# Synthesis of Antioxidative *p*-Terphenyl Dimers via Boronic Acid-Mediated C–C Coupling

**DOI:** 10.3390/ijms27062726

**Published:** 2026-03-17

**Authors:** Yong Wang, Yanchao Xu, Linmeng Chen, Dan Wu, Peng Fu, Liping Wang, Weiming Zhu

**Affiliations:** 1 Key Laboratory of Marine Drugs, Ministry Education of China, School of Medicine and Pharmacy, Ocean University of China, Qingdao 266003, China; wang0533yong@163.com (Y.W.); 17669470758@163.com (L.C.); fupeng@ouc.edu.cn (P.F.); 2 State Key Laboratory of Discovery and Utilization of Functional Components in Traditional Chinese Medicine, Guizhou Medical University, Guiyang 550014, China; xuyanchao@gmc.edu.cn (Y.X.); wudan@gmc.edu.cn (D.W.); 3 School of Pharmaceutical Sciences, Guizhou Medical University, Guiyang 561113, China; 4 Natural Products Research Center of Guizhou Province, Guiyang 550014, China; 5 Laboratory for Marine Drugs and Bioproducts, Marine Science and Technology Center, Qingdao 266237, China

**Keywords:** *p*-terphenyl dimers, boric acid, synthesis, antioxidation, α-glucosidase inhibition, PTP1B inhibition

## Abstract

By investigating the conditions for the C–C coupling reaction of *p*-terphenyls, we successfully synthesized C–C coupled dimeric *p*-terphenyls for the first time using a reaction system involving air, silica gel, and B(OH)_3_. Additionally, we developed a novel method to synthesize furan-fused *p*-terphenyl dimers through solvent-free reactions by creatively applying rotary evaporation and heating. Compounds **6**–**12**, **16**, **20**, and **22** demonstrated DPPH radical scavenging activity that was either stronger than or comparable to the positive control (vitamin C), with IC_50_ values ranging from 0.14 to 4.61 μM. Compounds **4**–**22** also exhibited significant activity against α-glucosidase, with IC_50_ values ranging from 0.37 to 17.9 μM, exceeding the efficacy of the positive control, acarbose. Moreover, compounds **6**–**14**, **16**–**18**, **21**, and **22** demonstrated greater inhibitory activity against PTP1B compared with the positive control, oleanolic acid, with IC_50_ values between 0.30 and 9.17 μM. These findings highlight their potential as promising leads or dietary supplements for the treatment and prevention of diabetes, as well as possible application as oxidative agents in food preservation.

## 1. Introduction

**Figure 1:**
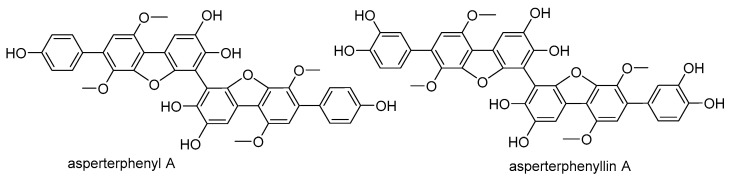
Structure of asperterphenyllin A and asperterphenyl A.

Research into *p*-terphenyl compounds, known for their antioxidant [1,2], cytotoxic [3,4,5], antibacterial [6], and α-glucosidase inhibitory [7,8,9] and protein tyrosine phosphatase 1B (PTP1B) inhibitory activities [10], dates back to 1877, with over 230 *p*-terphenyls documented to date [9,10,11,12,13,14]. Among these, only two dimeric *p*-terphenyl compounds, asperterphenyllin A [10] and asperterphenyl A [13], have been identified from natural sources (Figure 1). Notably, asperterphenyl A demonstrated stronger neuraminidase inhibitory activity and anti-influenza virus A (H1N1) activity compared with its monomer [13]. Additionally, there are limited reports on the synthesis of *p*-terphenyls [15,16,17,18,19,20], which significantly restricts their structural diversity.

**Scheme 1:**
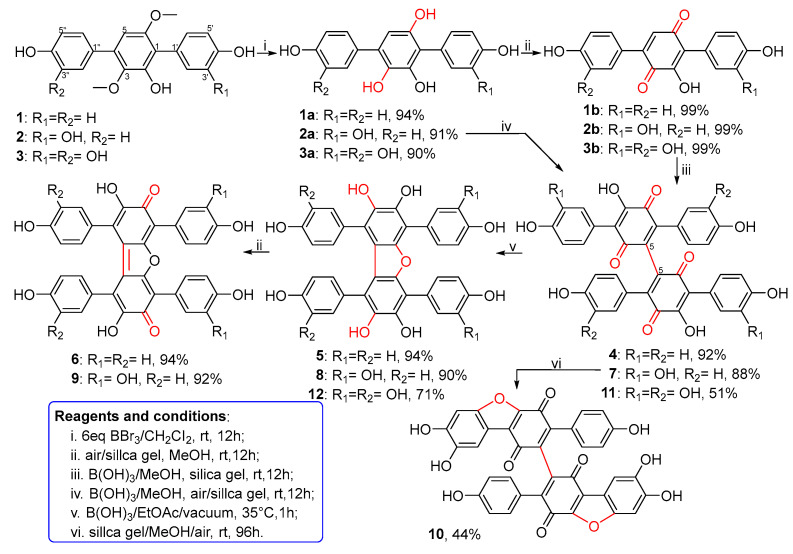
The synthesis of *p*-terphenyl dimers **4**–**12** from the monomers **1**–**3**.

**Scheme 2:**
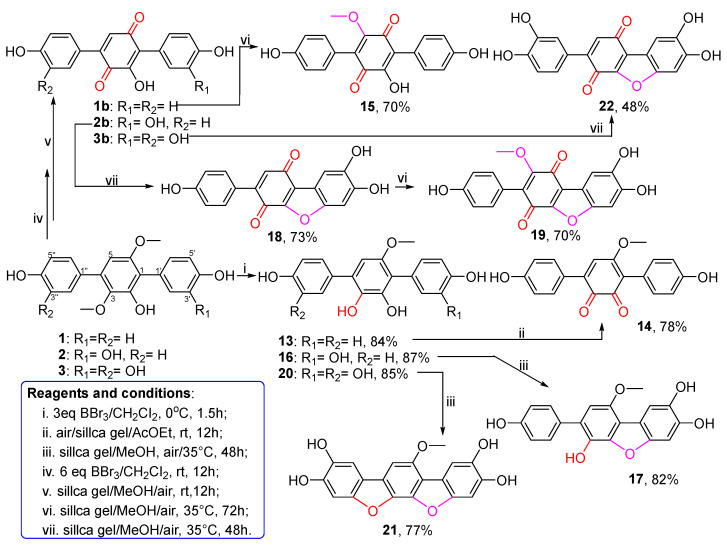
The synthesis of *p*-terphenyl monomers **13**–**22** from compounds **1**–**3**.

It has been established that antioxidant activity, as well as α-glucosidase and PTP1B inhibitory effects, are closely associated with diabetes. Diabetes has become a major global public health concern [21]. It is classified as a group of metabolic disorders resulting from an absolute or relative deficiency in insulin secretion, with hyperglycemia being the key indicator [22]. Research indicates that an imbalance in the antioxidant system can severely damage islet cells, leading to a reduction in insulin production [23,24,25]. α-Glucosidase inhibitors help reduce the body’s absorption of carbohydrates, while inhibiting PTP1B activity can improve the sensitivity of peripheral tissues to insulin, thus promoting insulin release [25,26,27]. In our previous research, we isolated three lower cytotoxic *p*-terphenyls, terphenyllin (**1**), 3′-hydroxyterphyllin (**2**), and 3′,3″-dihydroxyterphyllin (**3**) (Scheme 1), from the *Aspergillus* strain GZWMJZ-055 endophytic with the leaves of *Eucommia ulmoides*, a plant known for its homologous medicinal and food properties. These compounds demonstrated high content, along with significant antioxidant activity and α-glucosidase inhibition [16]. To continuously search for bioactive compounds derived from microbial metabolites and to enhance the structural diversity of *p*-terphenyls [25], we explored the C–C coupling reaction of *p*-terphenyl monomers **1**–**3**, the primary products of *Aspergillus* sp. GZWMJZ-055, using B(OH)_3_/silica gel. Our findings showed that 1,2,4-trihydroxy-*p*-terphenyls could be effectively transformed to 5,5-dimers (**4**–**12**), with promising yields ranging from 44% to 94% (Scheme 1). In addition, ten additional *p*-terphenyl derivatives (**13**–**22**) were also obtained in the yields of 48% to 87% (Scheme 2).

## 2. Results and Discussion

### 2.1. Dimerization of p-Terphenyls ***1**–**3***

To investigate the dimeric reaction of *p*-terphenyl monomers, we initially examined the *O*-demethylation of compound **1**. Using three equivalent (eqv) BBr_3_ and reacting for 1.5 h at 0 °C, we obtained only the 3-*O*-demethyl product **13**. However, by employing six eqv BBr_3_ and reacting for 12 h at room temperature (rt), both the 3-*O*-methyl and 6-*O*-methyl groups were removed, resulting in the formation of 1,2,4-trihydroxy-*p*-terphenyl (**1a**) with a satisfactory yield [16]. Under similar conditions, compounds **2** and **3** underwent 3-*O*-demethyl and 3,6-*O*-didemethyl reactions smoothly, yielding compounds **2a**, **3a**, **16**, and **20**, respectively (Scheme 1 and Scheme 2).

Interestingly, during the isolation of compounds **1a** and **13** using a silica gel column, we also obtained a small amount of compounds **1b**, **4**–**6**, **14**, and **15** (Scheme 1 and Scheme 2). Considering the oxidative dehydrogenation of silica gel in air, we realized that silica gel may play a crucial role in forming dimers **4**–**6** and quinone derivatives **1b**, **14**, and **15** [16]. Consequently, we carefully studied the reaction of compound **1a** in the presence of air and silica gel. When dissolved in methanol (MeOH) with 133 times (*w*/*w*) of silica gel, compound **1a** was rapidly converted into compound **1b** at room temperature.

However, compounds **4**–**6** and **15** were not detected until compound **1a** was fully transformed into compound **1b** at 12 h (Table 1, entry 1), suggesting that the dimeric reaction required quinone substrates. Upon continuous heating to 35 °C in the presence of silica gel and air, compound **1b** gradually transformed into compound **15** (Scheme 2). Nonetheless, even when the temperature was raised to 100 °C, the amount of silica gel increased to 500 times (*w*/*w*), and the reaction time extended to 7 d, compound **1b** did not transform to dimers **4**–**6**. Upon retrospective analysis, we realized that BBr_3_ could be hydrolyzed to produce boric acid (B(OH)_3_) and hydrobromic acid (HBr) during the reaction quenching process by adding water [28], and B(OH)_3_ might catalyze the C–C dimerization of quinone derivative **1b**. Therefore, we investigated the effects of silica gel, B(OH)_3_, and HBr on the formation of the dimer **4**. The results indicated that compound **4** was produced with high yields when B(OH)_3_ and silica gel were added to the MeOH solution of compound **1b**, irrespective of the presence of hydrobromic acid (HBr) (Table 1, entries 2 and 3). However, compound **4** was not formed from **1b** if B(OH)_3_ was used alone (Table 1, entry 4). Furthermore, the use of proton acids such as HBr or *p*-toluenesulfonic acid (TsOH) did not lead to the dimerization of compound **1b** into compound **4**, even in the presence of silica gel (Table 1, entries 5 and 6). In contrast, when other Lewis acids, like aluminum trichloride (AlCl_3_), were utilized alongside silica gel, compound **1b** was converted into compound **4**, albeit only in trace amounts (Table 1, entry 7). Specifically, compound **1a** can be employed directly for dimerization when performed in the presence of B(OH)_3_ and silica gel under air atmosphere (Table 1, entry 8; Scheme 1). Under the same conditions, intermediates **2a**/**2b** and **3a**/**3b** undergo the dimerization to form compounds **7** and **11**, respectively, with high yields (Scheme 1).

Next, we examined the conversion of compound **4** to compound **5**, which features a furan nucleus fused between two *p*-terphenyls. In our previous work, we used silica gel to synthesize a furan nucleus between two adjacent phenyls of the *p*-terphenyl, specifically in 3′-hydroxyterphyllin [16]. However, this cyclization reaction did not occur with compound **4** (Table 2, entry 1). In contrast, compounds **2b** and **3b** successfully underwent cyclization to produce compounds **18** and **22**, respectively (Scheme 2), highlighting that the formation of the furan ring necessitates the presence of a hydroxy group. Given the Lewis acid nature of B(OH)_3_, and its previous use in promoting the formation of 5-hydroxymethylfurfural from glucose [29], we explored the furan-ring-forming reaction of compound **4** using B(OH)_3_ and silica gel in various solvents. The results demonstrated that compound **5** remained undetectable even with increased amounts of B(OH)_3_ and silica gel, and a temperature elevation to 60 °C (Table 2, entries 2–4). Surprisingly, compound **5** was detected, albeit in a small amount (4% yield), after the solvent MeOH was removed using a rotary evaporator at 35 °C under reduced pressure (Table 2, entry 5). This suggests that the formation of the furan ring from *p*-terphenyl quinone (**4**) necessitates a solvent-free environment. Consequently, we dissolved compound **4** in MeOH with the addition of B(OH)_3_, followed by vacuum evaporation for 1 h at 35 °C on a rotary evaporator, resulting in the conversion of compound **4** to **5** with an 87% yield (Table 2, entry 6). Using EtOAc as the solvent further increased the yield to 94% (Table 2, entry 7). In contrast, no reaction occurred when CH_2_Cl_2_ was used as the solvent (Table 2, entry 8) or when no vacuum was employed using MeOH (Table 2, entry 9).

**Scheme 3:**
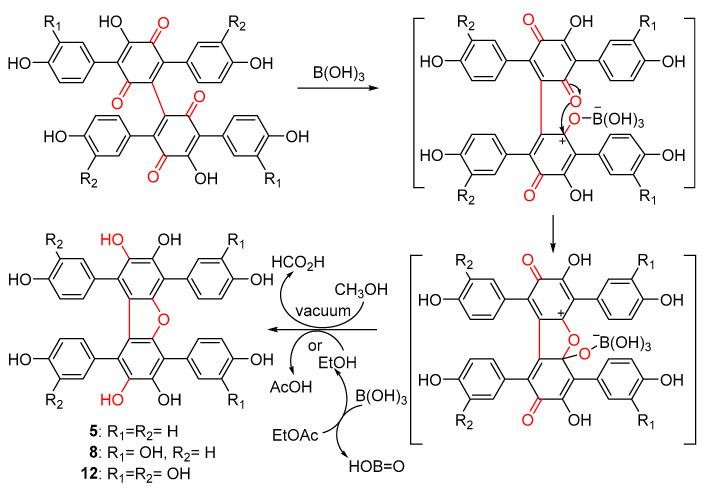
The postulated pathway for transforming dimeric *p*-quinones into furan-fused hydroquinone dimers.

This transformation could be attributed to the conversion of *p*-quinone (**4**) into the key furan-fused tetrahydroxyborate intermediate (**4a**) with the use of B(OH)_3_, followed by intramolecular reduction to hydroquinone (**5**) facilitated by MeOH or EtOAc under reduced pressure (Scheme 3). These optimal reaction conditions are also effective for compounds **7** and **11**, resulting in the high-yield production of compounds **8** and **12**, respectively (Scheme 1).

**Figure 2:**
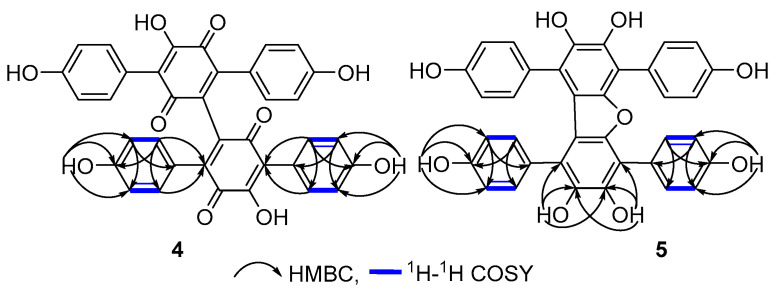
Selected COSY and HMBC correlations of compounds **4** and **5**.

Compound **4** was determined to have a molecular formula of C_36_H_22_O_10_, based on its HRESIMS data at *m*/*z* 613.1128 [M − H]^−^ (calcd. 613.1140 for C_36_H_21_O_10_^−^). Notably, the ^13^C NMR spectrum revealed only 18 carbon signals, suggesting that it is a symmetrical dimer. Additionally, the IR spectrum showed characteristic C=O stretching peaks of *p*-benzoquinone at ν_max_ 1654 and 1617 cm^−1^. The NMR data were highly similar to those of compound **1b** [16], with the main differences being the absence of the aromatic methine signals at *δ*_H/C_ 6.73 (s)/131.2 (CH-5) and the presence of a quaternary carbon signal at *δ*_C_ 139.6 (C-5) (Table 3). Additionally, distinct shifts were observed in the signals for the moderate phenyl ring and the adjacent carbons (C-1′ and C-1″). These findings suggest that compound **4** is the dimer of compound **1b**, linked by a single bond between two C-5 positions. This structure was further confirmed through the interpretation of 2D NMR spectra, particularly the COSY correlations between H-2′ and H-3′ as well as H-2″ and H-3″, along with the HMBC connections of H-2′/6′ to C-1′ and H-2″/6″ to C-1″ (Figure 2).

The molecular formula of compound **5** was determined to be C_36_H_24_O_9_ from its HRESIMS data at *m*/*z* 618.1745 [M + NH_4_]^+^ (calcd. 618.1759 for C_36_H_28_O_9_^+^), indicating that compound **5** has one less oxygen and two more hydrogens compared with compound **4**. Notably, the IR spectrum lacked the characteristic C=O stretching peaks typically observed for *p*-benzoquinone. The ^13^C NMR spectrum of **5** also displayed 18 signals; however, the signals corresponding to two carbonyl carbons were absent. Moreover, the NMR data for compound **5** were similar to compound **1a** [16], with the primary distinction being the absence of an exchangeable -OH signal and the presence of a quaternary carbon signal at *δ*_C_ 115.1 (C-5), which replaced the aromatic methine signals observed at *δ*_H/C_ 6.28 (s)/106.6 (CH-5) (Table 3). Additionally, significant shifts in the carbon signals for the moderate phenyl ring were noted. These findings suggest that compound **5** is a dimer of compound **1a**, connected by a furan nucleus between two C-5 positions and two 6-OH positions. This structure was confirmed by COSY and HMBC correlations that are nearly identical to those observed for compound **4** (Figure 2, Table 3).

Finally, when compounds **5**, **8**, and **12** were subjected to oxidation using air and silica gel, compounds **5** and **8** were successfully converted to compounds **6** and **9**, respectively (Scheme 1). However, the reaction involving compound **12** proved to be complex, resulting in no isolated products. Additionally, compound **7** underwent complete oxidation to yield the dimeric *p*-terphenyl derivative (**10**), which contains a furan nucleus, attributed to the presence of the *p*-hydroxy group (3′-OH) under air and silica gel conditions. Similarly, the reaction involving compound **11** also exhibited complexity, with no products isolated. Notably, all the *p*-terphenyl dimers **4**–**12** could be directly synthesized from compounds **1**–**3** without the need to purify all the intermediate products, achieving moderate to high yields.

**Scheme 4:**
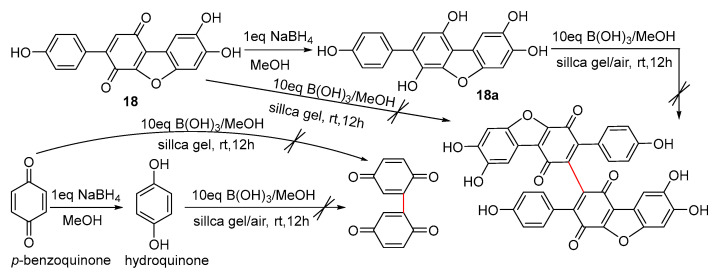
Attempts at C–C coupling of phenol compounds.

In the exploration of the dimerization of compounds **1**–**3**, we also obtained some new derivatives of the monomers. To evaluate the bioactivity, we also isolated and identified these compounds, **13**–**21** (Scheme 2). However, compounds **1**–**3**, along with **13**, **14**, **16**–**18**, **20**, and **21**, could not be directly transformed into the corresponding dimers under the conditions of air, silica gel, and B(OH)_3_ (Scheme 4). This indicates that benzene-1,2,4-triol serves as the fundamental framework for the C–C coupling reaction mediated by B(OH)_3_ and silica gel (Scheme 1).

**Scheme 5:**
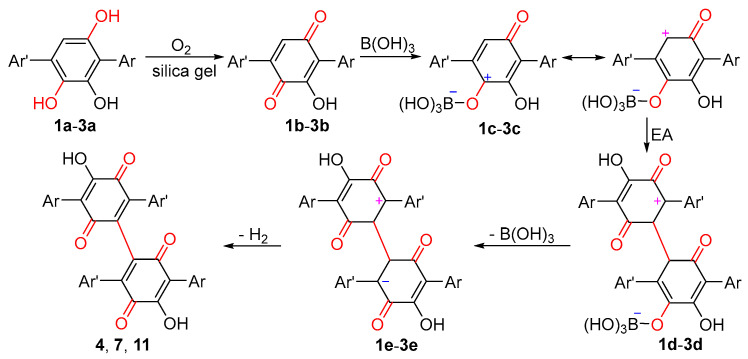
The proposed mechanism of C–C coupling of benzene-1,2,4-triol compounds.

We propose that benzene-1,2,4-triol compounds (**1a**–**3a**) undergo oxidation to yield 2-hyderoxy-1,4-benthoquinone compounds (**1b**–**3b**) in the presence of air and silica gel. These *p*-quinones (**1b**–**3b**) then interact with B(OH)_3_ to form complex boric acid intermediates (**1c**–**3c**). This complex subsequently undergoes electrophilic addition (EA) with another molecule of *p*-quinones (**1b**–**3b**), resulting in the formation of boric acid complex salt intermediates (**1d**–**3d**). Following this, the elimination of B(OH)_3_ occurs, leading to the generation of intramolecular salt intermediates (**1e**–**3e**). The *p*-terphenyl dimers **4**, **7**, and **11** are then obtained by the elimination of one molecule of hydrogen from intermediates (**1e**–**3e**). We speculated that silica gel acts as a carrier by adsorbing two molecules of *p*-terphenyl via hydrogen bonding, thereby bringing the C-5 positions into close spatial proximity, which facilitates C–C coupling (Scheme 5).

**Scheme 6:**
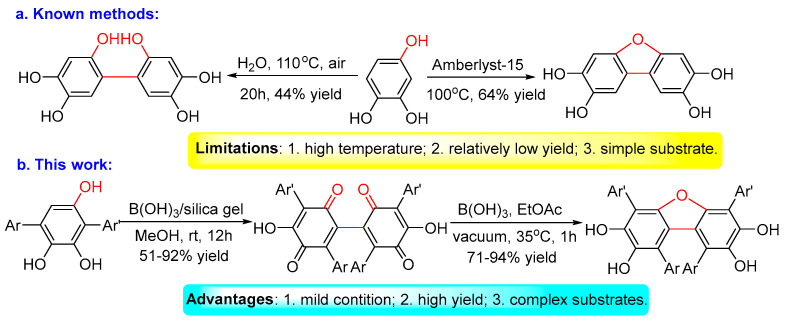
Advantage of B(OH)_3_/silica gel mediated C–C coupling of benzene-1,2,4-triol compounds.

Literature reviews reveal only two reports on the C–C coupling between two molecules of benzene-1,2,4-triol, which involves air-oxidized coupling [30] and acid-catalyzed dehydration [31]. to form a furan ring, achieving yields of 44–64%. However, these methods require high temperatures, specifically 100 or 110 °C (Scheme 6). In response to these limitations, we have developed a green and economical synthetic route for producing *p*-terphenyl dimers. This method involves C–C coupling, followed by the formation of a fused furan ring from *p*-terphenyl dimers derived from monomers that possess a benzene-1,2,4-triol structure, achieving yields ranging from moderate to high (51–94%).

The B(OH)_3_ and silica gel catalyzed C–C coupling reaction of 1,2,4-trihydroxy-*p*-terphenyls offers several advantages. It is environmentally friendly, as it avoids the use of heavy metal catalysts, thereby reducing environmental and health hazards. The process is cost-effective due to the use of readily available and inexpensive materials like B(OH)_3_ and silica gel. Additionally, it is energy efficient, as the reaction typically proceeds under milder conditions compared with traditional methods that require high temperatures or metal catalysis. The method also achieves moderate to high yields, enhancing its efficiency and practicality for potential industrial applications. Furthermore, the simplicity and accessibility of the reaction setup make it suitable for laboratory-scale synthesis without the need for complex equipment or procedures.

### 2.2. Bioactivities of Synthesized p-Terphenyls

The new phenolic compounds **4**–**22** were evaluated for their antioxidant activity using the DPPH radical scavenging assay [16] and the oxygen radical absorbance capacity (ORAC) method [16]. The results indicated that compounds **6**–**12**, **16**, **20**, and **22** exhibited DPPH radical scavenging activity that was either stronger than or comparable to the positive control (vitamin C, Vic), with IC_50_ values ranging from 0.14 to 4.61 μM. In contrast, compounds **17** and **21**, which contain a 2,6′-fused furan ring and 2,6′:3,6″-difused furan rings, respectively, displayed significantly reduced DPPH radical scavenging activity (Table 4). Furthermore, compounds **4**–**22** demonstrated significant antioxidant capacity, as evidenced by their oxygen radical absorbance capacity (ORAC) values, which ranged from 0.91 to 9.23 μM µmol TE/µmol (Table 4). This underscores their potential application in food preservation.

Compounds **4**–**22** were also evaluated for their potential in treating diabetes by assessing their inhibitory activities against α-glucosidase [16] and PTP1B (protein tyrosine phosphatase 1B) [25]. The results revealed that compounds **4**–**22** showed significant α-glucosidase inhibitory activity, with IC_50_ values ranging from 0.37 to 17.9 μM, surpassing the efficacy of the positive control, acarbose (AC) (Table 4). Notably, the dimeric *p*-terphenyls **6**, **10**, and **12** displayed stronger α-glucosidase inhibitory activity than monomers, with IC_50_ values below 1 μM. Among these, the furan-fused dimer **10** demonstrated the most potent α-glucosidase inhibition, achieving an IC_50_ of 0.37 μM. In addition, compounds **6**–**14**, **16**–**18**, **21**, and **22** exhibited greater PTP1B inhibitory activity compared with the positive control, oleanolic acid (OA), with IC_50_ values ranging from 0.30 to 9.17 μM. In contrast, compounds **4** and **15**, which have fewer than four hydroxy substitutions on the monomer units, showed poor activity against PTP1B (Table 4). This suggests that the presence of four or more hydroxy groups at the 3-, 3′-, 4′-, 3″-, and 4″-positions significantly enhances the PTP1B inhibitory activity of *p*-terphenyl derivatives. Unlike their α-glucosidase inhibitory profiles, the monomeric *p*-terphenyls showed stronger PTP1B inhibitory effects compared with the dimeric counterparts. Furthermore, comparison of compounds **18** (IC_50_ 0.47 μM) and **19** (12.4 μM) revealed that the 5-methoxy substitution significantly diminished the PTP1B inhibitory activity of *p*-terphenyl-3,6-dione derivatives. This reduction is likely due to the *ortho*-methoxy group hindering the formation of hydrogen bonds between the carbonyl oxygen and the PTP1B protein. Notably, compounds **13**, **14**, **18**, and **22**, which feature structural motifs such as 2,3-dihydroxy, 2,3-benzoquinone, or furan-fused 1,4-benzoquinone, exhibit the strongest activity against PTP1B, with IC_50_ values below 1 μM. These findings highlight their promise as potential leads or dietary supplements for the treatment and prevention of diabetes.

## 3. Materials and Methods

### 3.1. General Experimental Procedures

LC-MS data were acquired using a SHIMADZUR-R-smz-O1-LCMS-2020 HPLC/MS system (Shimadzu Corporation, Takamatsu, Japan) equipped with a reversed-phase C18 column (5 μm, 4.6 × 150 mm) at a flow rate of 1.0 mL/min. For detailed structural analysis, a Varian INOVA-400 MHz (Varian Associates, Palo Alto, CA, USA) and a Bruker 600 MHz superconducting nuclear magnetic resonance (NMR) spectrometer (Bruker, Fällanden, Switzerland) were employed. Infrared (IR) spectra were recorded on a Nicolet Nexus 470 spectrophotometer (Thermo Fisher Scientific, Waltham, MA, USA) using KBr discs. High-resolution electrospray ionization mass spectrometry (HRESIMS) was performed with a Q-TOF Ultima Global GAA076 LC mass spectrometer (Waters Corporation, Milford, MA, USA). Semi-preparative HPLC was conducted using an ODS column (YMC-pack ODS-A (YMC Corporation, Kyoto, Japan,), 10 × 250 mm, 5 μm, 4.0 mL/min). Thin-layer chromatography (TLC) and column chromatography (CC) were carried out on plates pre-coated with silica gel GF254 (10–40 μm) and on silica gel (200–300 mesh, Qingdao Marine Chemical Factory, Qingdao, China), as well as on Sephadex LH-20 (Amersham Biosciences, Uppsala, Sweden). Flash column chromatography (FCC) was executed using silica gel H from Qingdao Marine Chemical Factory.

### 3.2. Chemical Synthesis Procedures

#### 3.2.1. Synthesis of Compounds **1a**–**3a** and **1b**–**3b**

Compounds **1a**–**3a** and **1b**–**3b** were synthesized according to our previous report [16].

#### 3.2.2. Synthesis of the Dimeric *p*-Terphenyls **4**, **7**, and **11**

Compound **1** (1 g, 2.96 mmol) was dissolved in 75 mL of CH_2_Cl_2_, and BBr_3_ (26.3 mL, 17.76 mmol) was added to the solution. The mixture was stirred at room temperature for 12 h, and then 3 L of EtOAc was added. The solution obtained was washed three times with H_2_O (300 mL each), and the organic phase was then dried over anhydrous Na_2_SO_4_ and concentrated under reduced pressure. The resulting residue was dissolved in 330 mL of MeOH, and B(OH)_3_ (549 mg, 8.88 mmol), along with 133 g of 200–300 mesh silica gel, were added. The mixture was stirred at rt for another 12 h, after which the solvent was evaporated. The residue was purified by flash column chromatography (FCC) using a 1:1 (*v*/*v*) mixture of EtOAc-CH_2_Cl_2_ as the eluent, yielding compound **4** as a dark red solid with an R*_f_* value of 0.1 and a yield of 836 mg (92%). Using the same procedures, compound **7** (R*_f_* 0.1, 802 mg, 88% yield) was synthesized starting from compound **2** (1 g, 2.82 mmol) with the addition of BBr_3_ (25.1 mL, 16.92 mmol), B(OH)_3_ (1.22 g, 19.74 mmol), and silica gel (133 g). The product was purified by FCC with EtOAc-CH_2_Cl_2_ (*v*/*v* 2:1) as the eluent. In a similar manner, compound **11** (R*_f_* 0.1, 467 mg, 51% yield) was synthesized from compound **3** (1 g, 2.7 mmol) using BBr_3_ (24 mL, 16.2 mmol), B(OH)_3_ (1.67 g, 27 mmol), and silica gel (133 g), followed by purification via FCC with EtOAc-CH_2_Cl_2_ (*v*/*v* 2:1) as the eluent.

#### 3.2.3. Synthesis of the Dimeric *p*-Terphenyls **5**, **8** and **12**

A 20 mg (0.033 mmol) sample of pure or simply-purified compound **4** by FCC, obtained from the above synthesis, was dissolved in EtOAc (30 mL), followed by the addition of B(OH)_3_ (6.1 mg, 0.099 mmol). The reaction mixture was then rotated on a rotary evaporator at 35 °C for 1 h under vacuum. Subsequently, the reaction product was extracted with EtOAc (200 mL), and the organic layer was washed three times with H_2_O (20 mL each). The organic layer was then dried over anhydrous Na_2_SO_4_, and the solvent was removed under reduced pressure. The resulting residue was purified by semi-preparative HPLC using a YMC-pack ODS-A column (10 × 250 mm). The column was eluted with 50% MeOH/H_2_O containing 0.15% trifluoroacetic acid (TFA) at a flow rate of 4.0 mL/min, yielding compound **5** with a retention time (*t*_R_) of 10.3 min and a yield of 18.6 mg (94% yield). Using the same procedure, compounds **8** (*t*_R_ 9.7 min, 8.5 mg, 90% yield) and **12** (*t*_R_ 10.1 min, 7.1 mg, 71% yield) were respectively synthesized from compounds **7** (9.7 mg, 0.015 mmol) with B(OH)_3_ (6.5 mg, 0.105 mmol) and **11** (10.2 mg, 0.015 mmol) with B(OH)_3_ (9.3 mg, 0.15 mmol). Both products were purified by HPLC, which was eluted with 45% MeOH/H_2_O containing 0.15% TFA for **8** and 40% MeOH/H_2_O containing 0.15% TFA for **12**.

#### 3.2.4. Synthesis of the Dimeric *p*-Terphenyls **6** and **9**

Compound **5** (10 mg, 0.0167 mmol) was dissolved in MeOH (4 mL), and 1.3 g of silica gel (200–300 mesh) was added to the mixture. The reaction was allowed to proceed at room temperature for 12 h. Following this period, the MeOH was removed under vacuum, and the residue was purified by flash column chromatography (FCC), eluting with EtOAc-CH_2_Cl_2_ (*v*/*v* 1:5), which resulted in the isolation of compound **6** as a brown solid (R*_f_* 0.2, 9.4 mg, 94% yield). In a similar manner, compound **9** (R*_f_* 0.1, 4.6 mg, 92% yield) was synthesized by the reaction of compound **8** (5 mg, 0.0079 mmol) with 0.67 g of silica gel in MeOH, following the same procedures.

#### 3.2.5. Synthesis of the Dimeric *p*-Terphenyl **10**

Compound **7** (9.7 mg, 0.015 mmol) was dissolved in MeOH (4 mL), and 1.3 g of silica gel (200–300 mesh) was added to the solution. The mixture was stirred at room temperature for 96 h. After this period, the solvent was evaporated, and the resulting residue was purified using FCC, eluting with EtOAc-CH_2_Cl_2_ (*v*/*v* 1:5). This process yielded compound **10** (R*_f_* 0.1) as a dark red solid, with a final weight of 4.2 mg and a yield of 44%.

The physicochemical properties of the dimeric *p*-terphenyls **4**–**12**, along with the synthesis procedures for the monomeric *p*-terphenyls **13**–**22**, are detailed in the Supplementary Materials file. Additionally, this section includes the physicochemical properties of the monomeric *p*-terphenyls **13**–**22**, the MS spectra of compounds **1a**/b–**3a**/**b**, the MS and NMR spectra of compounds **4**–**22**, as well as the HPLC profile of compound **22** (Supplementary Figures S1–S70).

#### 3.2.6. Bioactivity Assays

According to our previous report on the activity study of *p*-terphenyls [16,25], compounds **4**–**22** were assessed for their antioxidant activities through the measurement of DPPH radical scavenging activity and oxygen radical absorbance capacity (ORAC) [16]. Additionally, their antidiabetic effects in vitro were evaluated by determining the inhibitory activities against PTP1B [25] and α-glucosidase from *Saccharomyces cerevisiae* [16], following previously established methods. Detailed protocols for these assays can be found in the Supplementary Materials file.

## 4. Conclusions

In summary, we have developed a mild, cost-effective, and environmentally friendly method for the gram-scale synthesis of *p*-terphenyl dimers. This procedure utilizes only B(OH)_3_, silica gel, and air, yet it yields satisfactory results for dimeric *p*-terphenyls **4**–**12**. Additionally, we synthesized ten new *p*-terphenyl monomers (**13**–**22**). The innovative application of rotary evaporation and heating offers a novel approach for solvent-free reactions in the synthesis of furan-fused *p*-terphenyl dimers **5**, **8**, and **12**. Several of the newly synthesized compounds exhibited significant DPPH radical scavenging effects and demonstrated inhibitory activities against α-glucosidase and PTP1B. These findings highlight their potential as promising leads or dietary supplements for the treatment and prevention of diabetes, as well as possible application as oxidative agents in food preservation. However, the paper lacks sufficient experimental evidence regarding the proposed C–C coupling reaction mechanism, which remains an important aspect to be addressed in our future research.

## Figures and Tables

**Table 1:** The synthetic conditions of compound **4** from **1a** and **1b**.

Entry	Compound	Conditions ^a^	Product (%)
1	**1a**	B(OH)_3_ or not, MeOH, rt, 12 h	–
2	**1b**	B(OH)_3_, HBr, MeOH, rt, 12 h	**4** (92%)
3	**1b**	B(OH)_3_, MeOH, rt, 12 h	**4** (92%)
4	**1b**	B(OH)_3_ ^b^, MeOH, rt, 12 h	–
5	**1b**	HBr, MeOH, rt, 12 h	–
6	**1b**	TsOH, MeOH, rt, 12 h	–
7	**1b**	AlCl_3_, MeOH, rt, 12 h	**4** (3%)
8	**1a**	B(OH)_3_, MeOH, air, rt, 12 h	**4** (92%)

^a^ With the addition of silica gel. ^b^ Without silica gel.

**Table 2:** The synthetic conditions of compound **5** from **4**.

Entry	Compound	Conditions	Product (%)
1	**4**	silica gel, MeOH, air, 60 °C, 12 h	0336
2	**4**	B(OH)_3_ ^a^, MeOH, air, 60 °C, 12 h	–
3	**4**	B(OH)_3_ ^a^, EtOAc, air, 60 °C, 12 h	–
4	**4**	B(OH)_3_ ^a^, CH_2_Cl_2_, air, 60 °C, 12 h	–
5	**4**	B(OH)_3_ ^a^, vacuum, MeOH, 35 °C, 1 h	**5** (4%)
6	**4**	B(OH)_3_, vacuum, MeOH, 35 °C, 1 h	**5** (87%)
7	**4**	B(OH)_3_, vacuum, EtOAc, 35 °C, 1 h	**5** (94%)
8	**4**	B(OH)_3_, vacuum, CH_2_Cl_2_, 35 °C, 1 h	–
9	**4**	B(OH)_3_, MeOH, air, 60 °C, 12 h	–

^a^ With the addition of silica gel.

**Table 3:** ^1^H (600 MHz) and ^13^C (150 MHz) NMR data of compounds **4** and **5** (DMSO-*d*_6_).

Position	4				5			
	*δ*_C_, Type	*δ*_H_, Mult (*J* in Hz)	COSY	HMBC	*δ*_C_, Type	*δ*_H_, Mult (*J* in Hz)	COSY	HMBC
1	121.0 C				112.0 C			
2	151.4 C				142.5 C			
3	182.6 C				139.8 C			
4	140.1 C				123.3 C			
5	139.6 C				115.1 C			
6	186.0 C				148.7 C			
1′	119.7 C				115.1 C			
2′	132.1 CH	7.11, d (8.6)	3′	1, 4′, 6′	132.1 CH	7.57, d (6.9)	3′	1, 4′, 6′
3′	114.4 CH	6.77, d (8.6)	2′	1′, 5′	115.1 CH	6.85, d (6.9)	2′	1′, 5′
4′	157.1 C				156.7 C			
5′	114.4 CH	6.77, d (8.6)	6′	1′, 3′	115.1 CH	6.85, d (6.9)	6′	1′, 3′
6′	132.1 CH	7.11, d (8.6)	5′	1, 2′, 4′	132.1 CH	7.57, d (6.9)	5′	1, 2′, 4′
1″	122.1 C				128.1 C			
2″	130.6 CH	6.60, d (8.6)	3″	4, 4″, 6″	131.6 CH	6.63, d (6.9)	3″	4, 4″, 6″
3″	114.7 CH	6.69, d (8.6)	2″	1″, 5″	114.4 CH	6.35, d (6.9)	2″	1″, 5″
4″	158.2 C				155.9 C			
5″	114.7 CH	6.69, d (8.6)	6″	1″, 3″	114.4 CH	6.63, d (6.9)	6″	1″, 3″
6″	130.6 CH	6.60, d (8.6)	5″	4, 2″, 4″	131.6 CH	6.35, d (6.9)	5″	4, 2″, 4″
2-OH		10.78, s				8.39, s		1, 2, 3
3-OH						7.40, s		2, 3, 4
4′-OH		9.61, s		3′, 4′, 5′		9.48, s		3′, 4′, 5′
4″-OH		9.77, s		3″, 4″, 5″		8.99, s		3″, 4″, 5″

**Table 4:** Anti-DPPH, α-glucosidase and PTP1B activities of compounds **4**–**22** (IC_50_, μM).

Compound	DPPH	ORAC ^a^	α-Glucosidase ^b^	PTP1B
**4**	5.69 ± 0.07	9.23 ± 0.22	6.86 ± 0.21	>50
**5**	7.00 ± 0.13	2.76 ± 0.13	2.54 ± 0.02	14.31 ± 0.09
**6**	2.56 ± 0.03	4.44 ± 0.09	0.71 ± 0.04	8.29 ± 0.11
**7**	0.80 ± 0.00	8.16 ± 0.21	3.53 ± 0.07	1.14 ± 0.04
**8**	1.03 ± 0.01	3.14 ± 0.13	1.34 ± 0.01	5.77 ± 0.00
**9**	1.80 ± 0.01	2.03 ± 0.11	1.59 ± 0.03	6.45 ± 0.11
**10**	1.85 ± 0.00	1.44 ± 0.02	0.37 ± 0.00	2.96 ± 0.02
**11**	1.55 ± 0.03	2.09 ± 0.12	1.87 ± 0.03	1.95 ± 0.03
**12**	0.89 ± 0.02	1.64 ± 0.05	0.66 ± 0.01	4.14 ± 0.04
**13**	13.23 ± 0.37	5.42 ± 0.18	6.40 ± 0.15	0.73 ± 0.01
**14**	25.38 ± 0.86	8.33 ± 0.32	4.60 ± 0.10	0.30 ± 0.02
**15**	9.68 ± 0.15	7.55 ± 0.04	6.62 ± 0.14	>50
**16**	0.20 ± 0.00	4.91 ± 0.13	4.90 ± 0.86	9.17 ± 0.17
**17**	>500	4.11 ± 0.02	2.90 ± 0.02	2.34 ± 0.05
**18**	8.90 ± 0.29	2.43 ± 0.04	2.83 ± 0.07	0.47 ± 0.00
**19**	5.18 ± 0.11	2.02 ± 0.01	3.37 ± 0.01	12.44 ± 0.17
**20**	0.14 ± 0.00	1.77 ± 0.01	6.50 ± 0.02	14.04 ± 0.15
**21**	>500	0.91 ± 0.11	17.9 ± 0.82	1.74 ± 0.02
**22**	4.61 ± 0.11	6.10 ± 0.00	1.47 ± 0.07	0.40 ± 0.03
Vic ^c^	4.76 ± 0.07	-	-	-
AC ^d^	-	-	220.67 ± 10.20	-
OA ^e^	-	-	-	11.34 ± 0.27

^a^ μmole TE/μmole (TE: trolox equivalents). ^b^ From *Saccharomyces cerevisiae*. ^c^ Vitamin C. ^d^ Acarbose. ^e^ Oleanolic acid.

## Data Availability

The original contributions presented in this study are included in the article/Supplementary Materials. Further inquiries can be directed to the corresponding authors.

## Supplementary Materials

The following supporting information can be downloaded at: https://www.mdpi.com/article/10.3390/ijms27062726/s1. Refs. [16,32,33,34,35,36] are cited in Supplementary Materials file.

## Abbreviations

CH_2_Cl_2_	Dichloromethane
MeOH	Methanol
BBr_3_	Boron Tribromide
B(OH)_3_	Boric acid
NaBH_4_	Sodium Borohydride
EtOAc	Ethyl Acetate
Na_2_SO_4_	Sodium Sulfate
DPPH	1,1-Diphenyl-2-picryl hydrazyl
LC-MS	Liquid Chromatography Mass Spectrometry
HPLC	High-Performance Liquid Chromatography
HRESI-MS	High-resolution electrospray ionization mass spectroscopy
rt	Room temperature
2D NMR	Two-Dimensional Nuclear Magnetic Resonance
HMBC	Heteronuclear Multiple Bond
COSY	Correlation Spectroscopy
*m*/*z*	Mass to Charge Ratio
R_f_	Retention Factor
IC_50_	Half Maximal Inhibitory Concentration
μM	Micromoles per liter
