# Supplementary material for: Synthesis of Antioxidative p-Terphenyl Dimers via Boronic Acid-Mediated C–C Coupling
Source: Int J Mol Sci. 2026 Mar 17;27(6):2726. doi: 10.3390/ijms27062726 (PMC13027276; doi:10.3390/ijms27062726)

Supporting Materials

Synthesis of Antioxidative *p*-Terphenyl Dimers via Boronic acid-mediated C–C Coupling

Yong Wang,[1,†] Yanchao Xu,[2,3,†] Linmeng Chen,[1] Dan Wu,[2,4] Peng Fu,[1,5] Liping Wang,[2,4,*] and Weiming Zhu [1,5,*]

[1] *Key Laboratory of Marine Drugs, Ministry Education of China, School of Medicine and Pharmacy, Ocean University of China, Qingdao 266003, China*

[2] *State Key Laboratory of Discovery and Utilization of Functional Components in Traditional Chinese Medicine, Guizhou Medical University, Guiyang 550014, China*

[3] *School of Pharmaceutical Sciences, Guizhou Medical University, Guiyang 561113, China*

[4] *Natural Products Research Center of Guizhou Province, Guiyang 550014, China*

[5] *Laboratory for Marine Drugs and Bioproducts, Marine Science and Technology Center, Qingdao 266237, China*

¶ These authors contributed equally to the work.

* Corresponding authors. E-mail: wangliping2022@gmc.edu.cn (L.W.), weimingzhu@ouc.edu.cn (W.Z.).

List of supporting information

General experimental procedures S4
Protocols for synthesis of compounds S4
Synthesis and characterization of compounds **1a–3a** S4
Synthesis and characterization of compounds **1b–3b** S5
Synthesis and characterization of compounds **4**, **7** and **11** S5
Synthesis and characterization of compounds **5, 8** and **12** S6
Synthesis and characterization of compounds **6**, **9** and **10** S8
Synthesis and characterization of compounds **15**, **18** and **22** S9
Synthesis and characterization of compounds **13**, **16, 19** and **20** S10
Synthesis and characterization of compounds **14, 17** and **21** S11
Bioactivity assay procedures S12
References S14

Figure S1. The LC-MS spectrum of compound **1a** S15
Figure S2. The LC-MS spectrum of compound **1b**........ S15
Figure S3. The LC-MS spectrum of compound **2a** S15
Figure S4. The LC-MS spectrum of compound **2b** S16
Figure S5. The LC-MS spectrum of compound **3a** S16
Figure S6. The LC-MS spectrum of compound **3b** S16
Figure S7. The ^{1}H (600 MHz) NMR spectrum of compound **4** in DMSO-d_6 .... S17
Figure S8. The ^{13}C (150 MHz) NMR spectrum of compound **4** in DMSO-d_6 .... S17
Figure S9. The HSQC spectrum of compound **4** in DMSO-d_6........ S18
Figure S10. The ^{1}H-^{1}H COSY spectrum of compound **4** in DMSO-d_6 .... S18
Figure S11. The HMBC spectrum of compound **4** in DMSO-d_6........ S19
Figure S12. The HRESIMS spectrum of compound **4** S19
Figure S13. The ^{1}H (600 MHz) NMR spectrum of compound **5** in DMSO-d_6 .... S20
Figure S14. The ^{13}C (150 MHz) NMR spectrum of compound **5** in DMSO-d_6 .... S20
Figure S15. The HSQC spectrum of compound **5** in DMSO-d_6........ S21
Figure S16. The ^{1}H-^{1}H COSY spectrum of compound **5** in DMSO-d_6 .... S21
Figure S17. The HMBC spectrum of compound **5** in DMSO-d_6........ S22
Figure S18. The HRESIMS spectrum of compound **5** S22
Figure S19. The ^{1}H (600 MHz) NMR spectrum of compound **6** in DMSO-d_6 .... S23
Figure S20. The ^{13}C (150 MHz) NMR spectrum of compound **6** in DMSO-d_6 .... S23
Figure S21. The HRESIMS spectrum of compound **6** S24
Figure S22. The ^{1}H (600 MHz) NMR spectrum of compound **7** in DMSO-d_6 .... S24
Figure S23. The ^{13}C (150 MHz) NMR spectrum of compound **7** in DMSO-d_6 .... S25
Figure S24. The HRESIMS spectrum of compound **7** S25
Figure S25. The ^{1}H (600 MHz) NMR spectrum of compound **8** in DMSO-d_6 .... S26
Figure S26. The ^{13}C (150 MHz) NMR spectrum of compound **8** in DMSO-d_6 .... S26
Figure S27. The HRESIMS spectrum of compound **8** S27
Figure S28. The ^{1}H (600 MHz) NMR spectrum of compound **9** in DMSO-d_6 .... S27
Figure S29. The ^{13}C (150 MHz) NMR spectrum of compound **9** in DMSO-d_6 .... S28
Figure S30. The HRESIMS spectrum of compound **9** S28
Figure S31. The ^{1}H (400 MHz) NMR spectrum of compound **10** in DMSO-d_6 .... S29
Figure S32. The ^{13}C (150 MHz) NMR spectrum of compound **10** in DMSO-d_6 .... S29
Figure S33. The HRESIMS spectrum of compound **10** S30
Figure S34. The ^{1}H (600 MHz) NMR spectrum of compound **11** in DMSO-d_6 .... S30
Figure S35. The ^{13}C (150 MHz) NMR spectrum of compound **11** in DMSO-d_6 .... S31
Figure S36. The HRESIMS spectrum of compound **11** S31
Figure S37. The ^{1}H (600 MHz) NMR spectrum of compound **12** in DMSO-d_6 .... S32
Figure S38. The ^{13}C (150 MHz) NMR spectrum of compound **12** in DMSO-d_6 .... S32
Figure S39. The HRESIMS spectrum of compound **12** S33
Figure S40. The ^{1}H (600 MHz) NMR spectrum of compound **13** in DMSO-d_6 .... S33
Figure S41. The ^{13}C (150 MHz) NMR spectrum of compound **13** in DMSO-d_6 .... S34
Figure S42. The LC-MS spectrum of compound **13** S34
Figure S43. The HRESIMS spectrum of compound **14** in DMSO-d_6........ S35
Figure S44. The ^{1}H (600 MHz) NMR spectrum of compound **14** in DMSO-d_6 .... S35

Figure S45. The ^{13}C (150 MHz) NMR spectrum of compound **14** S36
Figure S46. The ^{1}H (600 MHz) NMR spectrum of compound **15** in DMSO-d_6 .... S36
Figure S47. The ^{13}C (150 MHz) NMR spectrum of compound **15** in DMSO-d_6 .... S37
Figure S48. The LC-MS spectrum of compound **15** S37
Figure S49. The ^{1}H (600 MHz) NMR spectrum of compound **16** in DMSO-d_6 .... S38
Figure S50. The ^{13}C (150 MHz) NMR spectrum of compound **16** in DMSO-d_6 .... S38
Figure S51. The HRESIMS spectrum of compound **16** S39
Figure S52. The ^{1}H (600 MHz) NMR spectrum of compound **17** in DMSO-d_6 .... S39
Figure S53. The ^{13}C (150 MHz) NMR spectrum of compound **17** in DMSO-d_6 .... S40
Figure S54. The HRESIMS spectrum of compound **17** S40
Figure S55. The ^{1}H (600 MHz) NMR spectrum of compound **18** in DMSO-d_6 .... S41
Figure S56. The ^{13}C (150 MHz) NMR spectrum of compound **18** in DMSO-d_6 .... S41
Figure S57. The HRESIMS spectrum of compound **18** S42
Figure S58. The ^{1}H (600 MHz) NMR spectrum of compound **19** in DMSO-d_6 .... S42
Figure S59. The ^{13}C (150 MHz) NMR spectrum of compound **19** in DMSO-d_6 .... S43
Figure S60. The HRESIMS spectrum of compound **19** S43
Figure S61. The ^{1}H (600 MHz) NMR spectrum of compound **20** in DMSO-d_6 .... S44
Figure S62. The ^{13}C (150 MHz) NMR spectrum of compound **20** in DMSO-d_6 .... S44
Figure S63. The HRESIMS spectrum of compound **20** S45
Figure S64. The ^{1}H (600 MHz) NMR spectrum of compound **21** in DMSO-d_6 .... S45
Figure S65. The ^{13}C (150 MHz) NMR spectrum of compound **21** in DMSO-d_6 .... S46
Figure S66. The HRESIMS spectrum of compound **21** S46
Figure S67. The ^{1}H (600 MHz) NMR spectrum of compound **22** in DMSO-d_6 .... S47
Figure S68. The ^{13}C (150 MHz) NMR spectrum of compound **22** in DMSO-d_6 .... S47
Figure S69. The HRESIMS spectrum of compound **22** S48
Figure S70. The HPLC spectrum of compound **22** S48

General experimental procedures

NMR spectra were recorded on a Bruker AVANCE NEO 400 spectrometer, or JEOL JNM-ECP 600 spectrometer, and chemical shifts were referenced to the corresponding residual solvent signals ($\delta_{H/C}$ 2.50/39.52 for DMSO-d_6); LC-MS data were obtained on a SHIMADZUR-R-smz-O1-LCMS-2020 HPLC/MS system; High resolution electrospray ionization mass spectrometer (Thermo Finniga, USA, Agilent-Technologies 6530-precisive-mass-Q-TOF-LC/M); High performance liquid chromatograph for analysis and preparation (Hitachi, Primaide); Silica gel H for column chromatography and thin layer chromatography (Qingdao Ocean Chemical Group Corporation, 10–40 μm); Chromatographic column (Beijing Xinwell Glass Instrument Company); Electronic analytical balance (Shanghai Tianping Instrument Factory, Model FA1104).

Protocols for synthesis of compounds

Synthesis of compounds 1a–3a

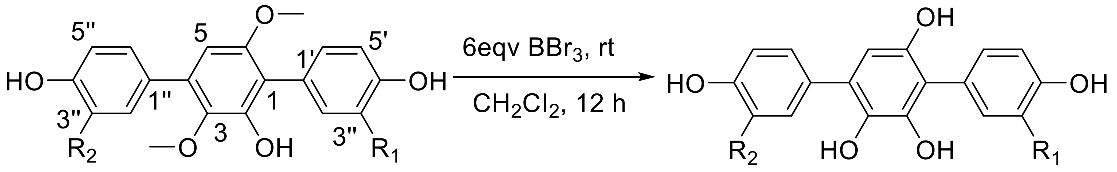

1: R_1=R_2= H
2: R_1= OH, R_2= H
3: R_1=R_2= OH

1a: R_1=R_2= H
2a: R_1= OH, R_2= H
3a: R_1=R_2= OH

According to our previous report, [S1] compound **1** (60 mg, 0.18 mmol) was dissolved in CH_2Cl_2 (10 mL), followed by the addition of BBr_3 (1.6 mL, 1.08 mmol). The mixture was stirred for 12 h at rt. EtOAc (200 mL) was then added, and the organic layer was washed three times with H_2O (20 mL each). The organic layer was dried over anhydrous Na_2SO_4 and concentrated in vacuo. The residue was purified by semipreparative HPLC on an ODS-A column (YMC-pack ODS-A, 10×250 mm), eluting with 50% MeOH/H_2O supplemented with 0.15% TFA at a flow rate of 4.0 mL/min, to provide **1a** (t_R = 7.5 min, 52.1 mg, 94% yield). Following the same procedures, compound **2a** (t_R = 8.0 min, 50.4 mg, 91% yield) was synthesized from compound **2** (60 mg, 0.17 mmol) with BBr_3 (1.52 mL, 0.102 mmol) and purified by HPLC, eluting with 40% MeOH/H_2O supplemented with 0.15% TFA at a flow rate of 4.0 mL/min. Similarly, compound **3a** (t_R = 7.1 min, 49.6 mg, 90% yield) was synthesized from compound **3** (60 mg, 0.162 mmol) with BBr_3 (1.44 mL, 0.096 mmol) and purified by HPLC, eluting with 30% MeOH/H_2O supplemented with 0.15% TFA at a flow rate of 4.0 mL/min.

p-Terphenyl-2,3,6,4',4"-pentaol (**1a**): LC-MS *m/z* 311.15 $[M+H]^+$; Exchangeable proton signals (600 MHz, DMSO-d_6) δ_H 9.39 (s, 1H, HO-4"), 9.25 (s, 1H, HO-4'), 8.46 (s, 1H, HO-2), 7.88 (s, 1H, HO-6), 7.54 (s, 1H, HO-3). [S1]

p-Terphenyl-2,3,6,3',4',4"-hexaol (**2a**): LC-MS *m/z* 327.15 $[M+H]^+$.

p-Terphenyl-2,3,6,3',4',3",4"-heptaol (**3a**): LC-MS *m/z* 343.10 $[M+H]^+$.

Synthesis of compounds 1b–3b

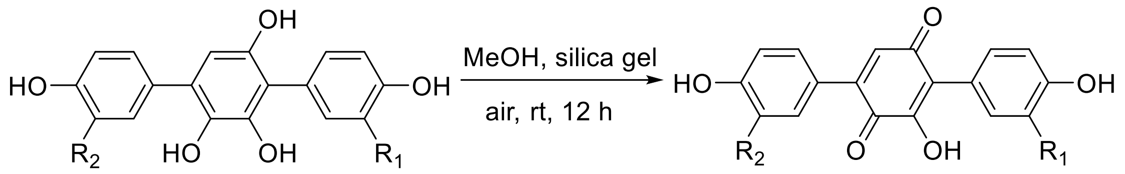

1a: R_1=R_2= H
2a: R_1= OH, R_2= H
3a: R_1=R_2= OH

1b: R_1=R_2= H
2b: R_1= OH, R_2= H
3b: R_1=R_2= OH

According to our previous report, [S1] compound **1a** (22 mg, 0.071 mmol) was dissolved in MeOH (7 mL), followed by the addition of 200−300 mesh silica gel (2.9 g). The mixture was stirred for 12 h at rt, and the solvent was evaporated. The residue was purified by flash column chromatography (FCC), eluting with EtOAc-CH_2Cl_2 (v/v 1:1) to give compound **1b** (R_f = 0.2, 21.6 mg, 99% yield). Following the same procedures, compound **2b** (R_f = 0.3, 19.6 mg, 99% yield) was synthesized from compound **2a** (20 mg, 0.061 mmol) with 200−300 mesh silica gel (2.7 g), and purified by flash column chromatography (FCC), eluting with EtOAc-CH_2Cl_2 (v/v 2:1). Similarly, compound **3b** (R_f = 0.2, 18.8 mg, 99% yield) was synthesized from compound **3a** (19 mg, 0.056 mmol) with 200−300 mesh silica gel (2.5 g), and purified by flash column chromatography (FCC), eluting with EtOAc-CH_2Cl_2 (v/v 4:1).

2,4',4''-Trihydroxy-*p*-terphenyl-3,6-dione (**1b**): LC-MS *m/z* 309.05 $[M+H]^+$;

2,3',4',4''-Tetrahydroxy-*p*-terphenyl-3,6-dione (**2b**): LC-MS *m/z* 325.10 $[M+H]^+$;

2,3',4',3'',4''-Pentahydroxy-*p*-terphenyl-3,6-dione (**3b**): LC-MS *m/z* 341.05 $[M+H]^+$.

Synthesis of compounds 4, 7 and 11

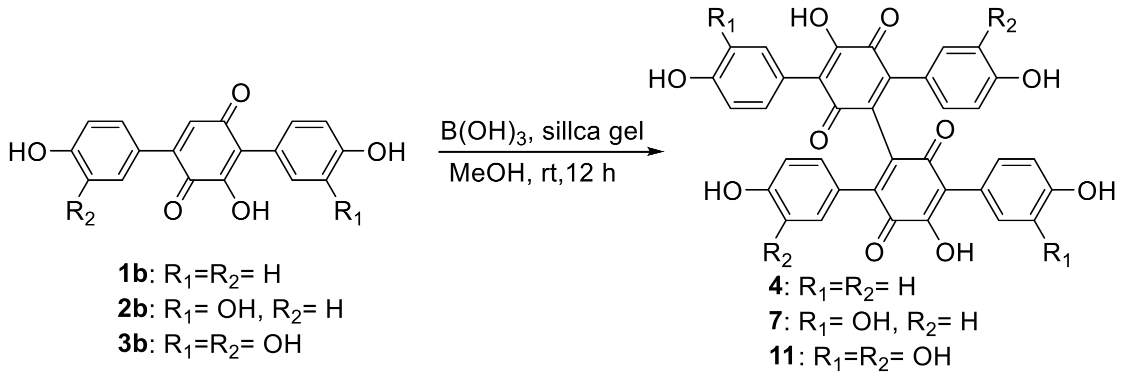

1b: R_1=R_2= H
2b: R_1= OH, R_2= H
3b: R_1=R_2= OH

4: R_1=R_2= H
7: R_1= OH, R_2= H
11: R_1=R_2= OH

Compound **1b** (9.9 mg, 0.032 mmol) was dissolved in MeOH (10 mL), followed by the addition of $B(OH)_3$ (5.9 mg, 0.096 mmol) and 200−300 mesh silica gel (1.33 g). Under an argon atmosphere, the mixture was stirred for 12 h at rt, and the solvent was evaporated. The residue was purified by flash column chromatography (FCC), eluting with EtOAc-CH_2Cl_2 (v/v 1:1) to give compound **4** (R_f = 0.1, 9.0 mg, 92% yield). Following the same procedures, compound **7** (R_f = 0.1, 8.8 mg, 88% yield) was synthesized from compound **2b** (10 mg, 0.031 mmol)

with $B(OH)_3$ (13.6 mg, 0.22 mmol) and 200−300 mesh silica gel (1.33 g), and purified by flash column chromatography (FCC), eluting with EtOAc-CH_2Cl_2 (v/v 2:1). Similarly, compound **11** (R_f = 0.1, 5.2 mg, 51% yield) was synthesized from compound **3b** (10.2 mg, 0.03 mmol) with $B(OH)_3$ (18.5 mg, 0.3 mmol) and 200−300 mesh silica gel (1.33 g), and purified by flash column chromatography (FCC), eluting with EtOAc-CH_2Cl_2 (v/v 3:1).

5,5-Bis(2,4',4''-trihydroxy-*p*-terphenyl-3,6-dione)(**4**): a dark red solid. m.p. >250 °C; IR (KBr) ν_{max} 3011, 2807, 2679, 1653, 1608, 1514, 1437, 1362, 1330, 1273, 1174, 1124, 952 cm^{-1}. ^1H NMR (600 MHz, DMSO-d_6) δ 10.78 (s, 1H), 9.77 (s, 1H), 9.61 (s, 1H), 7.11 (d, J = 8.6 Hz, 2H), 6.77 (d, J = 8.6 Hz, 2H), 6.69 (d, J = 8.6 Hz, 2H), 6.60 (d, J = 8.6 Hz, 2H); ^{13}C NMR (150 MHz, DMSO-d_6) δ 186.0, 182.6, 158.2, 157.1, 151.4, 140.1, 139.6, 132.1 (2×C), 130.6 (2×C), 122.1, 121.0, 119.7, 114.7 (2×C), 114.4 (2×C). HRESIMS *m/z* 613.1128 $[M–H]^-$ (calcd for $C_{36}H_{21}O_{10}^-$, 613.1140).

5,5-Bis(2,3',4',4''-tetrahydroxy-*p*-terphenyl-3,6-dione) (**7**): a dark red solid. m.p. >250 °C; IR (KBr) ν_{max} 3351, 2923, 1654, 1607, 1513, 1468, 1435, 1367, 1277, 1173, 1121, 1011 cm^{-1}. ^1H NMR (600 MHz, DMSO-d_6) δ 10.71 (s, 1H), 9.77 (s, 1H), 9.07 (s, 1H), 8.94 (s, 1H), 6.82 (d, J = 1.9 Hz, 1H), 6.74 (d, J = 8.2 Hz, 1H), 6.67 (d, J = 8.6 Hz, 2H), 6.61 (dd, J = 8.2, 1.9 Hz, 1H), 6.52 (d, J = 8.6 Hz, 2H). ^{13}C NMR (150 MHz, DMSO-d_6) δ 186.3, 182.7, 158.2, 151.6, 145.4, 144.3, 139.9, 139.8, 130.6 (2×C), 122.6, 122.0, 121.4, 119.9, 118.3, 114.9, 114.7 (2×C). HRESIMS *m/z* 645.10321 $[M–H]^-$ (calcd for $C_{36}H_{21}O_{12}^-$, 645.10275).

5,5-Bis(2,3',4',3'',4''-pentahydroxy-*p*-terphenyl-3,6-dione) (**11**): a dark red solid. m.p. >250 °C; IR (KBr) ν_{max} 3369, 1752, 1735, 1654, 1617, 1521, 1437, 1275, 1114, 1015 cm^{-1}. ^1H NMR (600 MHz, DMSO-d_6) δ 10.64 (s, 1H), 9.64–8.18 (m, 4H), 6.81 (d, J = 2.0 Hz, 1H), 6.72 (d, J = 8.2 Hz, 1H), 6.65 (d, J = 8.2 Hz, 1H), 6.49 (dd, J = 8.2, 2.0 Hz, 1H), 6.35 (d, J = 2.0 Hz, 1H), 6.10 (dd, J = 8.2, 2.0 Hz, 1H). ^{13}C NMR (150 MHz, DMSO-d_6) δ 185.8, 182.7, 151.2, 146.4, 145.4, 144.7, 144.3, 140.0, 139.6, 122.7, 122.6, 121.5, 120.9, 119.9, 118.4, 116.7, 114.9, 114.8. HRESIMS *m/z* 677.0950 $[M–H]^-$ (calcd for $C_{36}H_{21}O_{14}^-$, 677.0937).

Synthesis of compounds 5, 8 and 12

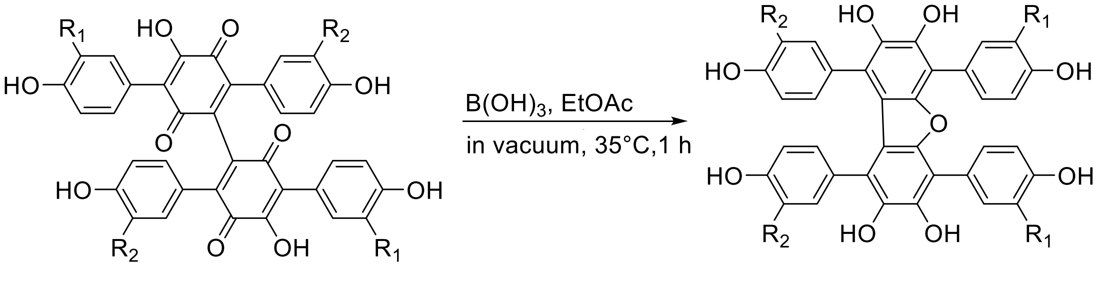

4: R_1=R_2=H
7: R_1=OH,R_2=H
11: R_1=R_2=OH

5: R_1=R_2=H
8: R_1=OH,R_2=H
12: R_1=R_2=OH

Compound **4** (20 mg, 0.033 mmol) was dissolved in EtOAc (30 mL), followed by the addition of $B(OH)_3$ (6.1 mg, 0.099 mmol). The mixture reacted for 1h in vocuum at 35 °C. and then EtOAc (200 mL) was added, and the organic layer was washed three times with H_2O (20 mL each). The organic layer was dried over anhydrous

Na_2SO_4 and concentrated in vacuo. The residue was purified by semipreparative HPLC on an ODS-A column (YMC-pack ODS-A, 10×250 mm), eluting with 50% MeOH/H_2O supplemented with 0.15% TFA at a flow rate of 4.0 mL/min, to provide **5** (t_R = 10.3 min, 18.6 mg, 94% yield). Following the same procedures, compound **8** (t_R = 9.7 min, 8.5 mg, 90% yield) was synthesized from compound **7** (9.7 mg, 0.015 mmol) with $B(OH)_3$ (6.5 mg, 0.105 mmol), and purified by HPLC, eluting with 45% MeOH/H_2O supplemented with 0.15% TFA at a flow rate of 4.0 mL/min. Similarly, compound **12** (t_R = 10.1 min, 7.1 mg, 71% yield) was synthesized from compound **11** (10.2 mg, 0.015 mmol) with $B(OH)_3$ (9.3 mg, 0.15 mmol), and purified by HPLC, eluting with 40% MeOH/H_2O supplemented with 0.15% TFA at a flow rate of 4.0 mL/min.

1,4,6,9-Tetra(4-hydroxyphenyl)dibenzo[*b*,*d*]furan-2,3,7,8-tetraol (**5**): a brown solid. m.p. >250 °C; IR (KBr) ν_{max} 3401, 2923, 1611, 1513, 1437, 1384, 1312, 1206, 1175, 1016 cm^{-1}. ^1H NMR (600 MHz, DMSO-d_6) δ 9.48 (s, 1H), 8.99 (s, 1H), 8.39 (s, 1H), 7.57 (d, J = 6.9 Hz, 2H), 7.40 (s, 1H), 6.85 (d, J = 6.9 Hz, 2H), 6.63 (d, J = 6.9 Hz, 2H), 6.35 (d, J = 6.9 Hz, 2H). ^{13}C NMR (150 MHz, DMSO-d_6) δ 156.7, 155.9, 148.7, 142.5, 139.8, 132.1 (2×C), 131.6 (2×C), 128.1, 124.0, 123.3, 115.1, 115.1 (2×C), 114.4 (2×C), 112.0. HRESIMS *m/z* 618.1745 $[M+NH_4]^+$ (calcd for $C_{36}H_{28}O_9N^+$, 618.1759).

4,6-Bis(3,4-dihydroxyphenyl)-1,9-bis(4-hydroxyphenyl)dibenzo[*b*,*d*]furan-2,3,7,8-tetraol (**8**): a brown solid. m.p. >250 °C; IR (KBr) ν_{max} 3421, 2923, 1752, 1617, 1561, 1512, 1437, 1385, 1122, 1014 cm^{-1}. ^1H NMR (600 MHz, DMSO-d_6) δ 8.99 (s, 1H), 8.91 (s, 1H), 8.86 (s, 1H), 8.35 (s, 1H), 7.38 (s, 1H), 7.15 (d, J = 2.1 Hz, 1H), 7.05 (dd, J = 8.1, 2.1 Hz, 1H), 6.82 (d, J = 8.1 Hz, 1H), 6.62 (d, J = 8.4 Hz, 2H), 6.34 (d, J = 8.4 Hz, 2H). ^{13}C NMR (150 MHz, DMSO-d_6) δ 155.4, 148.3, 144.6, 144.4, 142.0, 139.3, 131.2 (2×C), 127.7, 123.9, 122.6, 122.0, 118.0, 115.1, 114.7, 114.0 (2×C), 111.8. HRESIMS *m/z* 650.1654 $[M+NH_4]^+$ (calcd for $C_{36}H_{28}O_{11}N^+$, 650.1657).

1,4,6,9-Tetra(3,4-dihydroxyphenyl)dibenzo[*b*,*d*]furan-2,3,7,8-tetraol (**12**): a brown solid. m.p. >250 °C; IR (KBr) ν_{max} 3401, 1734, 1654, 1560, 1536, 1496, 1420, 1114, 1015 cm^{-1}. ^1H NMR (600 MHz, DMSO-d_6) δ 7.11 (d, J = 2.0 Hz, 1H), 7.00 (dd, J = 8.1, 2.0 Hz, 1H), 6.78 (d, J = 8.1 Hz, 1H), 6.31 (s, 1H), 6.30 (s, 1H), 6.11 (s, 1H). ^{13}C NMR (150 MHz, DMSO-d_6) δ 148.7, 145.1, 144.8, 144.3, 143.9, 142.3, 139.7, 128.4, 124.4, 123.1, 122.5, 121.9, 118.5, 117.9, 115.6, 115.0, 114.8, 112.1. HRESIMS *m/z* 663.1149 $[M-H]^-$ (calcd for $C_{36}H_{23}O_{13}^-$, 663.1144).

Synthesis of compounds 6 and 9

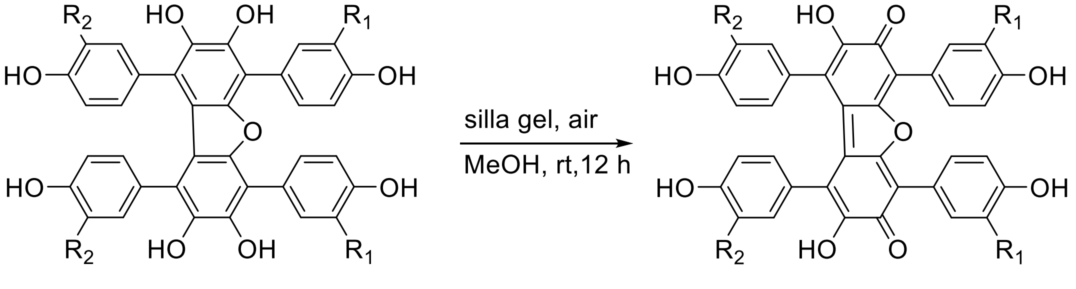

5: R_1=R_2=H
8: R_1=OH, R_2=H

6: R_1=R_2=H
9: R_1=OH, R_2=H

Compound **5** (10 mg, 0.0167 mmol) was dissolved in MeOH (4 mL), followed by the addition of 200−300 mesh

silica gel (1.3 g). The mixture reacted for 12 h at rt. The solvent was evaporated, and the residue was purified by flash column chromatography (FCC) eluting with EtOAc-CH_2Cl_2 (v/v 1:5) to give compound **6** (R_f = 0.2, 9.4 mg, 94% yield). Following the same procedures, compound **9** (R_f = 0.1, 4.6 mg, 92% yield) was synthesized from compound **8** (5 mg, 0.0079 mmol) with 200−300 mesh silica gel (0.67 g), by flash column chromatography (FCC), eluting with EtOAc-CH_2Cl_2 (v/v 1:5).

2,8-Dihydroxy-1,4,6,9-tetra(4-hydroxyphenyl)dibenzo[*b*,*d*]furan-3,7-dione (**6**): a blood solid. m.p. >250 °C; IR (KBr) ν_{max} 3370, 2924, 1676, 1588, 1514, 1438, 1348, 1285, 1177, 1014 cm^{-1}.^{1}H NMR (600 MHz, DMSO-d_6) δ 9.75 (s, 1H), 9.59 (s, 1H), 9.22 (s, 1H), 7.50 (d, J = 8.4 Hz, 2H), 6.84 (d, J = 8.5 Hz, 2H), 6.65 (d, J = 8.4 Hz, 2H), 6.37 (d, J = 8.5 Hz, 2H). ^{13}C NMR (150 MHz, DMSO-d_6) δ 180.6, 161.7, 158.0, 156.4, 147.4, 132.9, 132.5 (2×C), 130.8 (2×C), 125.1, 120.9, 115.9, 115.3 (2×C), 114.3 (2×C), 114.1. HRESIMS *m/z* 597.1195 $[M–H]^-$ (calcd for $C_{36}H_{21}O_9^-$, 597.1191).

2,8-Dihydroxy-4,6-bis(3,4-dihydroxyphenyl)-1,9-bis(4-hydroxyphenyl)dibenzo[*b*,*d*]furan-3,7-dione (**9**): blood solid. m.p. >250 °C; IR (KBr) ν_{max} 3401, 1686, 1654, 1604, 1513, 1437, 1307, 1174, 1117, 1015 cm^{-1}. ^{1}H NMR (600 MHz, DMSO-d_6) δ 9.53 (s, 1H), 9.21 (s, 1H), 9.20 (s, 1H), 9.03 (s, 1H), 7.10 (s, 1H), 7.00 (d, J = 8.1 Hz, 1H), 6.81 (d, J = 8.2 Hz, 1H), 6.65 (d, J = 8.1 Hz, 2H), 6.37 (d, J = 8.2 Hz, 2H). ^{13}C NMR (150 MHz, DMSO-d_6) δ 180.2, 161.2, 156.0, 146.9, 145.8, 144.7 (2×C), 132.4, 130.3, 124.7, 122.6, 120.8, 118.0, 115.3, 115.2, 113.8, 113.8 (2×C). HRESIMS *m/z* 629.1100 $[M–H]^-$ (calcd for $C_{36}H_{21}O_{11}^-$, 629.1089).

Synthesis of compound 10

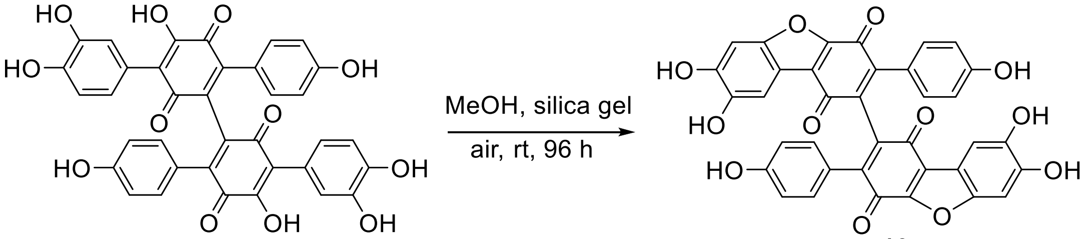

7 10

Compound **7** (9.7 mg, 0.015 mmol) was dissolved dissolved in MeOH (4 mL), followed by 200−300 mesh silica gel (1.3 g). The mixture was stirred for 96 h at rt, and the solvent was evaporated. The residue was purified by flash column chromatography (FCC), eluting with EtOAc-CH_2Cl_2 (v/v 1:5) to give compound **10** (R_f = 0.1, 4.2 mg, 44% yield).

2,2-Bis(7,8-dihydroxy-3-(4-hydroxyphenyl)dibenzo[*b*,*d*]furan-1,4-dione)(**10**): dark-red solid. m.p. >250 °C; IR (KBr) ν_{max} 3401, 1735, 1654, 1608, 1509, 1473, 1273, 1174, 1092, 1020 cm^{-1}. ^{1}H NMR (400 MHz, DMSO-d_6) δ 10.26 (s, 1H), 9.95 (s, 1H), 9.82 (s, 1H), 7.35 (s, 1H), 7.20 (s, 1H), 6.61 (d, J = 8.6 Hz, 2H), 6.45 (d, J = 8.6 Hz, 2H). ^{13}C NMR (150 MHz, DMSO-d_6) δ 183.2, 174.3, 158.4, 151.6, 150.2, 149.8, 146.9, 143.5, 138.2, 131.1(2×C), 121.8, 121.8, 114.6 (2×C), 113.4, 105.2, 98.7. HRESIMS *m/z* 641.0735 $[M–H]^-$ (calcd for $C_{36}H_{17}O_{12}^-$, 641.0725).

Synthesis of compound 15

Compound **1b** (10 mg, 0.032 mmol) was dissolved in MeOH (4 mL), followed by the addition of 200−300 mesh silica gel (1.3 g). The mixture was stirred for 72 h at 35 °C. After the solvent was evaporated, the residue was purified by flash column chromatography (FCC), eluting with EtOAc-CH_2Cl_2 (v/v 1:5) to give compound **15** (R_f = 0.3, 7.6 mg, 70% yield).

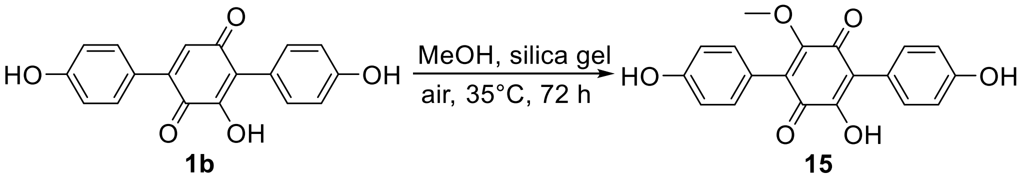

2,4',4"-Trihydroxy-5-methoxy-*p*-terphenyl-3,6-dione (**15**): dark-red solid. ^{1}H NMR (600 MHz, DMSO-d_6) δ 9.65 (s, 1H), 9.54 (s, 1H), 7.20 (d, J = 8.6 Hz, 2H), 7.14 (d, J = 8.6 Hz, 2H), 6.80 (d, J = 8.6 Hz, 2H), 6.78 (d, J = 8.6 Hz, 2H), 3.77 (s, 3H). ^{13}C NMR (150 MHz, DMSO-d_6) δ 184.1, 183.2, 157.9, 157.3, 155.6, 152.2, 132.4 (2×C), 132.2 (2×C), 125.7, 121.7, 121.0, 118.0, 115.1 (2×C), 114.8 (2×C), 61.7; LC-MS m/z 339.10 $[M+H]^+$.

Synthesis of Compounds 18 and 22

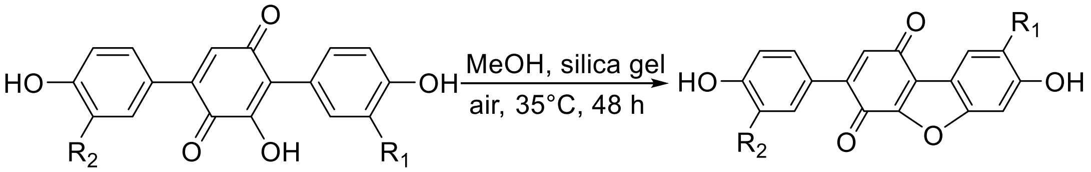

2b: R1= OH, R2= H
3b: R1=R2= OH
18: R1= OH, R2= H
22: R1=R2= OH

Compound **2b** (18 mg, 0.056 mmol) was dissolved in MeOH (6 mL), followed by the addition of 200−300 mesh silica gel (2.4 g). The mixture reacted for 48 h at 35 °C. After the solvent was evaporated, the residue was purified by flash column chromatography (FCC), eluting with EtOAc-CH_2Cl_2 (v/v 1:5) to give compound **18** (R_f = 0.2, 13.2 mg, 73% yield). Following the same procedures, compound **22** (R_f = 0.1, 8.6 mg, 48% yield) was synthesized from compound **3b** (18 mg, 0.053 mmol) and purified by flash column chromatography (FCC), eluting with EtOAc-CH_2Cl_2 (v/v 1:5).

7,8-Dihydroxy-3-(4-hydroxyphenyl)dibenzo[*b*,*d*]furan-1,4-dione (**18**): dark-red solid. m.p. >250 °C; IR (KBr) ν_{max} 3466, 2924, 1644, 1607, 1546, 1514, 1437, 1265, 1176, 1088, 1017 cm^{-1}. ^{1}H NMR (600 MHz, DMSO-d_6) δ 10.13 (s, 1H), 10.00 (s, 1H), 9.79 (s, 1H), 7.46 (d, J = 8.6 Hz, 2H), 7.31 (s, 1H), 7.15 (s, 1H), 6.85 (d, J = 8.6 Hz, 2H), 6.71 (s, 1H). ^{13}C NMR (150 MHz, DMSO-d_6) δ 184.3, 175.8, 159.8, 151.7, 150.9, 150.1, 146.9, 145.1, 131.8 (2×C), 130.9, 123.8, 122.2, 115.7 (2×C), 113.8, 105.6, 99.0. HRESIMS m/z 321.0406 $[M–H]^-$ (calcd for $C_{18}H_9O_6^-$, 321.0405).

7,8-Dihydroxy-3-(3,4-dihydroxyphenyl)dibenzo[*b*,*d*]furan-1,4-dione (**22**): dark-red solid. m.p. >250 °C; IR (KBr) ν_{max} 3369, 2921, 1655, 1554, 1522, 1437, 1384, 1265, 1202, 1126, 1018 cm^{-1}. ^{1}H NMR (600 MHz, DMSO-d_6) δ 7.31 (s, 1H), 7.15 (s, 1H), 7.04 (s, 1H), 6.94 (d, J = 7.9 Hz, 1H), 6.82 (d, J = 7.9 Hz 1H), 6.63 (s, 1H). ^{13}C NMR (150 MHz, DMSO-d_6) δ 183.9, 175.4, 151.3, 150.6, 149.7, 147.8, 146.5, 145.0, 144.8, 130.4, 123.8, 121.8, 121.6, 117.1, 115.6, 113.3, 105.3, 98.6; HRESIMS m/z 337.0354 $[M–H]^-$ (calcd for $C_{18}H_9O_7^-$,

337.0354).

Synthesis of Compound 19

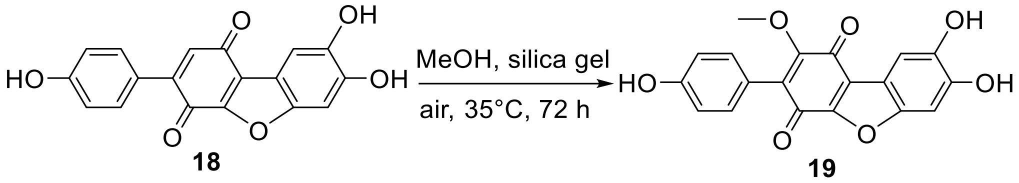

Compound **18** (10 mg, 0.031 mmol) was dissolved in MeOH (4 mL), followed by the addition of 200−300 mesh silica gel (1.3 g). The mixture was stirred for 72 h at 35 °C. After the solvent was evaporated, the residue was purified by flash column chromatography (FCC), eluting with EtOAc-CH_2Cl_2 (v/v 1:10) to give compound **19** (R_f = 0.3, 7.6 mg, 70% yield).

7,8-Dihydroxy-2-methoxy-3-(4-hydroxyphenyl)dibenzo[*b*,*d*]furan-1,4-dione (**19**): dark-red solid. m.p. >250 °C; IR (KBr) ν_{max} 3401, 2922, 1654, 1610, 1561, 1512, 1437, 1274, 1166, 1087, 1021 cm^{-1}. ^1H NMR (600 MHz, DMSO-d_6) δ 10.03 (s, 1H), 9.75 (s, 1H), 9.68 (s, 1H), 7.32 (s, 1H), 7.14 (s, 1H), 7.13 (d, J = 8.6 Hz, 2H), 6.81 (d, J = 8.6 Hz, 2H), 3.78 (s, 3H). ^{13}C NMR (150 MHz, DMSO-d_6) δ 179.7, 176.2, 157.6, 155.1, 151.0, 150.2, 149.1, 146.3, 132.0 (2×C), 128.9, 120.6, 120.3, 114.6 (2×C), 113.4, 105.2, 98.7, 61.2. HRESIMS *m/z* 351.0505 $[M–H]^-$ (calcd for $C_{19}H_{11}O_7^-$, 351.0510).

Synthesis of Compounds 13, 16 and 20

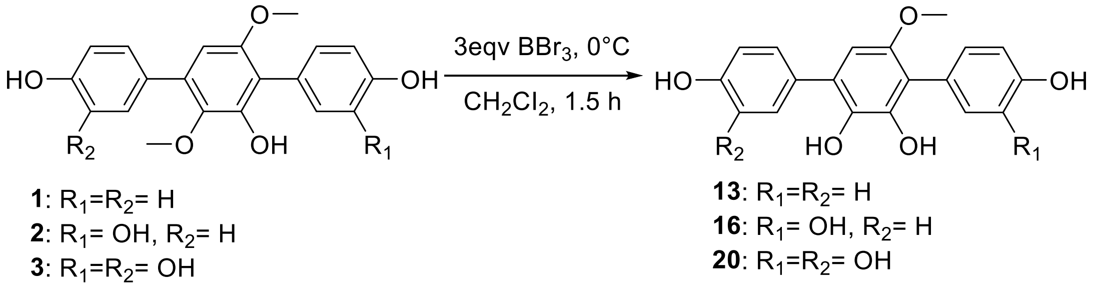

Compound **1** (30 mg, 0.089 mmol) was dissolved in CH_2Cl_2 (5 mL), followed by the addition of BBr_3 (0.4 mL, 0.27 mmol). The mixture was stirred for 1.5 h at 0 °C. Then EtOAc (200 mL) was added, and the organic layer was washed three times with H_2O (20 mL each). The organic layer was dried over anhydrous Na_2SO_4 and concentrated in vacuo. The residue was purified by semipreparative HPLC on an ODS-A column (YMC-pack ODS-A, 10×250 mm), eluting with 50% MeOH/H_2O supplemented with 0.15% TFA at a flow rate of 4.0 mL/min, to provide **13** (t_R = 11.3 min, 24.2 mg, 84% yield). Following the same procedures, compound **16** (t_R = 9.1 min, 25.2 mg, 87% yield) was synthesized from compound **2** (30 mg, 0.085 mmol) and purified by HPLC, eluting with 40% MeOH/H_2O supplemented with 0.15% TFA at a flow rate of 4.0 mL/min. Similarly, compound **20** (t_R = 9.2 min, 24.5 mg, 85% yield) was synthesized from compound **3** (30 mg, 0.081 mmol) and purified by HPLC, eluting with 30% MeOH/H_2O supplemented with 0.15% TFA at a flow rate of 4.0 mL/min.

6-Methoxy-*p*-terphenyl-2,3, 4',4''-tetraol (**13**): a yellow solid. ^1H NMR (600 MHz, DMSO-d_6) δ 7.42 (d, J = 8.4 Hz, 2H), 7.09 (d, J = 8.5 Hz, 2H), 6.81 (d, J = 8.4 Hz, 2H), 6.76 (d, J = 8.5 Hz, 2H), 6.36 (s, 1H), 3.59 (s, 3H).

^{13}C NMR (150 MHz, DMSO-d_6) δ 156.3, 155.9, 150.5, 145.0, 136.3, 131.9 (2×C), 130.2 (2×C), 129.7, 127.9, 124.8, 117.2, 114.8 (2×C), 114.4 (2×C), 103.3, 55.7; LC-MS *m/z* 325.15 $[M+H]^+$.

6-Methoxy-*p*-terphenyl-2,3,3',4',4''-pentaol (**16**): a yellow solid. m.p. >250 °C; IR (KBr) ν_{max} 3211, 1677, 1611, 1522, 1489, 1436, 1270, 1174, 1103, 1017, 952 cm^{-1}. ^1H NMR (600 MHz, DMSO-d_6) δ 9.41 (s, 1H), 8.75 (s, 1H), 8.74 (s, 1H), 8.00 (s, 1H), 7.77 (s, 1H), 7.42 (d, J = 8.5 Hz, 2H), 6.81 (d, J = 8.5 Hz, 2H), 6.72 (d, J = 8.0 Hz, 1H), 6.68 (d, J = 1.9 Hz, 1H), 6.54 (dd, J = 8.0, 1.9 Hz, 1H), 6.34 (s, 1H), 3.58 (s, 3H). ^{13}C NMR (150 MHz, DMSO-d_6) δ 156.3, 150.4, 144.8, 144.4, 144.0, 136.2, 130.1 (2×C), 129.6, 127.6, 125.2, 121.9, 118.5, 117.5, 114.9, 114.9 (2×C), 103.3, 55.7. HRESIMS *m/z* 339.0872 $[M–H]^-$ (calcd for $C_{19}H_{15}O_6^-$, 339.0874).

6-Methoxy-*p*-terphenyl-2,3,3',4',3'',4''-hexaol (**20**): a yellow solid. m.p. >250 °C; IR (KBr) ν_{max} 3181, 1600, 1530, 1468, 1435, 1314, 1243, 1204, 1130, 1019, 952 cm^{-1}. ^1H NMR (600 MHz, DMSO-d_6) δ 7.04 (d, J = 2.2 Hz, 1H), 6.86 (dd, J = 8.1, 2.2 Hz, 1H), 6.77 (d, J = 8.1 Hz, 1H), 6.72 (d, J = 8.0 Hz, 1H), 6.68 (d, J = 2.0 Hz, 1H), 6.54 (dd, J = 8.0, 2.0 Hz, 1H), 6.31 (s, 1H), 3.58 (s, 3H). ^{13}C NMR (150 MHz, DMSO-d_6) δ 150.3, 144.7, 144.7, 144.4, 144.4, 144.0, 136.2, 130.1, 127.7, 125.2, 121.9, 120.1, 118.5, 117.4, 116.7, 115.3, 114.9, 103.2, 55.7. HRESIMS *m/z* 357.0968 $[M+H]^+$ (calcd for $C_{19}H_{17}O_7^+$, 357.0969).

Synthesis of compound 14

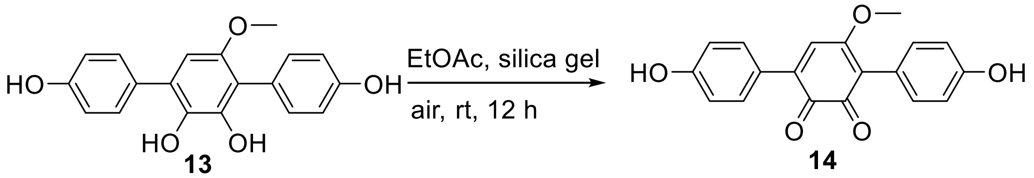

Compound **13** (20 mg, 0.062 mmol) was dissolved in EtOAc (8 mL), followed by the addition of 200−300 mesh silica gel (2.7 g). The mixture was stirred for 12 h at rt, and the solvent was evaporated. The residue was purified by flash column chromatography (FCC), eluting with EtOAc-CH_2Cl_2 (v/v 1:5) to give compound **14** (R_f = 0.2, 15.6 mg, 78% yield).

6-Methoxy-4',4''-dihydroxy-*p*-terphenyl-2,3-dione (**14**): wine solid. m.p. >250 °C; IR (KBr) ν_{max} 3191, 1686, 1610, 1514, 1438, 1408, 1267, 1231, 1176, 1113, 1021, 952 cm^{-1}. ^1H NMR (600 MHz, DMSO-d_6) δ 9.87 (s, 1H), 9.48 (s, 1H), 7.47 (d, J = 8.6 Hz, 2H), 7.42 (s, 1H), 7.07 (d, J = 8.6 Hz, 2H), 6.83 (d, J = 8.6 Hz, 2H), 6.74 (d, J = 8.6 Hz, 2H), 3.96 (s, 3H). ^{13}C NMR (150 MHz, DMSO-d_6) δ 179.8, 177.7, 163.8, 158.7, 156.5, 138.9, 131.8 (2×C), 130.4 (2×C), 127.1, 124.7, 122.0, 118.9, 115.2 (2×C), 114.5 (2×C), 57.5. HRESIMS *m/z* 321.0765 $[M–H]^-$ (calcd for $C_{19}H_{13}O_5^-$, 321.0757).

Synthesis of Compounds 17 and 21

Compound **16** (20 mg, 0.059 mmol) was dissolved in MeOH (7 mL), followed by the addition of 200−300 mesh silica gel (2.7 g). The mixture was stirred for 48 h at 35 °C, and the solvent was evaporated. The residue was purified by flash column chromatography (FCC), eluting with EtOAc-CH_2Cl_2 (v/v 1:5) to give compound **17** (R_f = 0.3, 16.4 mg, 82% yield). Following the same procedures, compound **21** (R_f = 0.1, 15.2 mg, 77% yield) was synthesized from compound **20** (20 mg, 0.056 mmol) and purified by flash column chromatography (FCC),

eluting with EtOAc-CH_2Cl_2 (v/v 1:10).

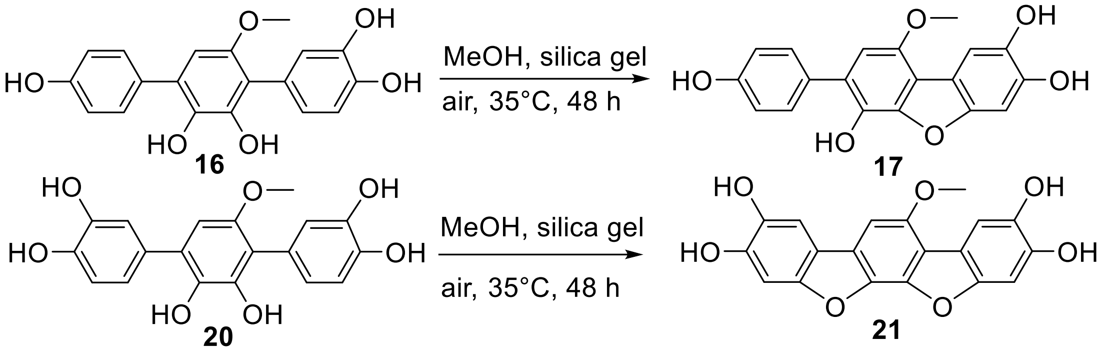

7-(4-Hydroxyphenyl)-9-methoxydibenzo[*b*,*d*]furan-2,3,6-triol (**17**): a dark-red solid. m.p. >250 °C; IR (KBr) ν_{max} 3401, 1654, 1611, 1468, 1437, 1316, 1267, 1171, 1099, 1024, 956 cm^{-1}. ^1H NMR (600 MHz, DMSO-d_6) δ 7.47 (d, J = 8.6 Hz, 2H), 7.38 (s, 1H), 7.04 (s, 1H), 6.83 (d, J = 8.6 Hz, 2H), 6.68 (s, 1H), 3.94 (s, 3H). ^{13}C NMR (150 MHz, DMSO-d_6) δ 156.3, 149.3, 146.9, 146.3, 145.7, 142.4, 132.9, 130.6 (2×C), 129.4, 126.2, 114.9 (2×C), 114.3, 113.3, 107.2, 105.5, 98.4, 55.8. HRESIMS *m/z* 337.0716 $[M–H]^-$ (calcd for $C_{19}H_{13}O_6^-$, 337.0707).

6-Methoxy-2,6':3,6''-diepoxy-*p*-terphenyl-3',4',3'',4''-tetraol (**21**): a dark red solid. m.p. >250 °C; IR (KBr) ν_{max} 3204, 1610, 1472, 1235, 1314, 1083, 1131, 1055, 1016, 952 cm^{-1}. ^1H NMR (600 MHz, DMSO-d_6) δ 7.47 (s, 1H), 7.43 (s, 1H), 7.39 (s, 1H), 7.12 (s, 1H), 7.10 (s, 1H), 4.08 (s, 3H). ^{13}C NMR (150 MHz, DMSO-d_6) δ 150.6, 150.3, 149.2, 146.3, 145.6, 142.8, 142.8, 139.7, 134.5, 123.4, 115.6, 114.4, 112.6, 107.1, 105.6, 98.7, 98.6, 95.2, 56.2. HRESIMS *m/z* 351.0507 $[M–H]^-$ (calcd for $C_{19}H_{11}O_7^-$, 351.0499).

Bioactivity assay procedures

Oxygen radical absorbance capacity (ORAC) assay.

The anti-oxidative activity of compounds **4**–**22** was assessed using the ORAC assay.[S2] This assay was primarily conducted with 2,2'-azobis(2-amidinopropane)dihydrochloride (AAPH) at a concentration of 153.0 μM, fluorescein (FL) at 81.6 nM, the test compounds, and trolox as a positive control, all dissolved in a phosphate buffer solution (PBS) at 75 mM with a pH of 7.4. The concentrations for compounds **4**–**22** and Trolox were set at 6.25 μM. In each well, we added 25 μL of the test compounds, blank (PBS), negative control (PBS), and trolox, followed by 150 μL of fluorescein, and incubated the mixture at 37 °C for 10 minutes. Subsequently, 25 μL of AAPH was added to the wells containing the test compounds, blank, and trolox, while 25 μL of PBS was added to the negative control group. The fluorescence intensity of each well was measured every minute for 90 cycles using a Fluoroskan Ascent FL plate reader (Thermo Scientific Varioskan LUX), with an excitation wavelength of 485 nm and an emission wavelength of 530 nm. The relative fluorescence intensity f was calculated as the ratio of the absolute fluorescence reading to the initial fluorescence reading. The net area under the curve (AUC) was determined by subtracting the AUC of the blank from that of the compound. The AUC was calculated as $0.5 + f_1 + ... f_i + ... + f_{89} + 0.5 \times f_{90}$, where fi represents the ratio of fluorescence reading at time i to the initial fluorescence reading. The final ORAC values were reported as micromoles of trolox equivalents per micromole of the compound (μM TE/μM). This calculation was based on a regression equation relating the

trolox concentration to the net area under the fluorescein decay curve, specifically:

$$\text{Relative ORAC value} = (AUC_{compound} - AUC_{blank}) / (AUC_{trolox} - AUC_{blank}).$$

DPPH Radical-scavenging Assay

The anti-oxidative activity of compounds **4–22** was assessed using the 2,2-diphenyl-1-picrylhydrazyl (DPPH) radical-scavenging assay.[S3] The experiment included four groups: blank (methanol, MeOH), sample (a mixture of the compound and DPPH solution), background (pure compound solution), negative (pure DPPH solution), and positive (a mixture of vitamin C and DPPH solution) controls. DPPH was prepared at a concentration of 0.15 mM, while compounds **4–22** and VC were tested at concentrations ranging from 1 to 100 μM, with all substances dissolved in methanol. For the blank group, 200 μL of MeOH was added. In contrast, 160 μL of the compound or vitamin C solution was added to the sample and background or positive groups. Additionally, 40 μL of MeOH was added to the background group, while 40 μL of DPPH solution was added to the negative, positive, and sample groups. After incubating the mixtures in the dark at rt for 30 minutes, the decrease in DPPH radical concentration was measured by assessing the absorbance (A) at a wavelength of 517 nm using a microplate reader (Multiscan Spectrum, Thermo Scientific Varioskan LUX). The DPPH radical-scavenging rate (%) was calculated as $[(A_{negative} - A_{blank}) - (A_{sample} - A_{background})] / (A_{negative} - A_{blank}) \times 100\%$. The IC_{50} values (half maximal inhibitory concentration) for the compounds and VC were determined using SPSS (Statistical Package for the Social Sciences) software, based on the radical-scavenging rates measured at final concentrations of 100, 50, 10, 5, and 1 μM.

α-Glucosidase Inhibitory Assay

The inhibitory activities of compounds **4–22** against α-glucosidase derived from *Saccharomyces cerevisiae* were evaluated using a previously reported method.[S4] The testing compounds were initially dissolved in dimethyl sulfoxide (DMSO) to create a stock solution at a concentration of 10 mM, which was then diluted in phosphate buffer solution (PBS, pH 6.8). The α-glucosidase enzyme (2.0 U/mL, Sigma), *p*-nitrophenyl-α-D-glucopyranoside (pNPG, 2.5 mM, Macklin), sodium carbonate (Na_2CO_3, 0.2 M), and acarbose (2.5 mg/mL, Sigma) were directly dissolved in PBS for the assay. In a 96-well microplate, 20 μL of the compound solutions and acarbose were mixed with 20 μL of α-glucosidase and 60 μL of PBS, serving as the drug and positive control groups, respectively. Pure PBS solution was utilized as the blank group. Following a 15-minute incubation at 37 °C, 20 μL of pNPG solution was added to each well in the testing groups, and the mixtures were further incubated at 37 °C for an additional 30 minutes. To terminate the reaction, 80 μL of Na_2CO_3 solution was subsequently added to each well. The absorbance was then measured using a microplate reader (Multiscan Spectrum, Thermo Scientific Varioskan LUX) at a wavelength of 405 nm. The inhibitory rate was calculated as $[1 - (A_{drug}/A_{blank})] \times 100\%$. The IC_{50} values for the compounds were determined using SPSS software, based on the inhibitory rates recorded at final concentrations of 500, 250, 50, 25, 5, 1, and 0.2 μM.

PTP1B Inhibitory Assay

The inhibitory activity of protein tyrosine phosphatase 1B (PTP1B) was assessed using *p*-nitrophenyl phosphate disodium salt (pNPP) as the substrate.[S5,S6] The assay buffer was composed of 10 mM Tris, 25 mM NaCl and 1 mM EDTA, adjusted to a pH of 7.1. The experimental procedure was carried out as follows: 2 μL of the sample dissolved in DMSO, was added to 48 μL of the buffer solution. Following this, 60 μL of 20 μg/mL PTP1B was introduced and mixed thoroughly in the well, and the mixture was incubated at 37 °C for 5 minutes. After the initial incubation, 40 μL of a 10 mM pNPP solution was added to the reaction mixture, which was then incubated at 37 °C for an additional 20 minutes. The reaction was subsequently halted by adding 50 μL of 2 M NaOH. The absorbance (A) of *p*-nitrophenol, which serves as a product of the reaction, was measured at a wavelength of 405 nm using a multiwell microplate reader. For controls, oleanolic acid was used as the positive control, while the negative control consisted of the buffer solution replacing PTP1B. Additionally, DMSO was used as the blank control. Each experiment was conducted at least three times to ensure consistency and reliability of the results. The inhibitory rate was calculated as $[1 - (A_{drug}/A_{blank})] \times 100\%$, The IC_{50} values for the compounds were determined using SPSS software, based on the inhibitory rates recorded at final concentrations of from 25 μg/mL to 0.04 μg/mL.

References

[S1] Xu, Y.; Wang, Y.; Wu, D.; He, W.; Wang, L.; Zhu, W. *p*-Terphenyls from *Aspergillus* sp. GZWMJZ-055: identification, derivation, antioxidant and α-glycosidase inhibitory activities. *Front. Microbiol.* **2021**, *12*, 654963.

[S2] Huang, D.; Ou, B.; Hampsch-woodill, M.; Flanagan, J. A.; Prior, R. L. High-throughput assay of oxygen radical absorbance capacity (ORAC) using a multichannel liquid handling system coupled with a microplate fluorescence reader in 96-well format. *J. Agric. Food. Chem.* **2002**, *50*, 4437−4444.

[S3] Wang, W. L.; Zhu, T. J.; Tao, H. W.; Lu, Z. Y.; Fang, Y. C.; Gu, Q. Q. Three novel, structurally unique spirocyclic alkaloids from the halotolerant B-17 fungal strain of *Aspergillus variecolor*. *Chem. Biodivers.* **2007**, *4*, 2913−2919.

[S4] Xu, Y.; Wang, C.; Liu, H.; Zhu, G.; Fu, P.; Wang, L.; Zhu, W. Meroterpenoids and isocoumarinoids from a *Myrothecium* fungus associated with *Apocynum venetum*. *Mar. Drugs* **2018**, *16*, 363−376.

[S5] Li, D.; Zhang, S.; Yang, C.; Li, Q.; Wang, S.; Xu, X.; Hao, J.; Li, C. A novel PTP1B inhibitor-phosphate of polymannuronic acid ame-liorates insulin resistance by regulating IRS-1/Akt signaling. *Int. J. Mol. Sci.* **2021**, *22*, 12693−12712.

[S6] Luo, J.; Hou, Y.; Xie, M.; Ma, W.; Shi, D.; Jiang, B. CYC31, A natural bromophenol PTP1B inhibitor, activates insulin signaling and improves long chain-fatty acid oxidation in C2C12 myotubes. *Mar. Drugs* **2020**, *18*, 267−279.

Figure S1. The LC-MS spectrum of compound **1a**

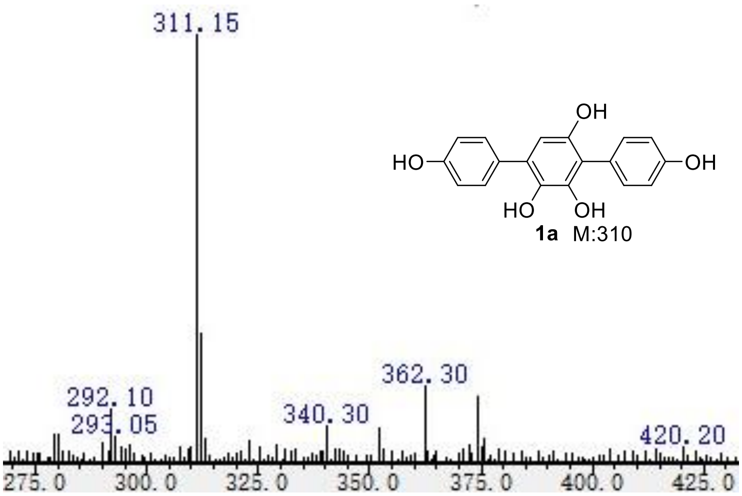

Figure S2. The LC-MS spectrum of compound **1b**

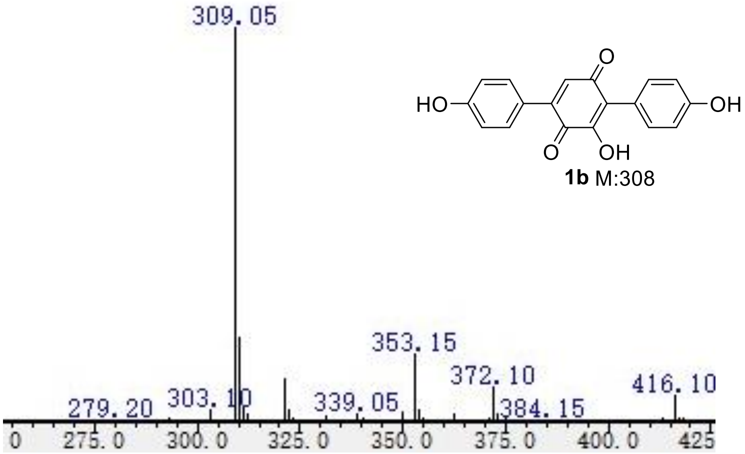

Figure S3. The LC-MS spectrum of compound **2a**

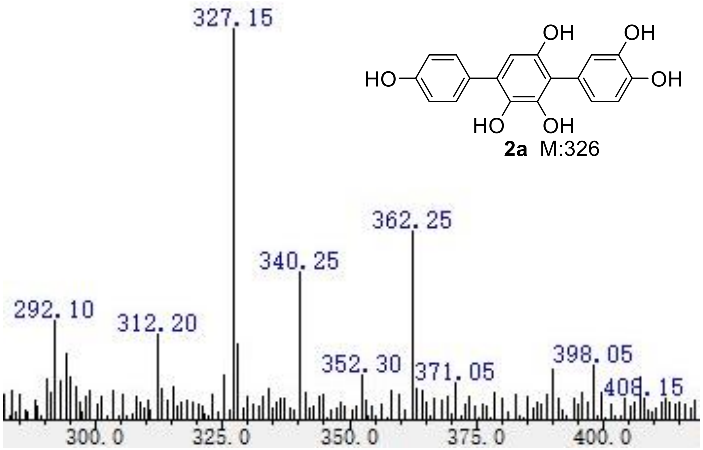

Figure S4. The LC-MS spectrum of compound **2b**

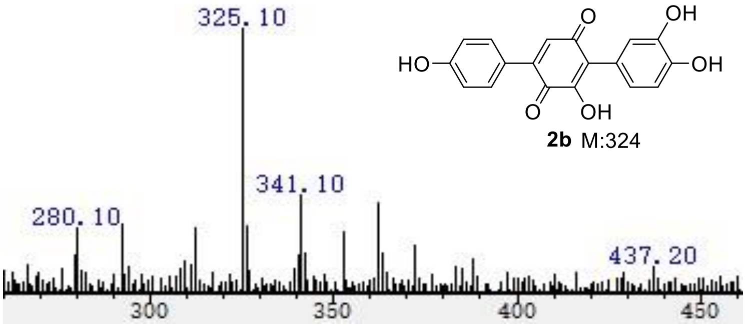

Figure S5. The LC-MS spectrum of compound **3a**

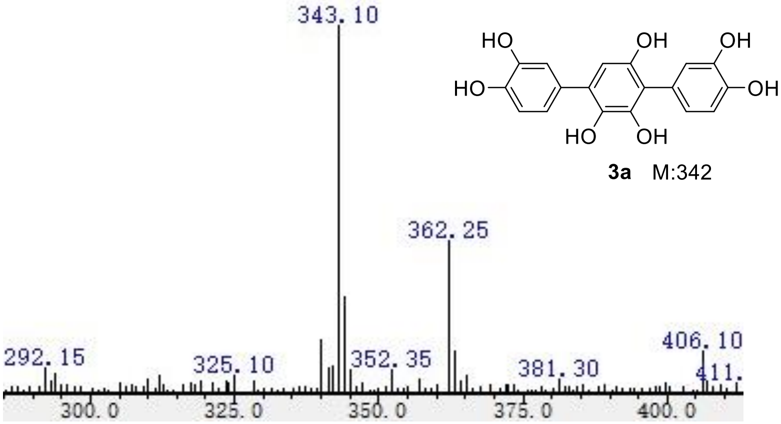

Figure S6. The LC-MS spectrum of compound **3b**

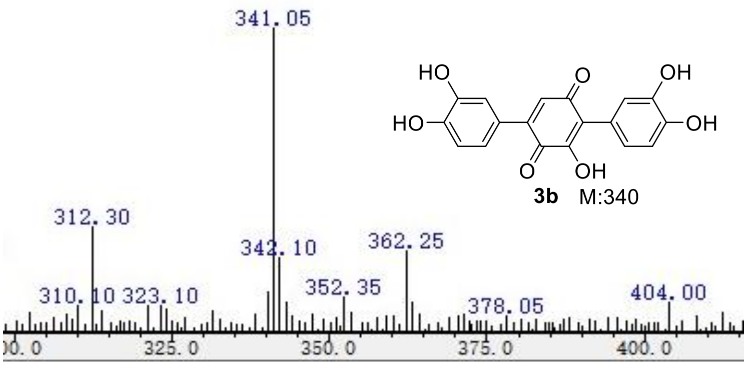

Figure S7. The ^{1}H (600 MHz) NMR spectrum of compound **4** in DMSO-d_6

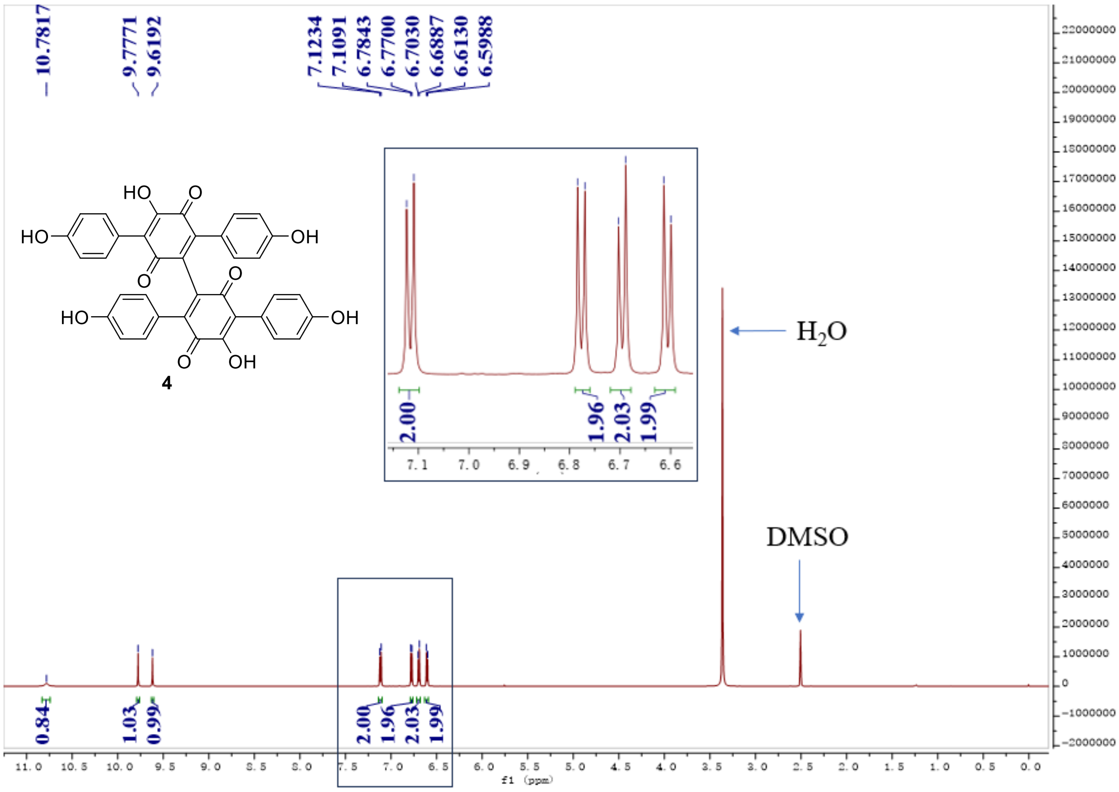

Figure S8. The ^{13}C (150 MHz) NMR spectrum of compound **4** in DMSO-d_6

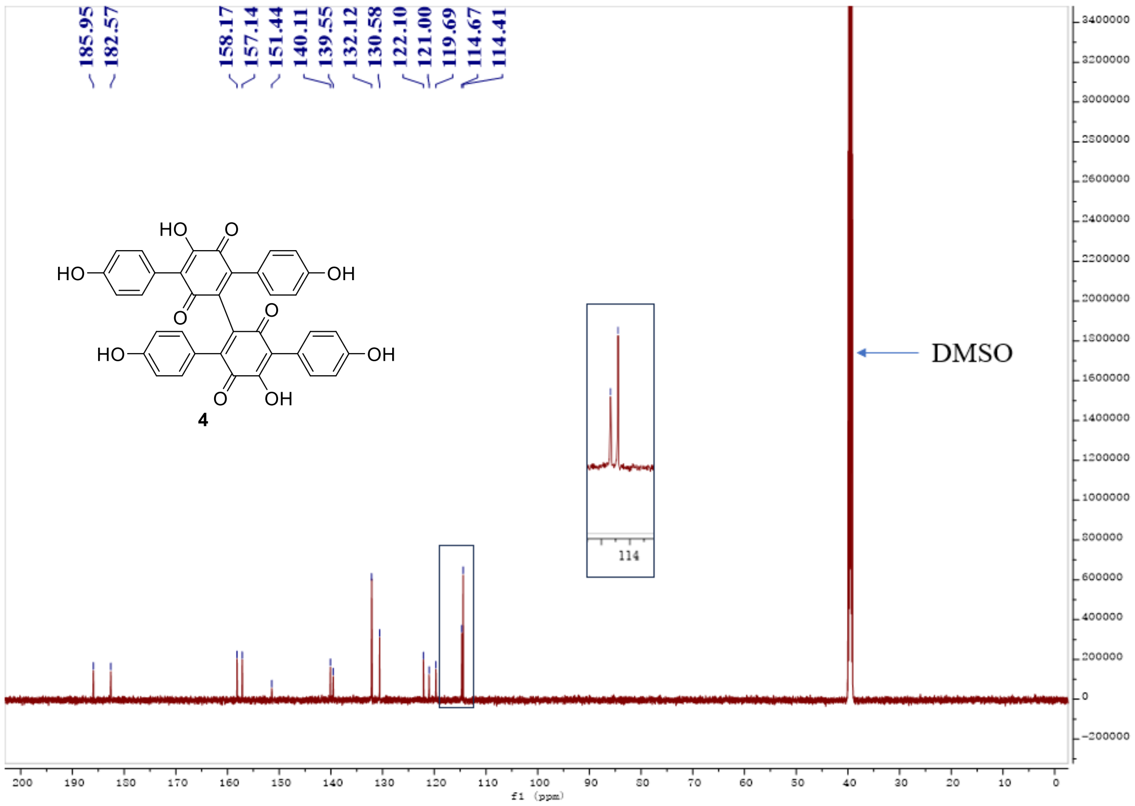

Figure S9. The HSQC spectrum of compound **4** in DMSO-d_6

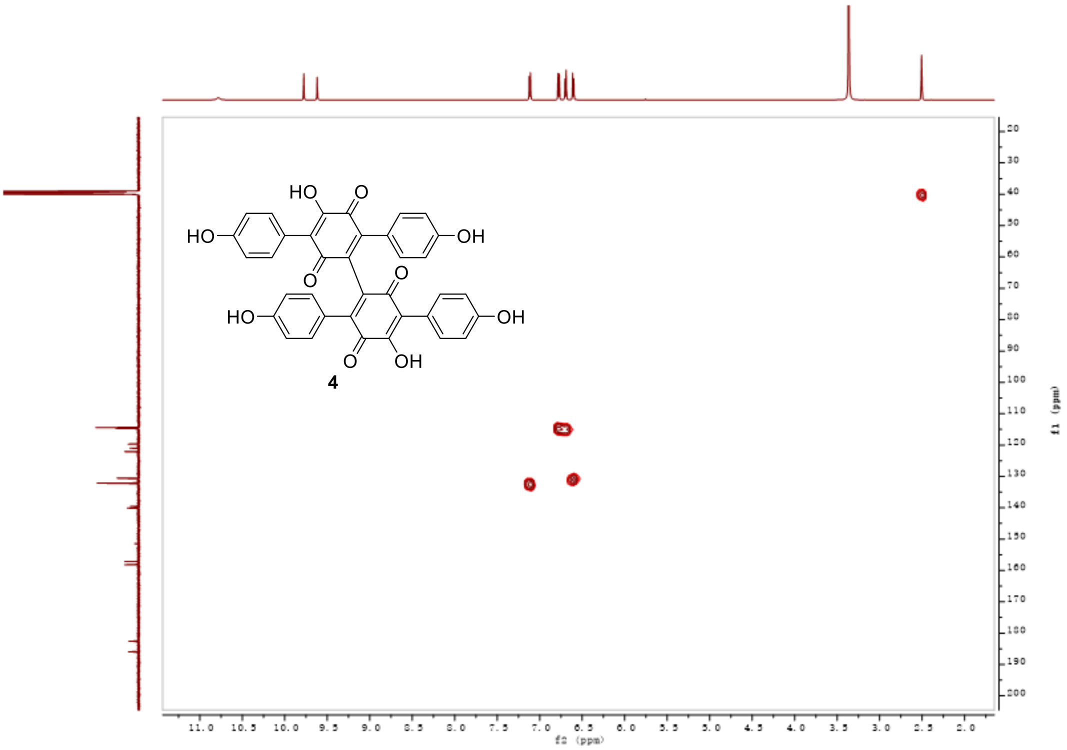

Figure S10. The ^{1}H-^{1}H COSY spectrum of compound **4** in DMSO-d_6

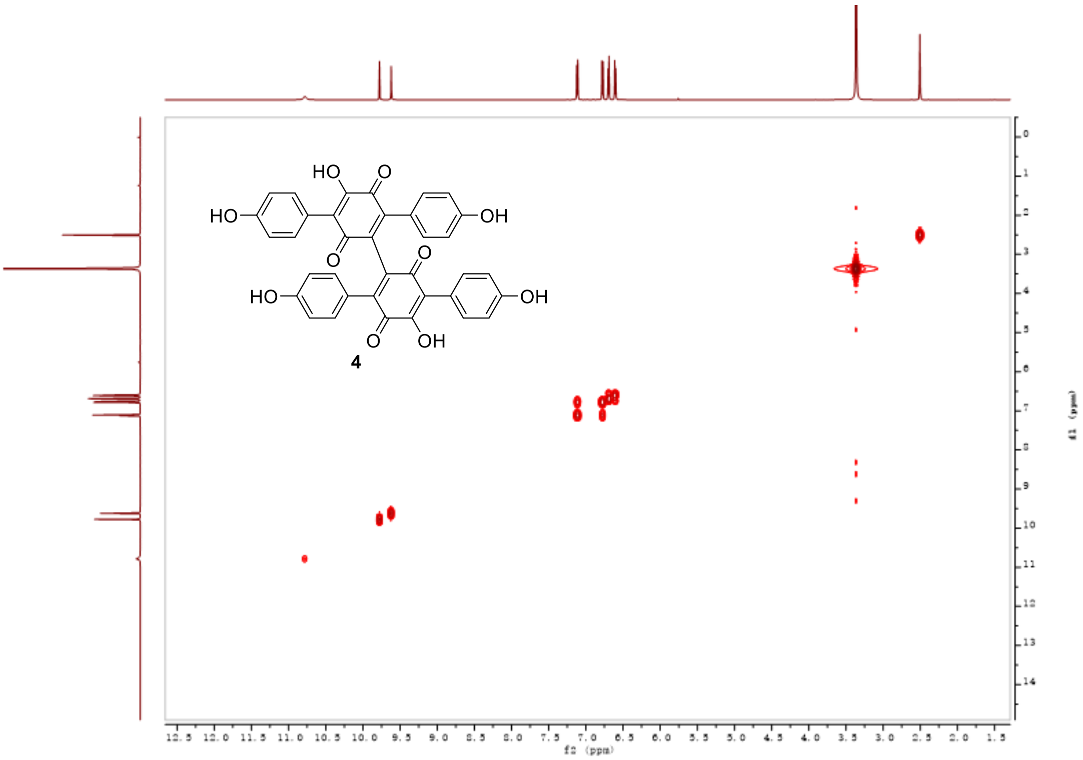

Figure S11. The HMBC spectrum of compound **4** in DMSO-d_6

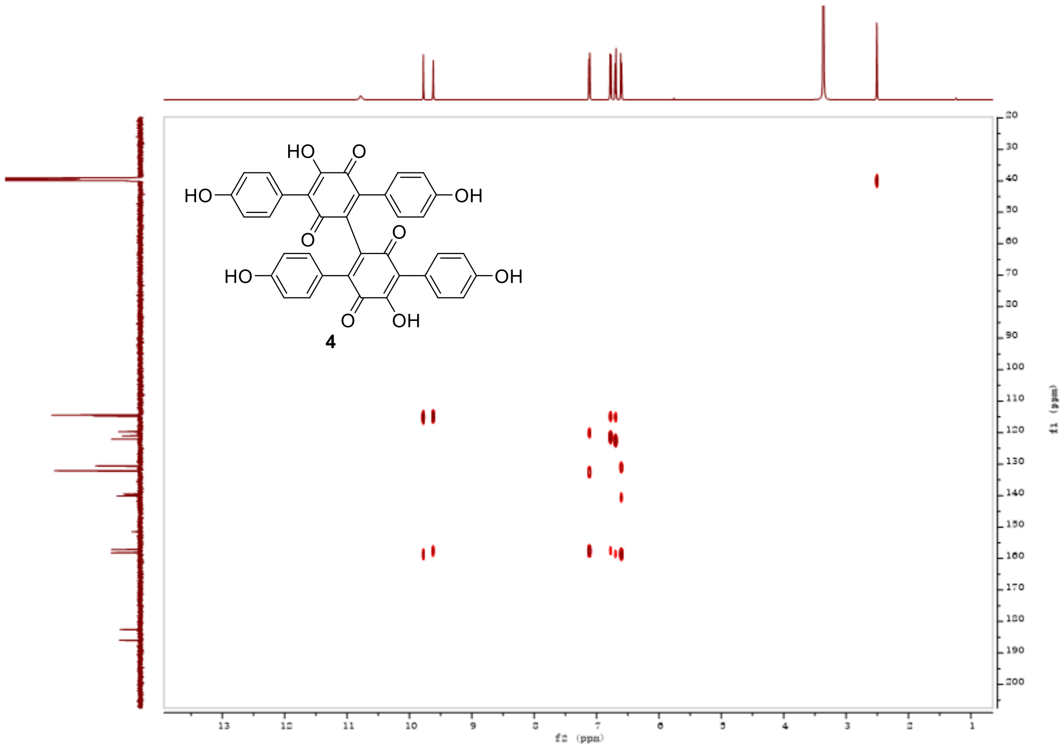

Figure S12. The HRESIMS spectrum of compound **4**

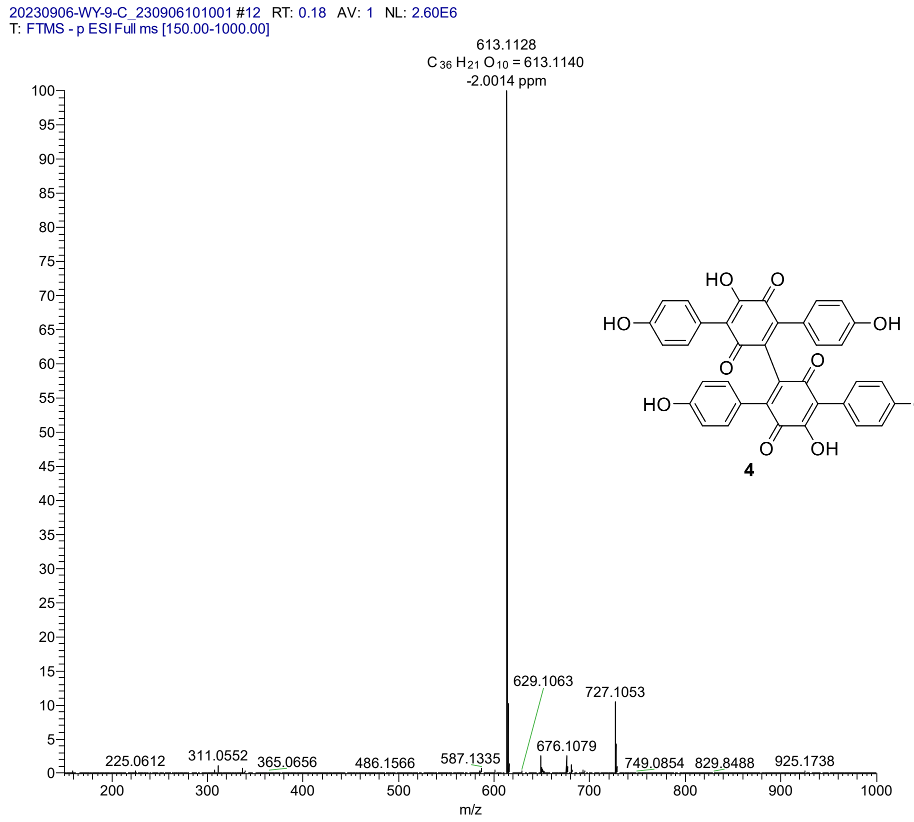

Figure S13. The ^{1}H (600 MHz) NMR spectrum of compound **5** in DMSO-d_6

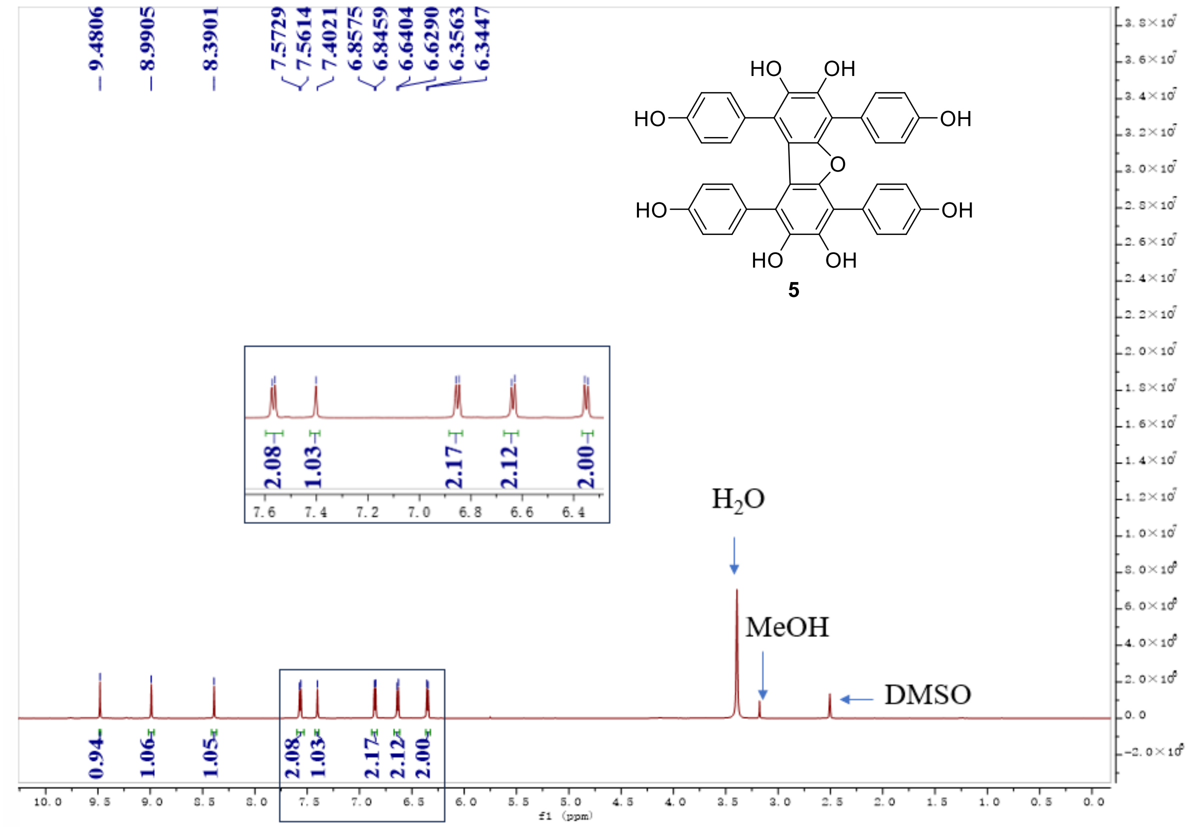

Figure S14. The ^{13}C (150 MHz) NMR spectrum of compound **5** in DMSO-d_6

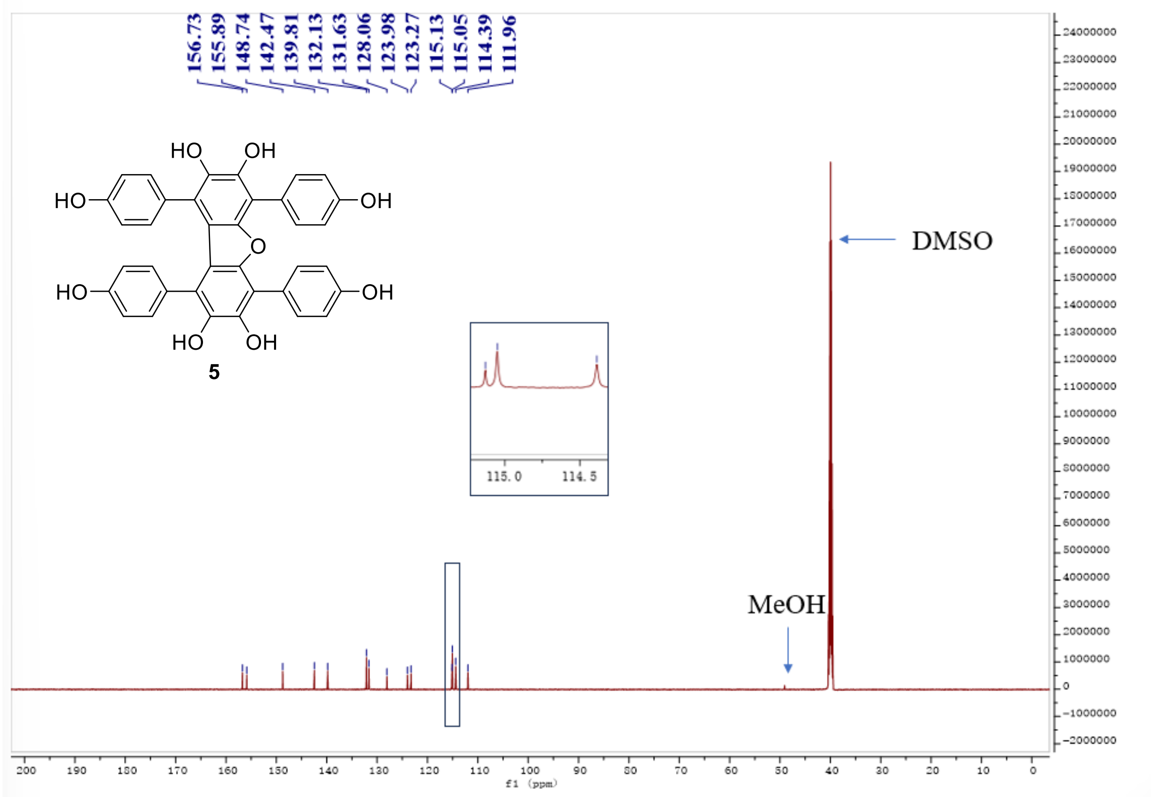

Figure S15. The HSQC spectrum of compound **5**

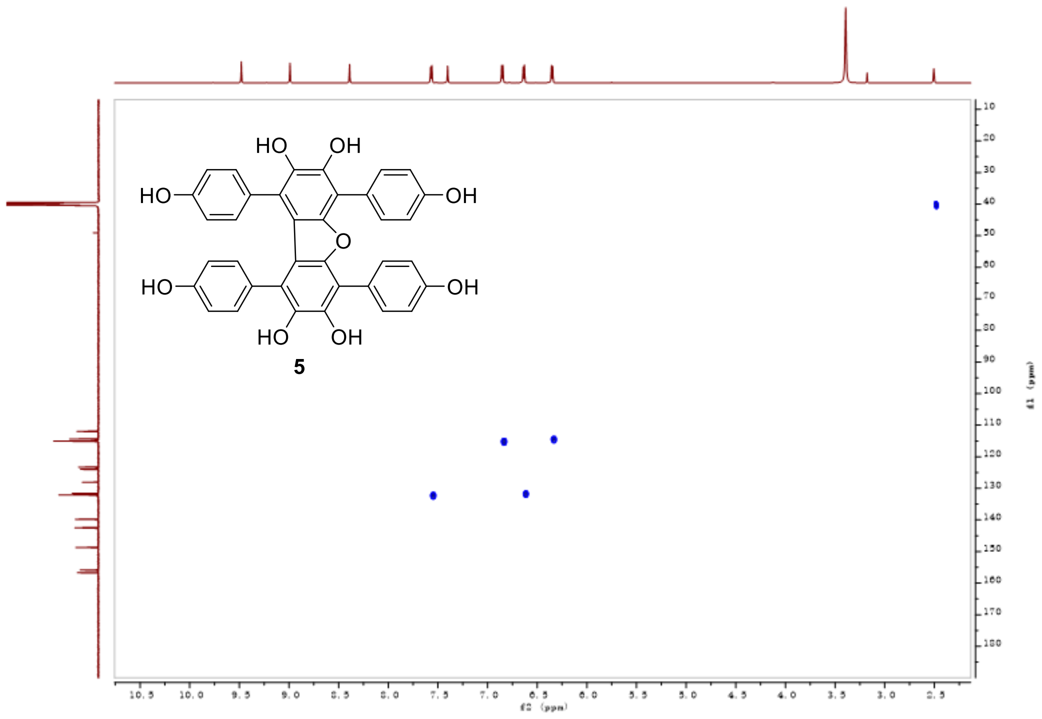

Figure S16. The ^{1}H-^{1}H COSY spectrum of compound **5**

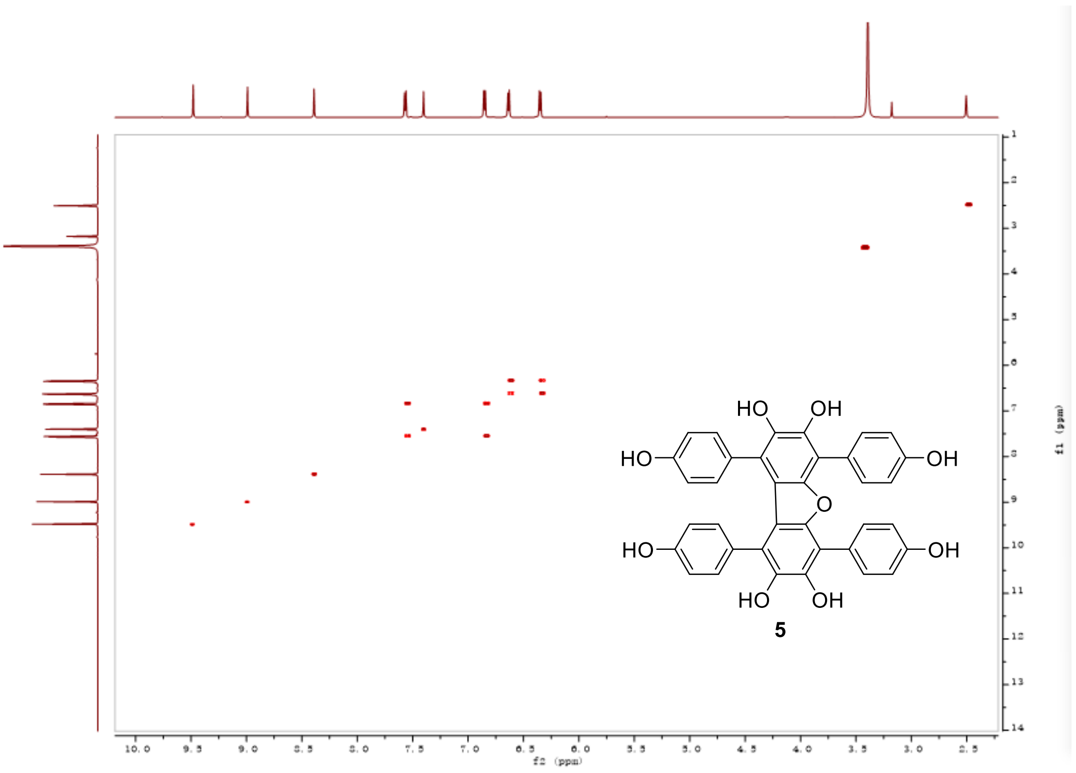

Figure S17. The HMBC spectrum of compound **5**

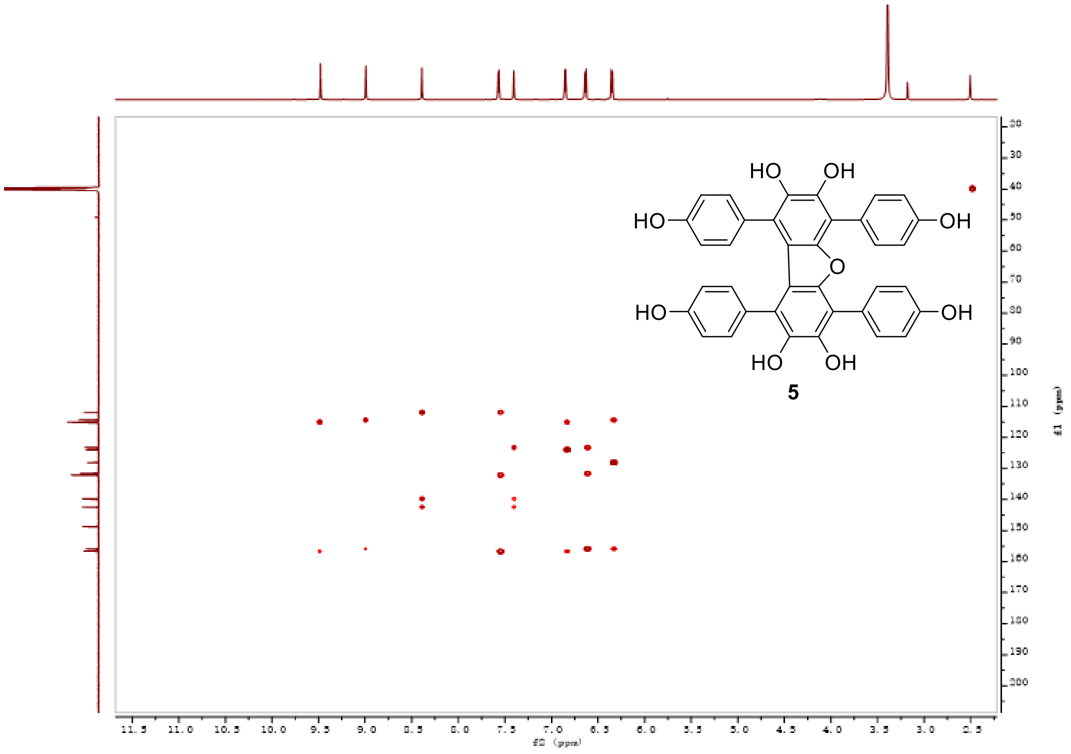

Figure S18. The HRESIMS spectrum of compound **5**

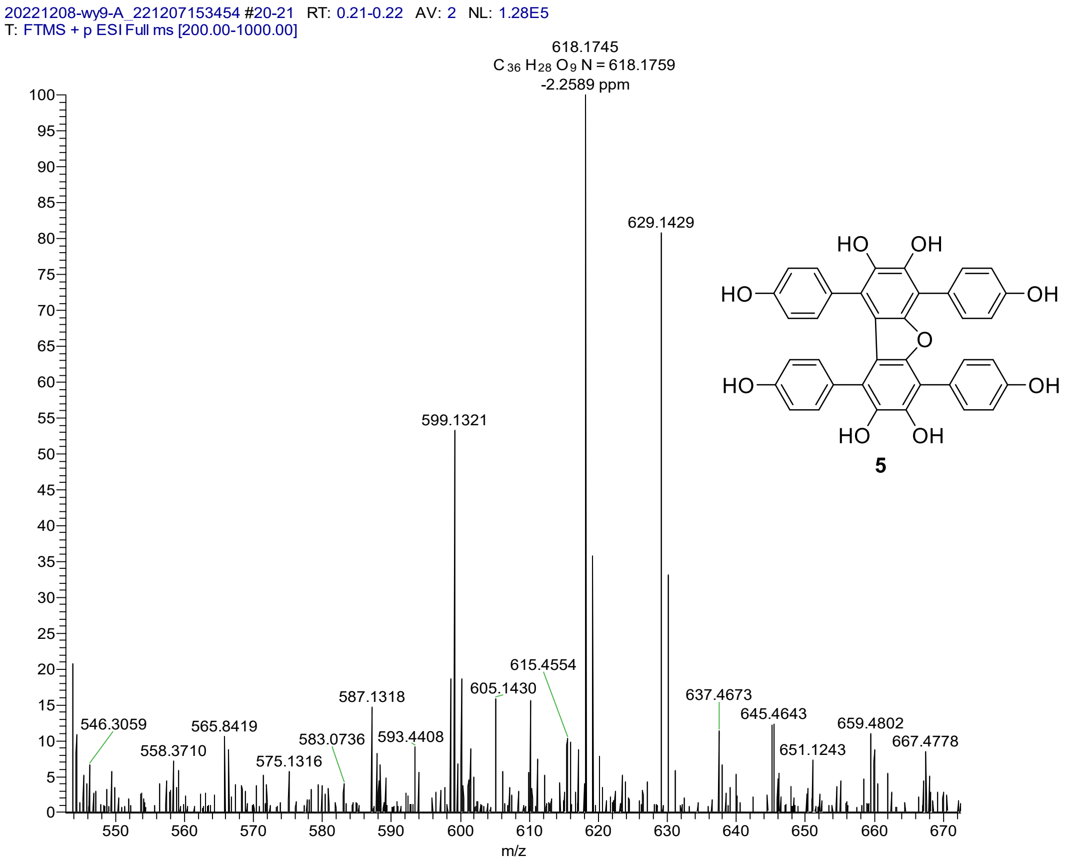

Figure S19. The ^{1}H (600 MHz) NMR spectrum of compound **6** in DMSO-d_6

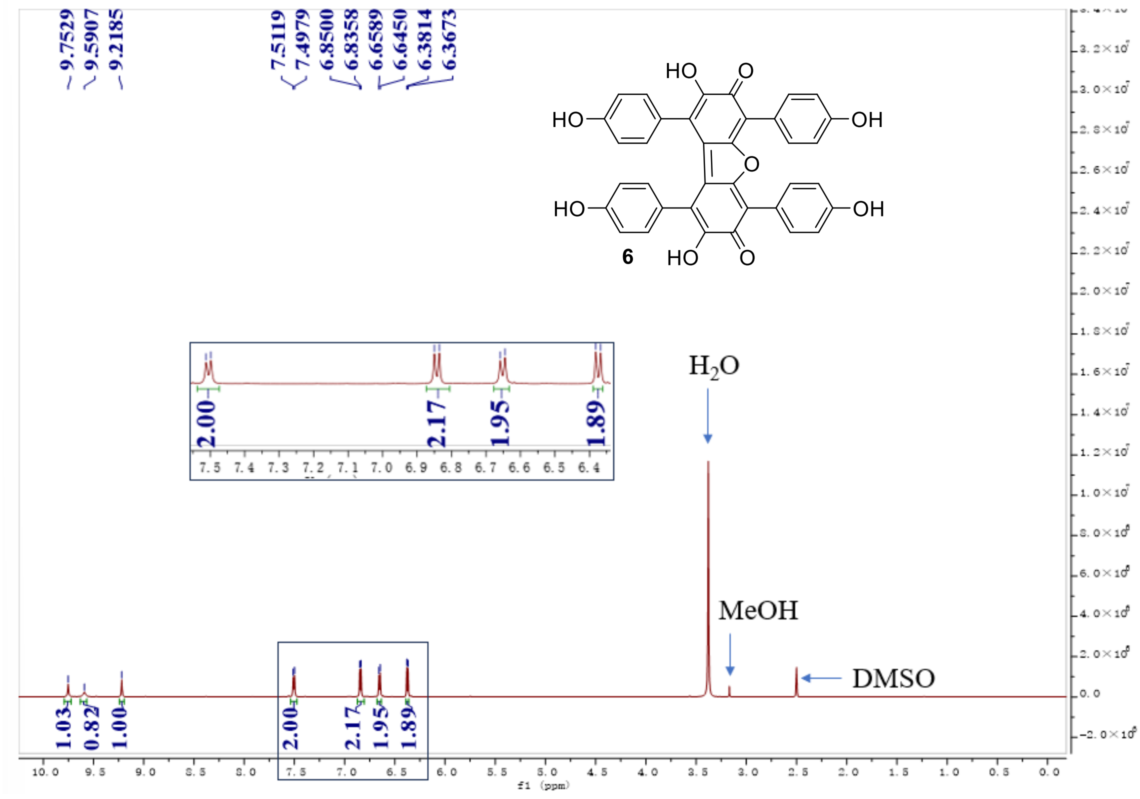

Figure S20. The ^{13}C (150 MHz) NMR spectrum of compound **6** in DMSO-d_6

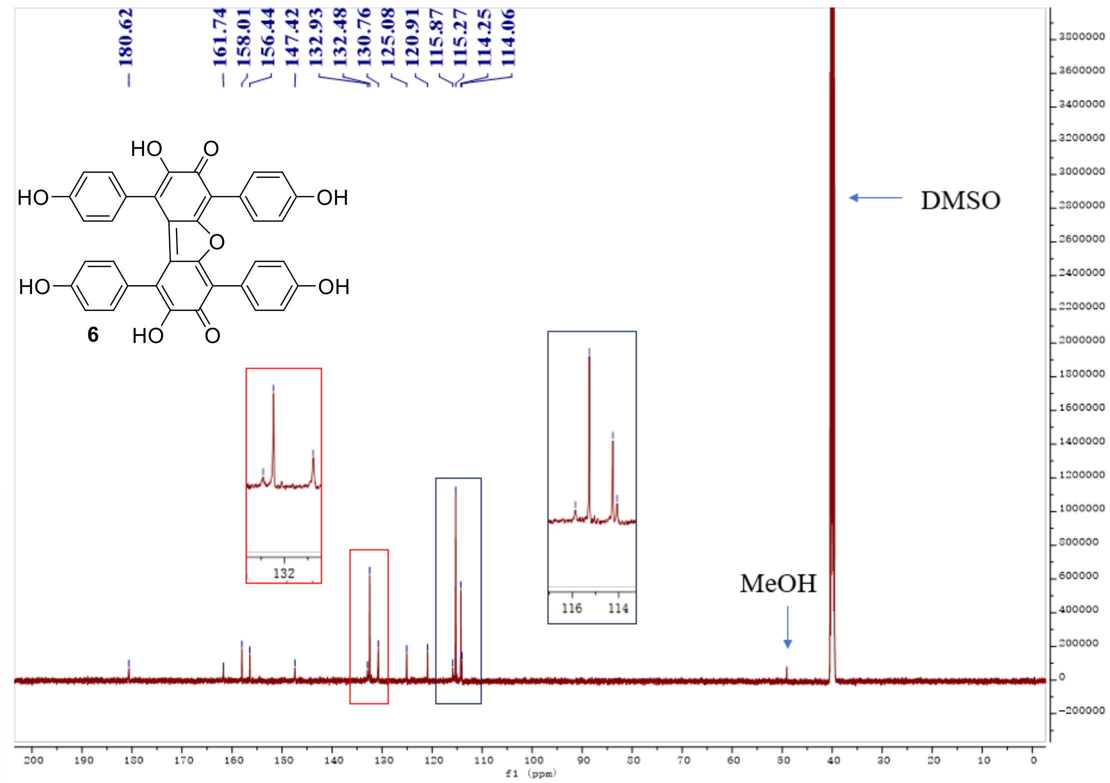

Figure S21. The HRESIMS spectrum of compound **6**

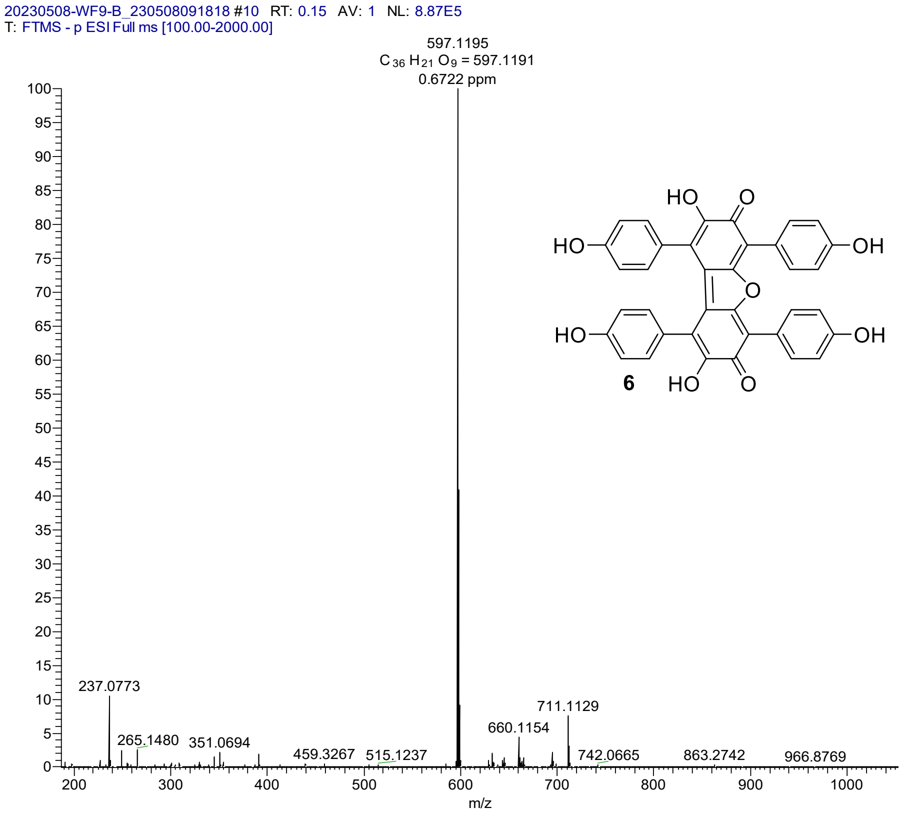

Figure S22. The ^{1}H (600 MHz) NMR spectrum of compound **7** in DMSO-d_6

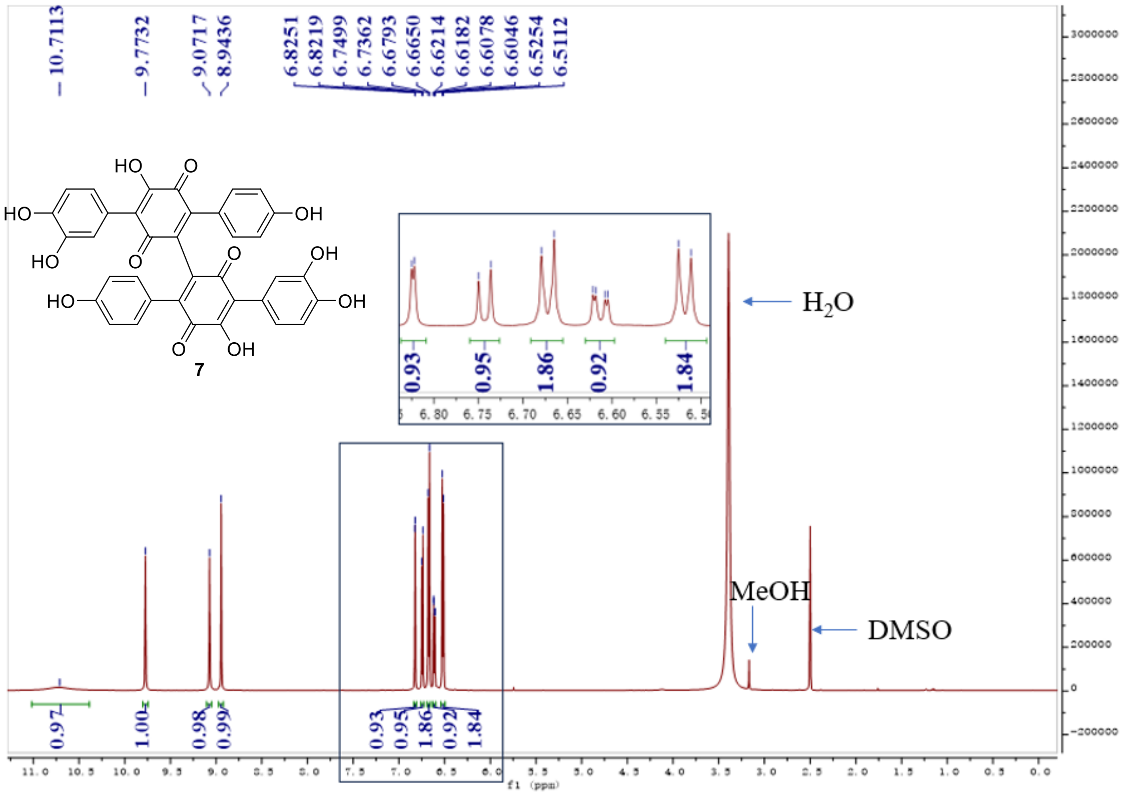

Figure S23. The ^{13}C (150 MHz) NMR spectrum of compound **7** in DMSO-d_6

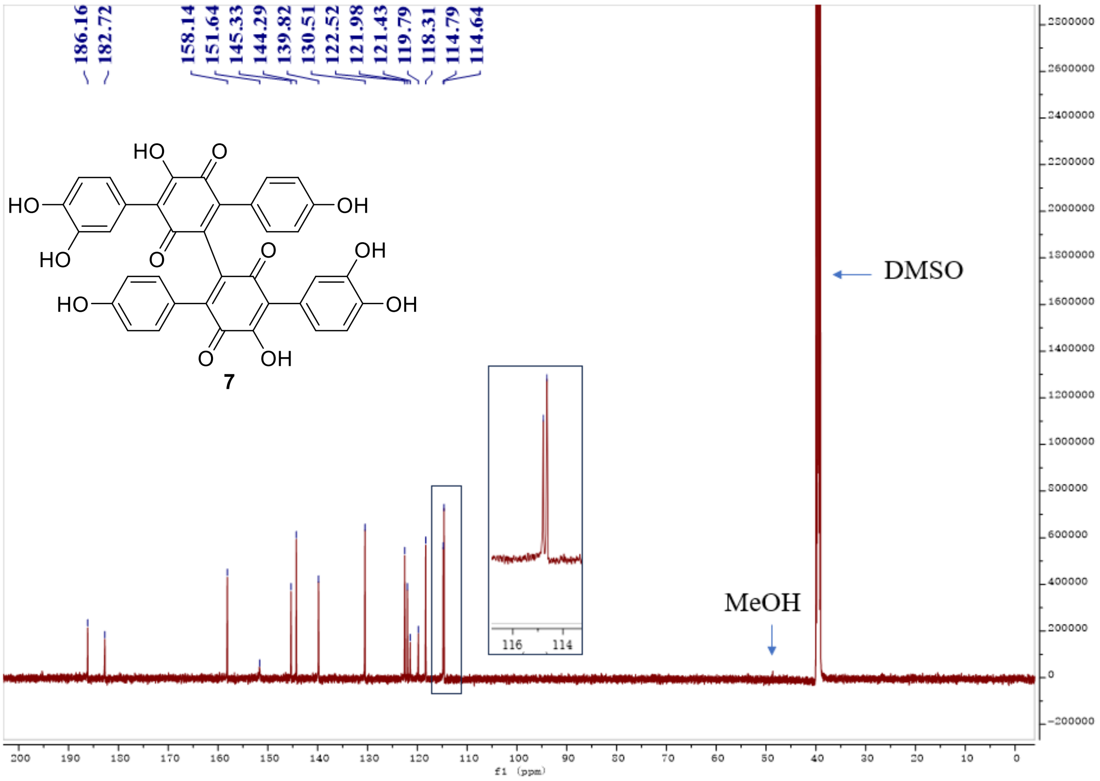

Figure S24. The HRESIMS spectrum of compound **7**

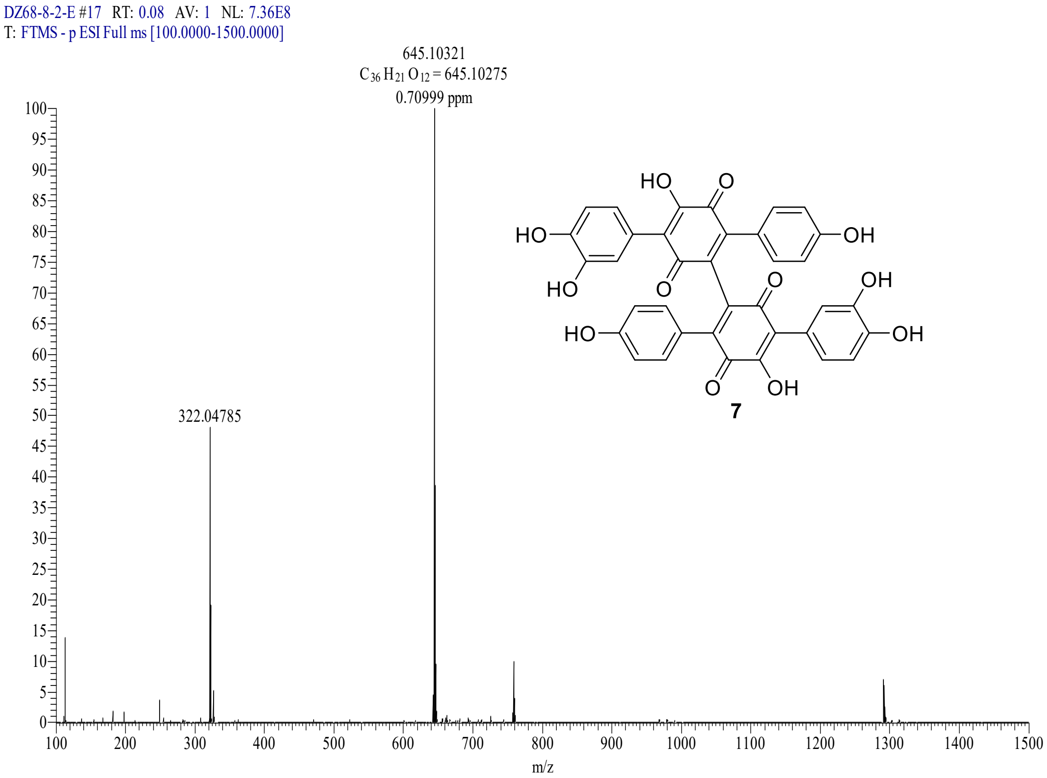

Figure S25. The ^{1}H (600 MHz) NMR spectrum of compound **8** in DMSO-d_6

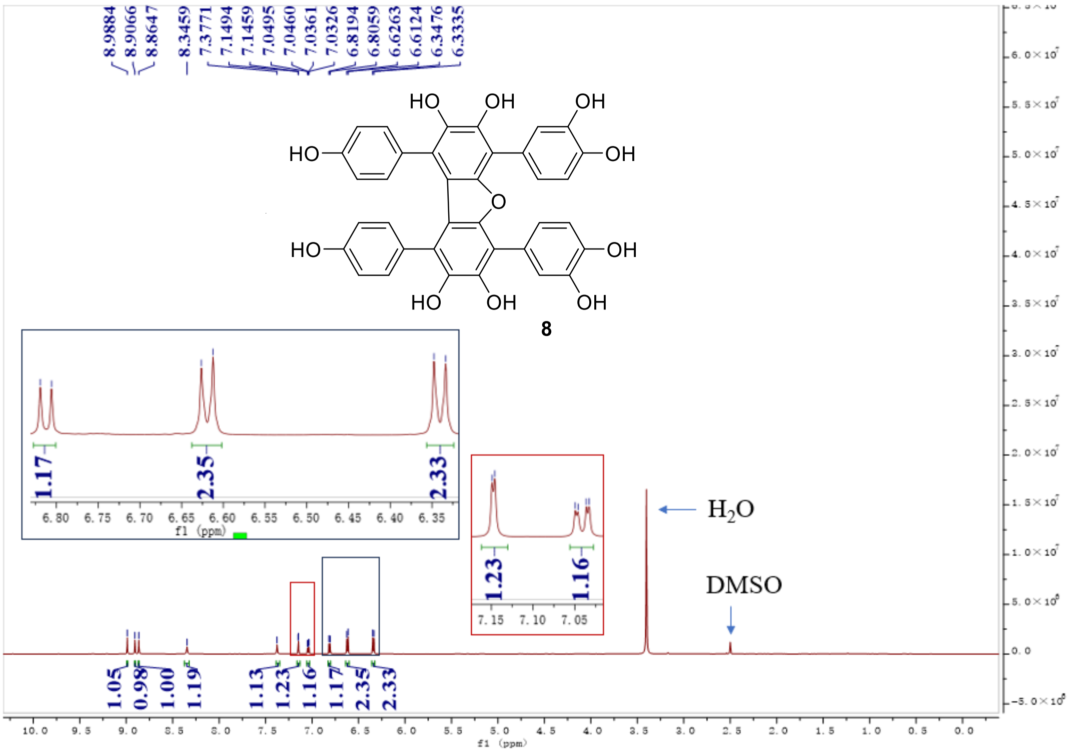

Figure S26. The ^{13}C (150 MHz) NMR spectrum of compound **8** in DMSO-d_6

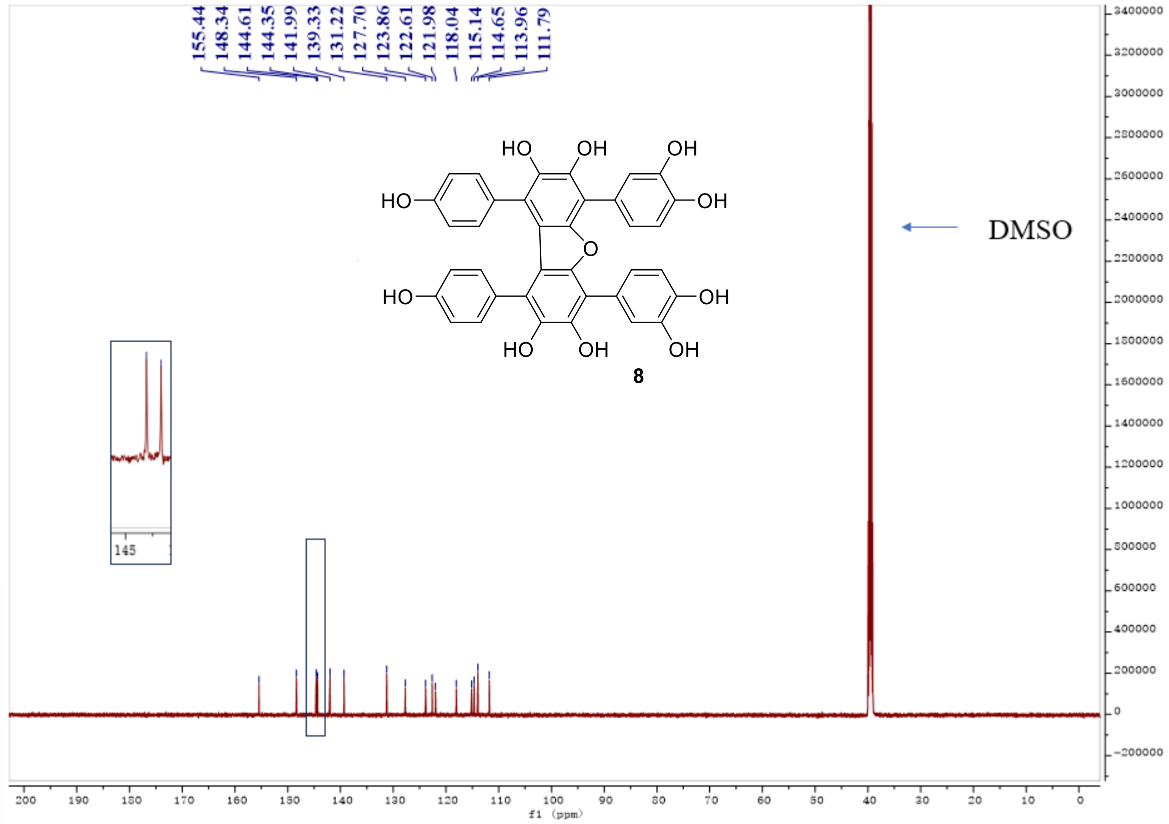

Figure S27. The HRESIMS spectrum of compound **8**

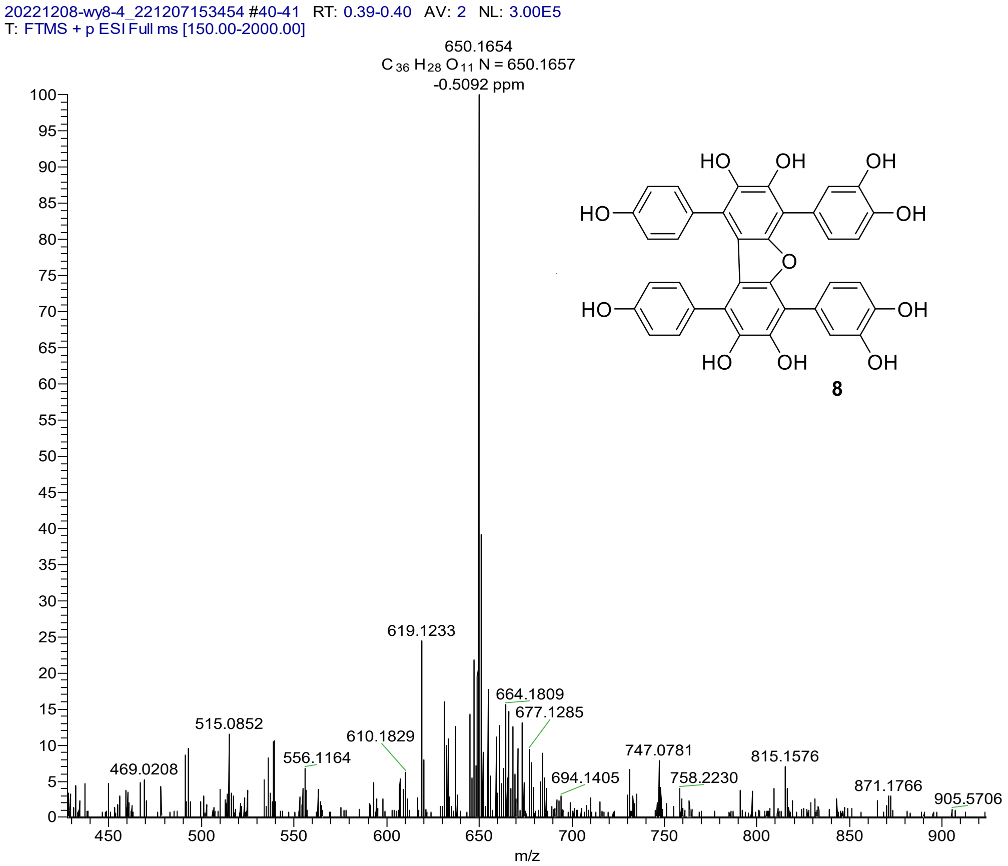

Figure S28. The ^{1}H (600 MHz) NMR spectrum of compound **9** in DMSO-d_6

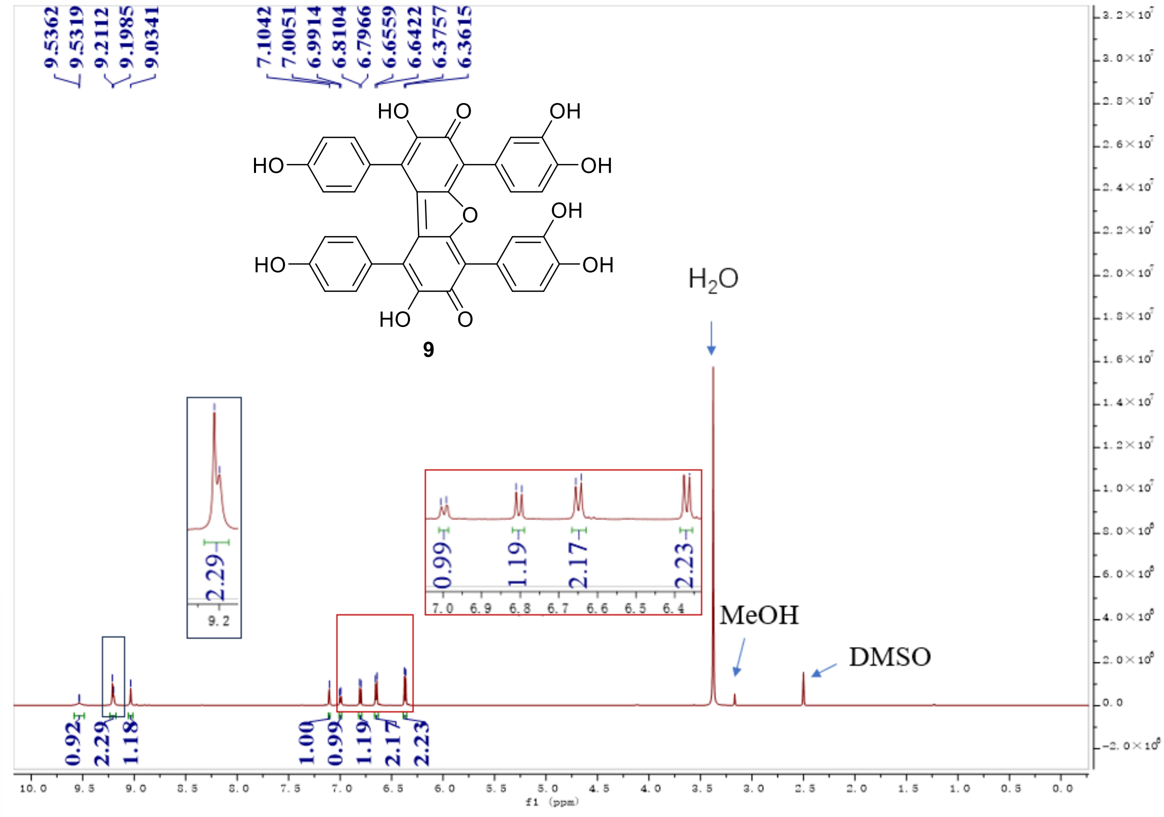

Figure S29 The ^{13}C (150 MHz) NMR spectrum of compound **9** in DMSO-d_6

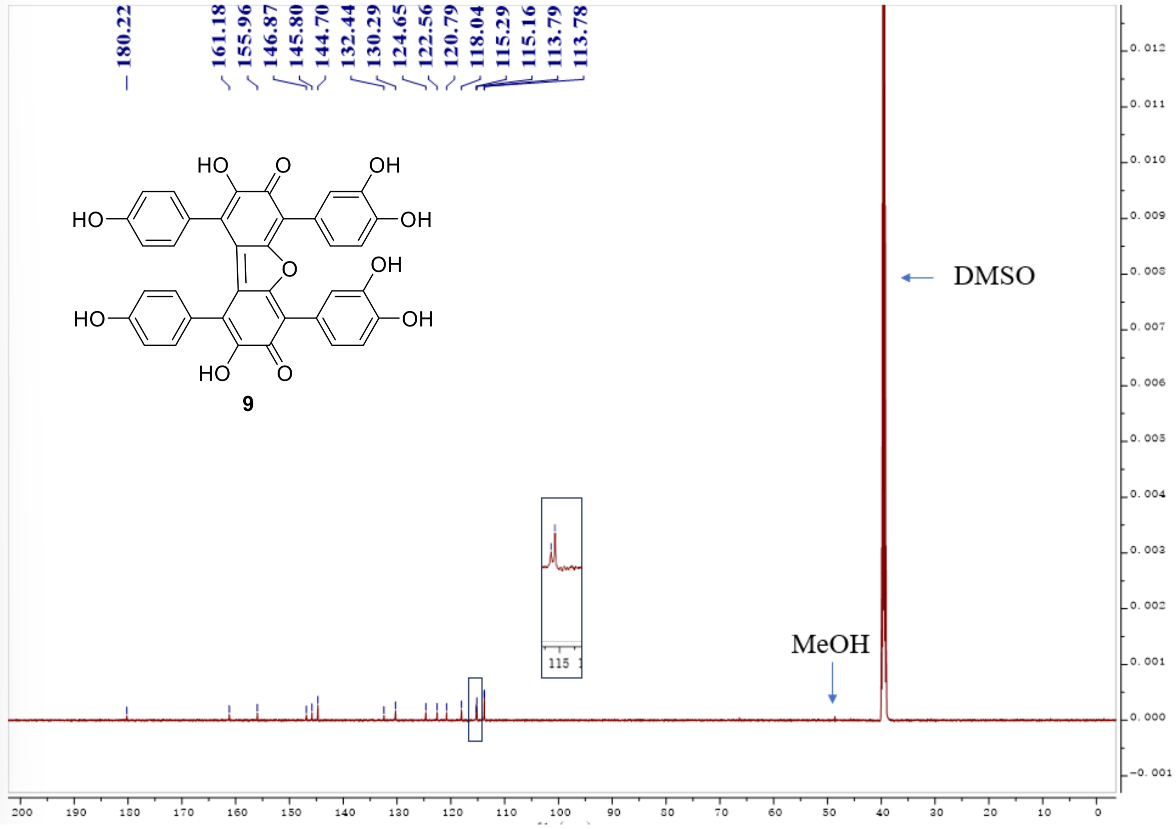

Figure S30. The HRESIMS spectrum of compound **9**

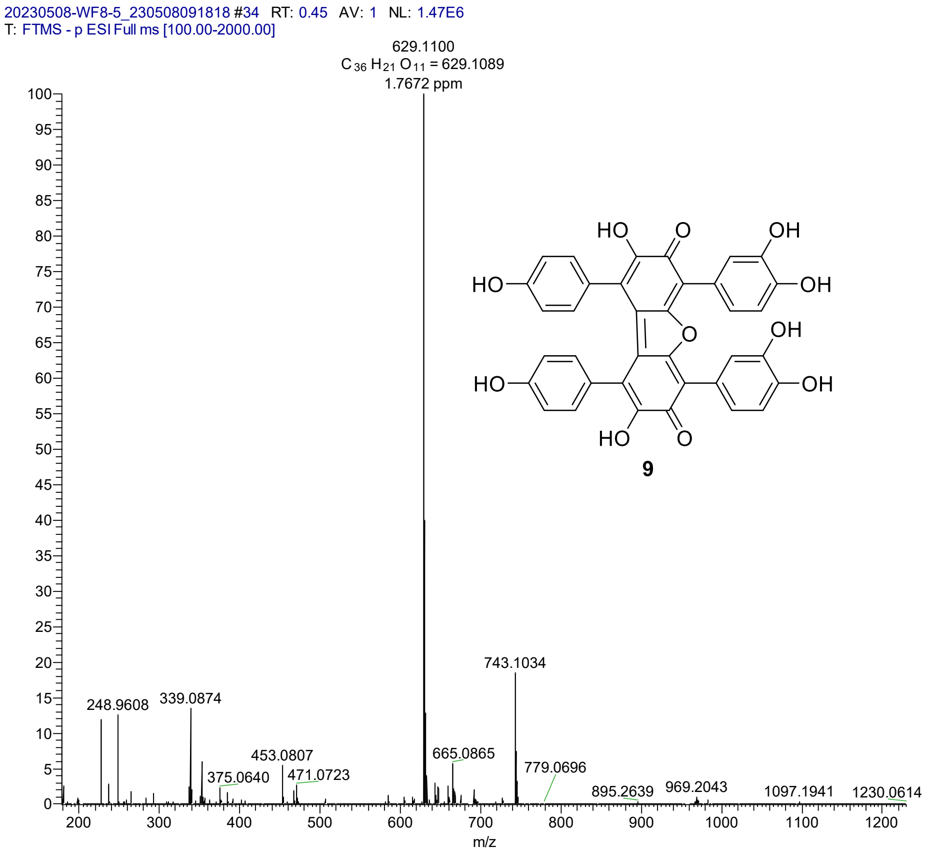

Figure S31. The ^{1}H (400 MHz) NMR spectrum of compound **10** in DMSO-d_6

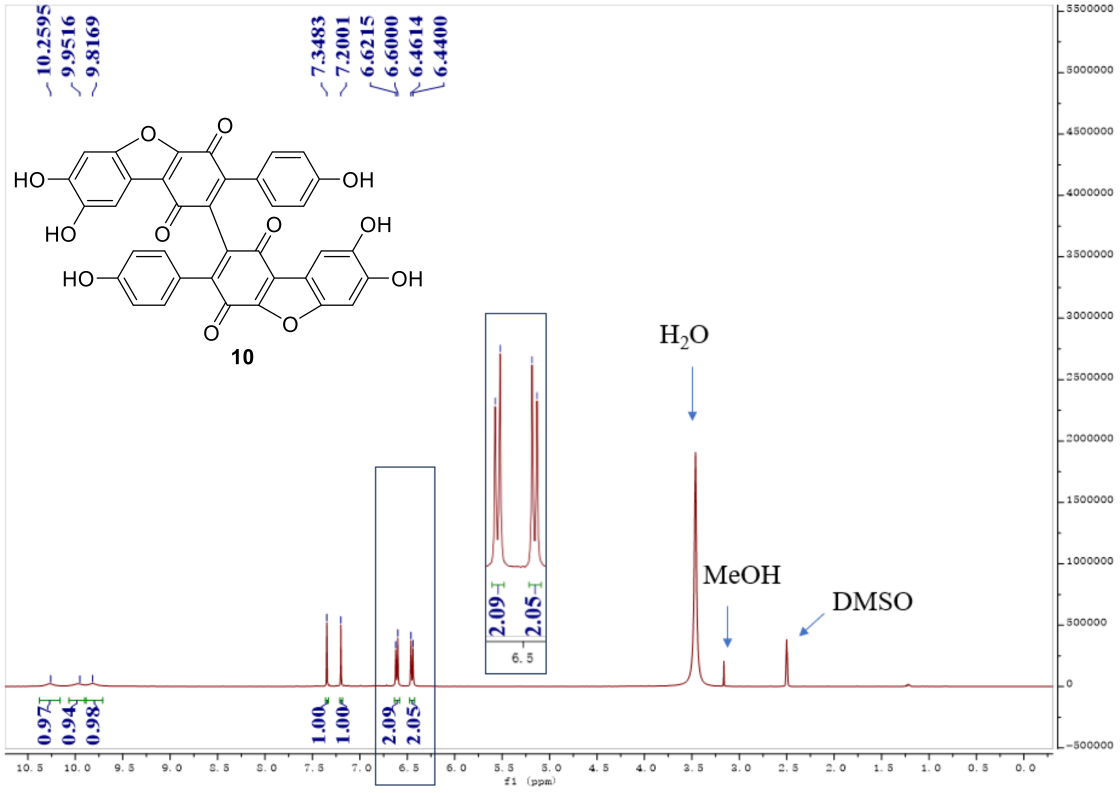

Figure S32. The ^{13}C (150 MHz) NMR spectrum of compound **10** in DMSO-d_6

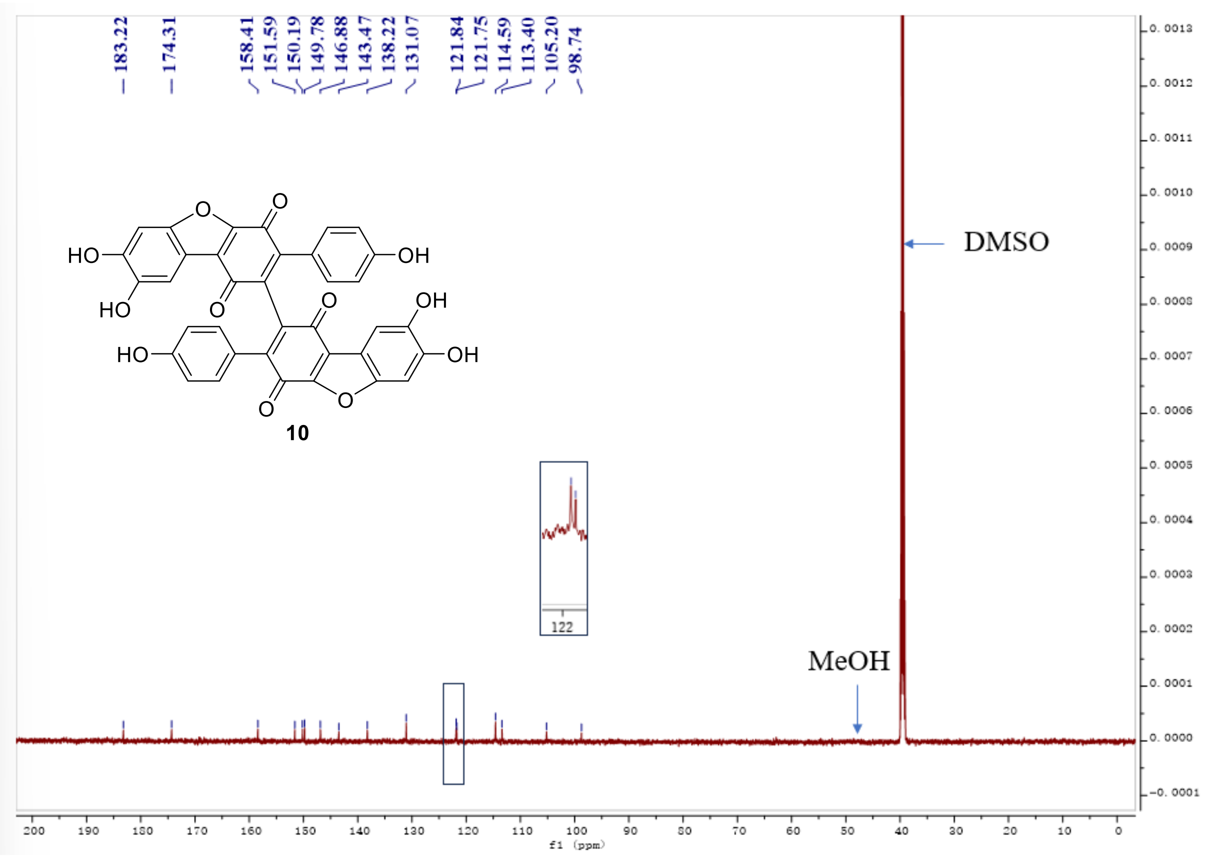

Figure S33. The HRESIMS spectrum of compound **10**

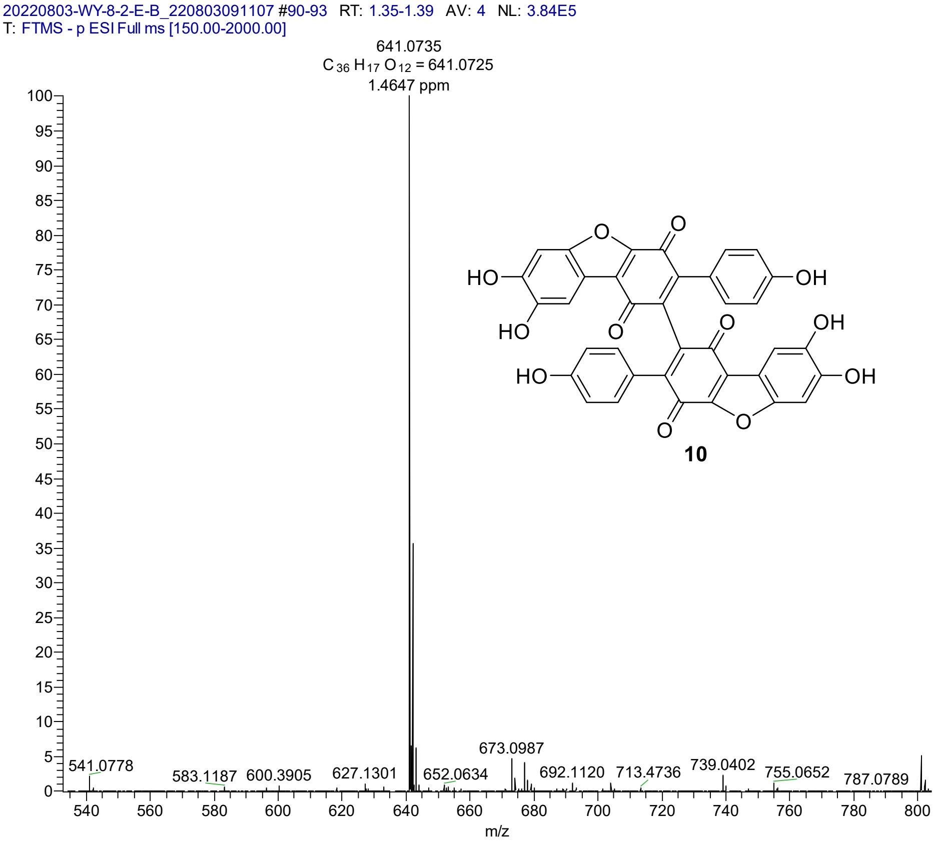

Figure S34. The ^{1}H (600 MHz) NMR spectrum of compound **11** in DMSO-d_6

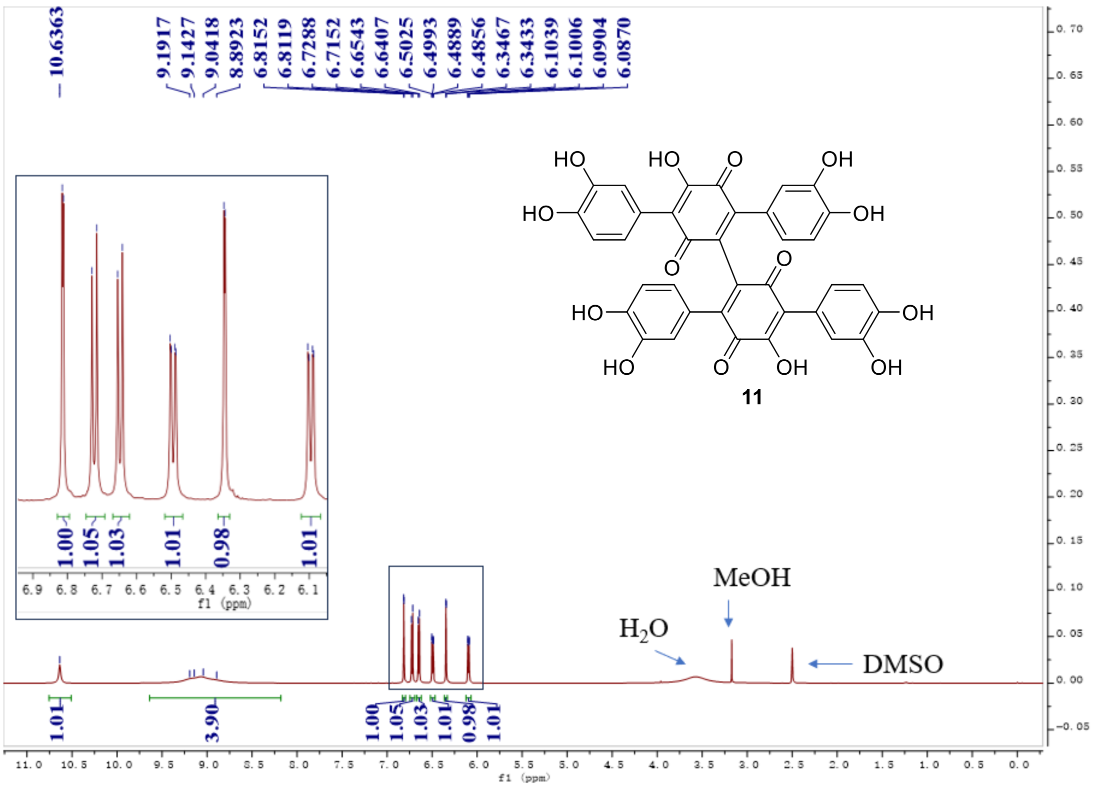

Figure S35. The ^{13}C (150 MHz) NMR spectrum of compound **11** in DMSO-d_6

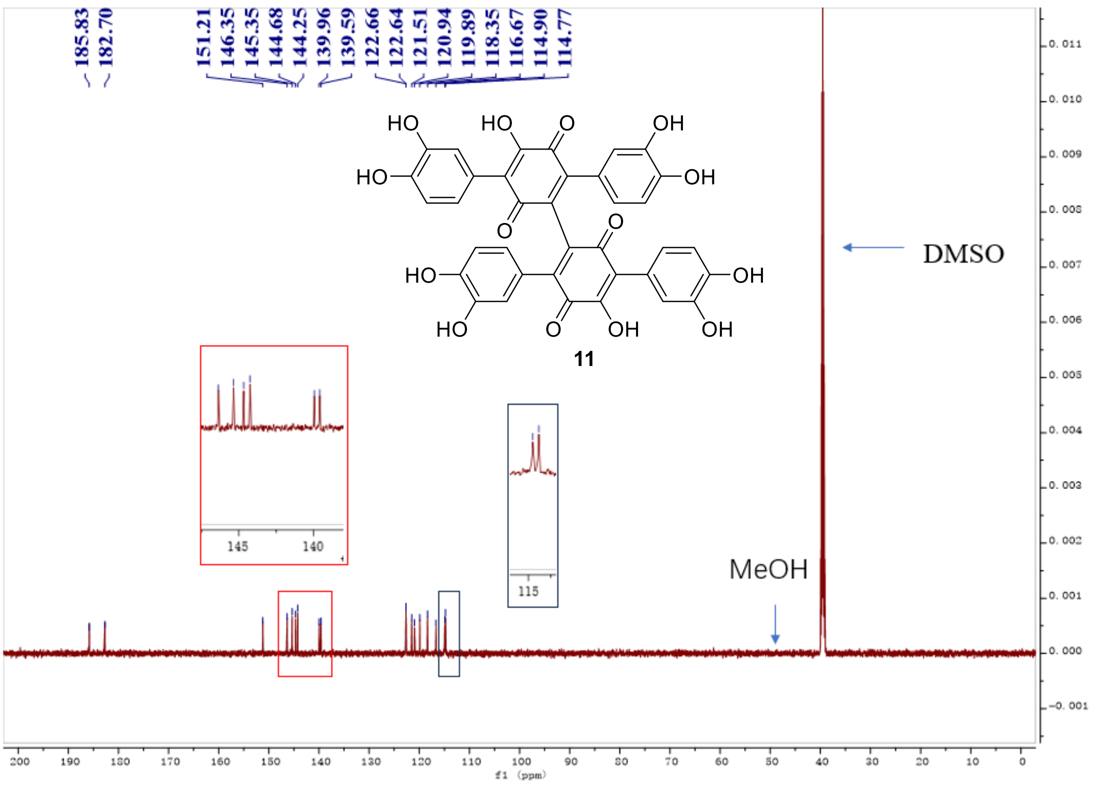

Figure S36. The HRESIMS spectrum of compound **11**

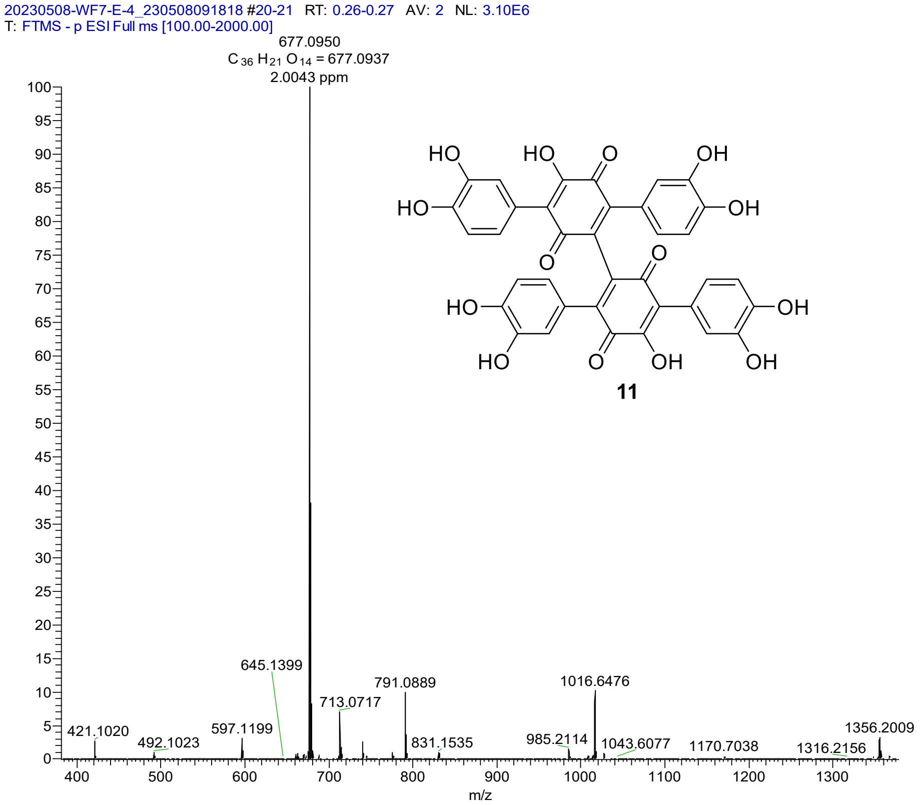

Figure S37. The ^{1}H (600 MHz) NMR spectrum of compound **12** in DMSO-d_6

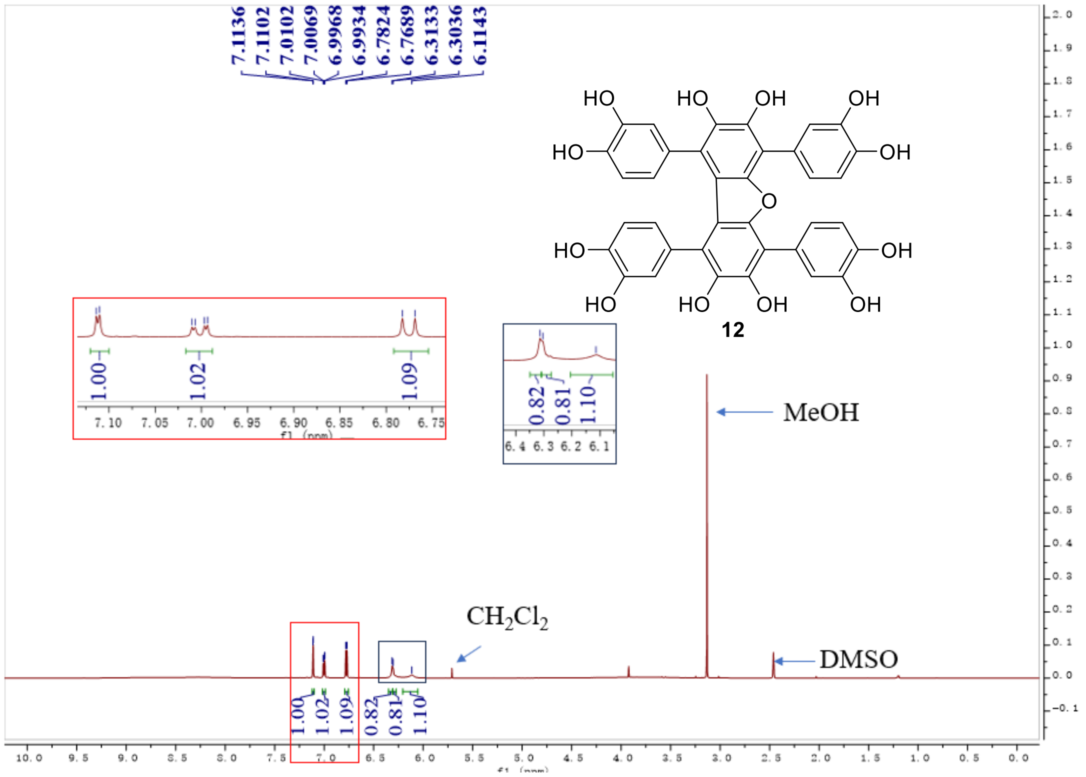

Figure S38. The ^{13}C (150 MHz) NMR spectrum of compound **12** in DMSO-d_6

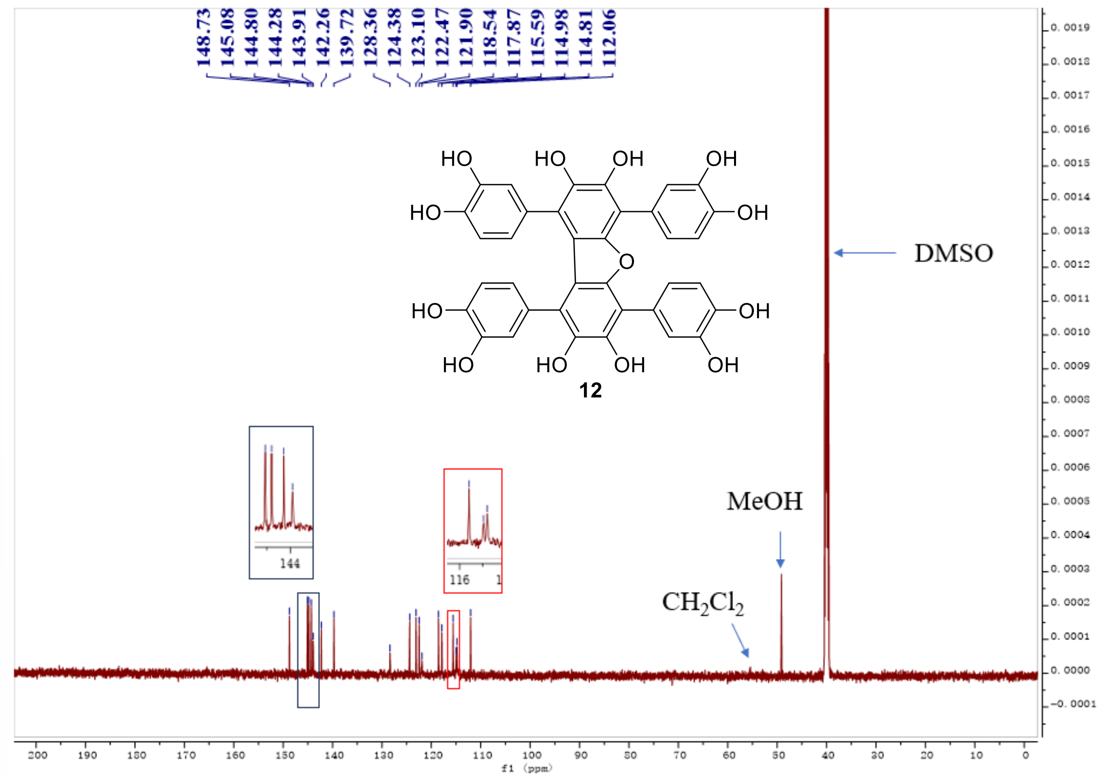

Figure S39. The HRESIMS spectrum of compound **12**

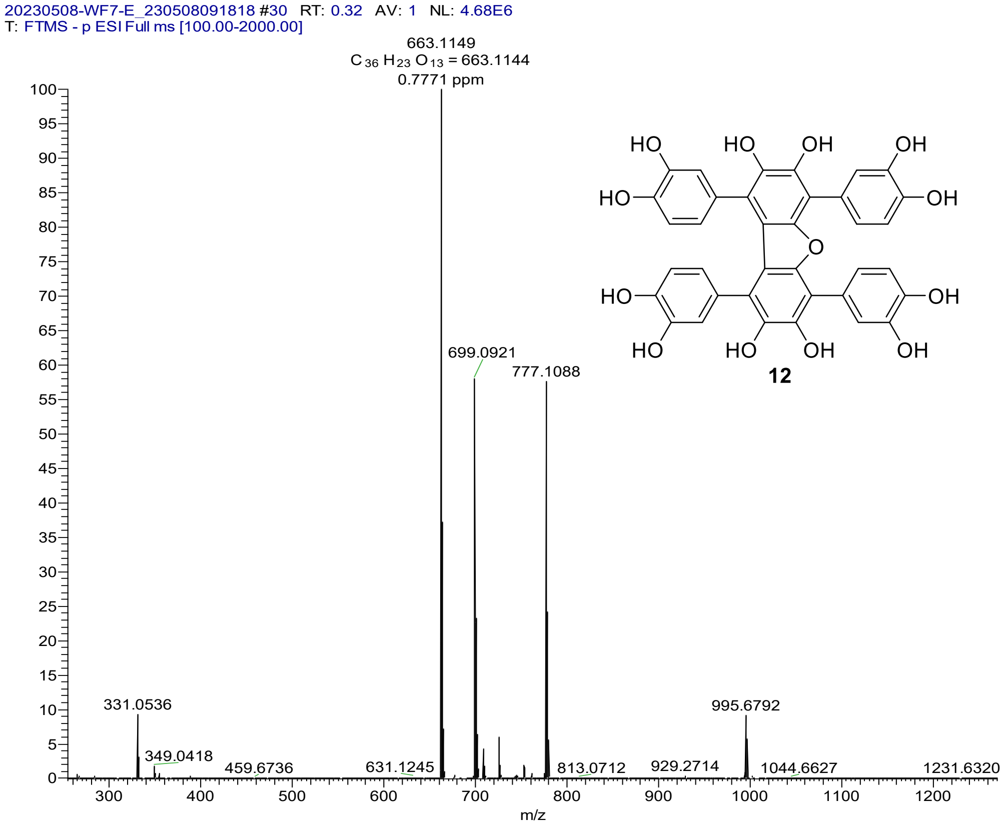

Figure S40. The ^{1}H (600 MHz) NMR spectrum of compound **13** in DMSO-d_6

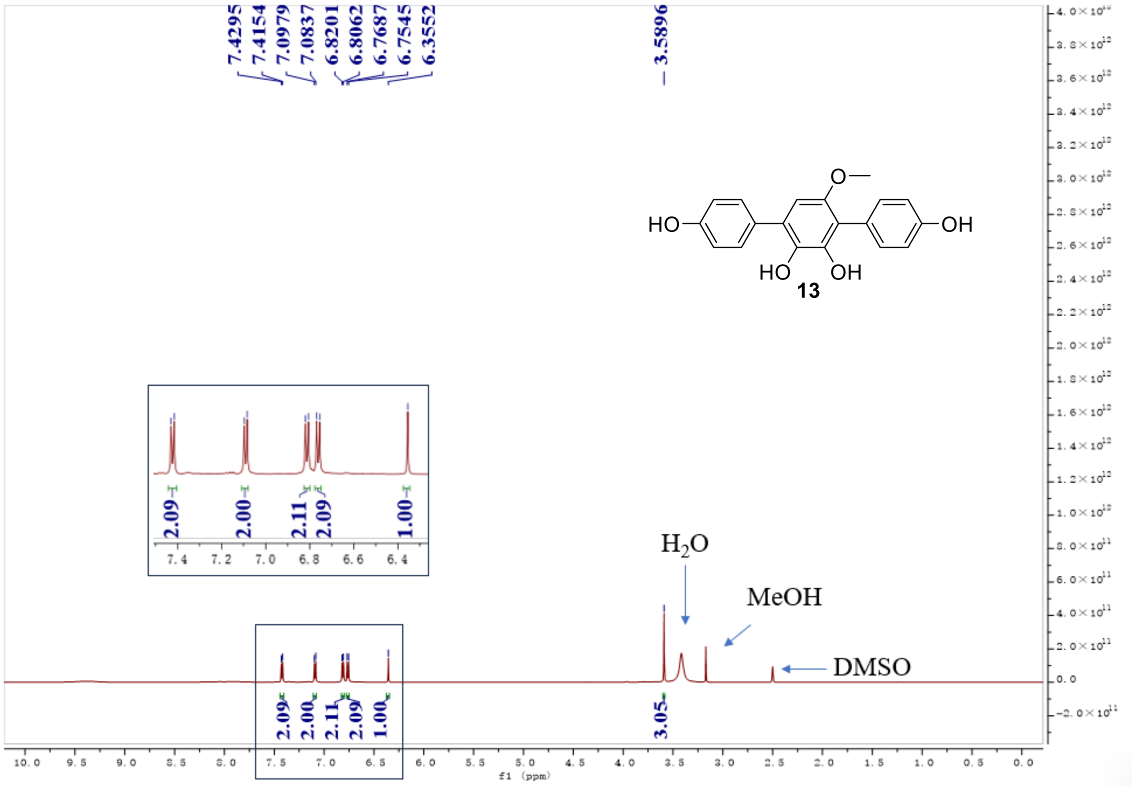

Figure S41. The ^{13}C (150 MHz) NMR spectrum of compound **13** in DMSO-d_6

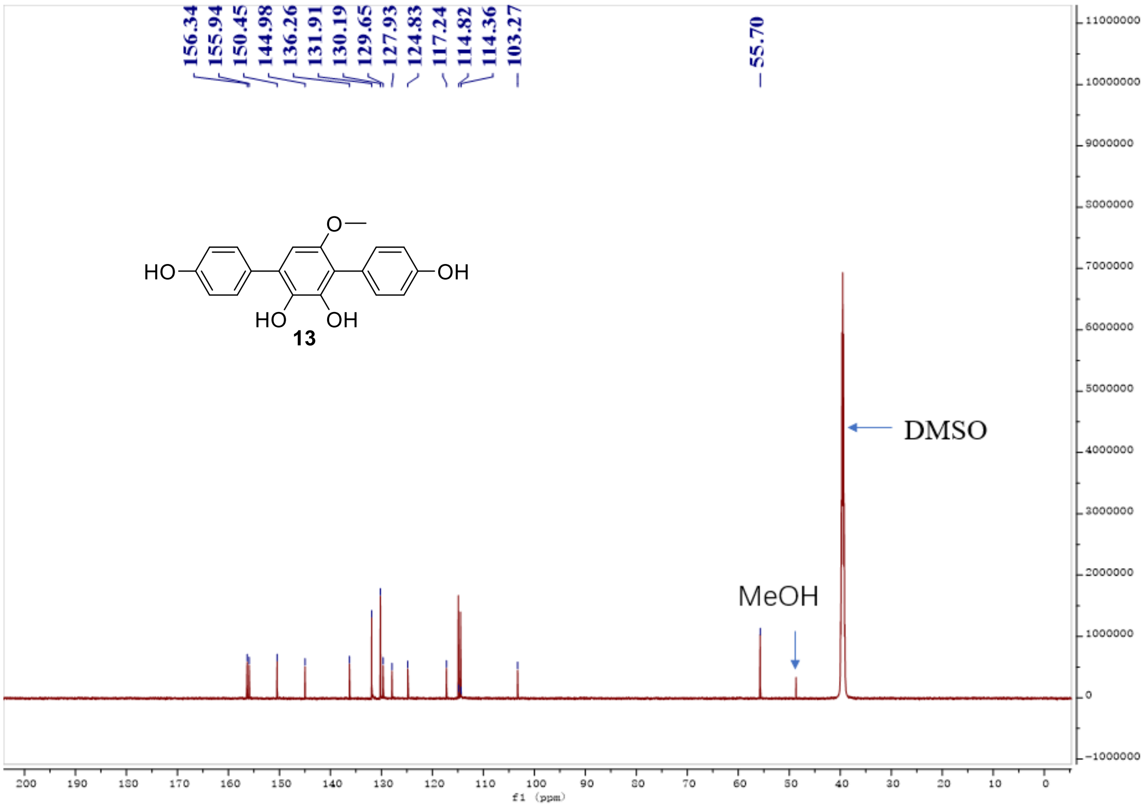

Figure S42. The LC-MS spectrum of compound **13** in DMSO-d_6

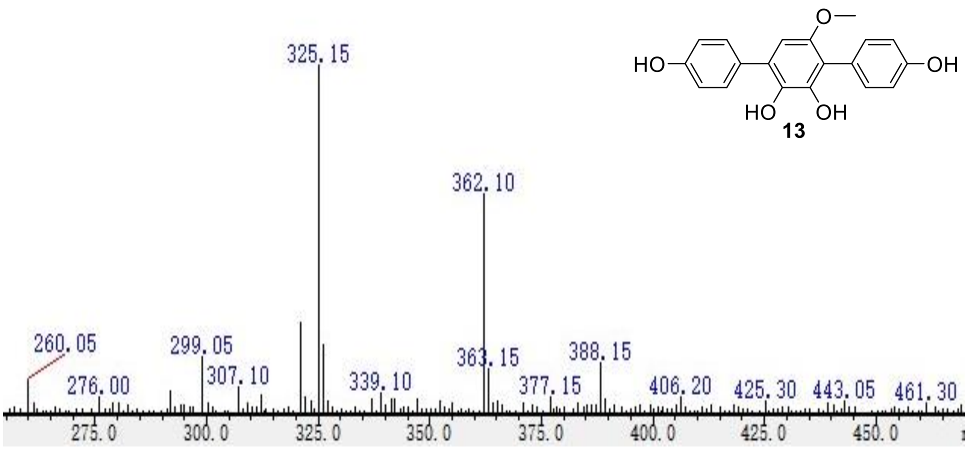

Figure S43. The HRESIMS spectrum of compound **14**

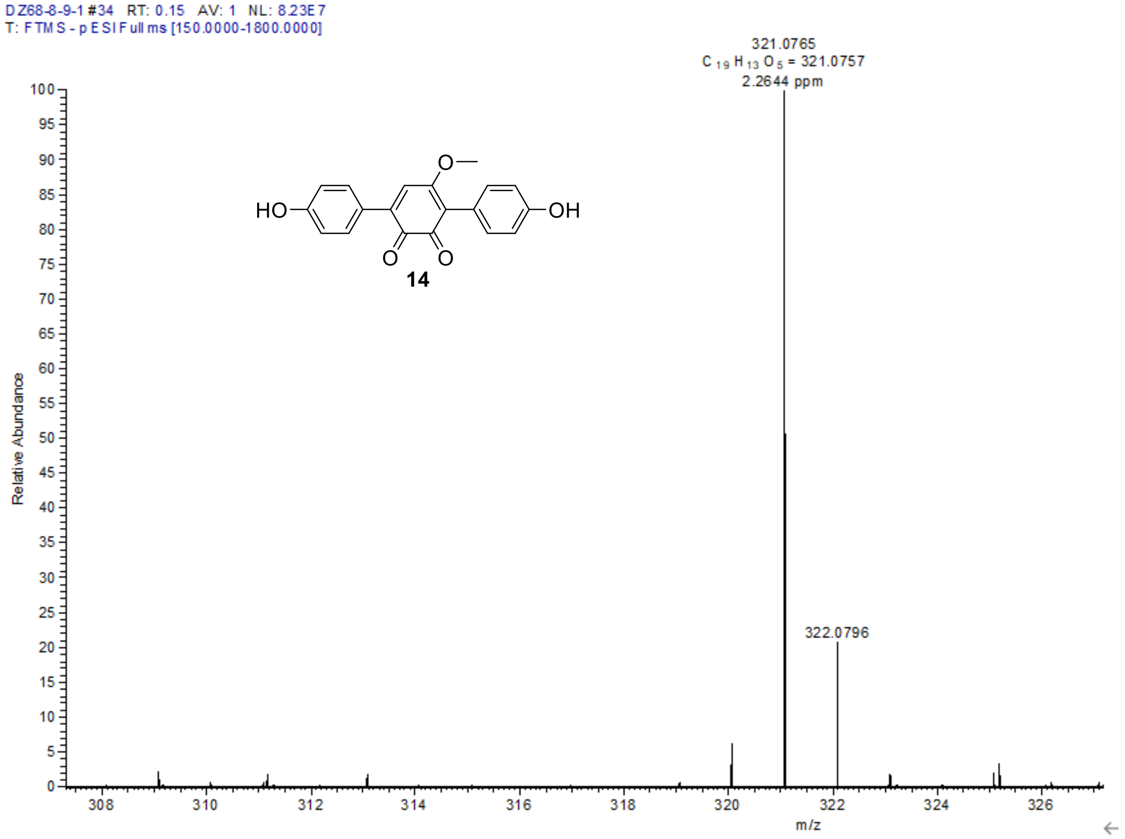

Figure S44. The ^{1}H (600 MHz) NMR spectrum of compound **14** in DMSO-d_6

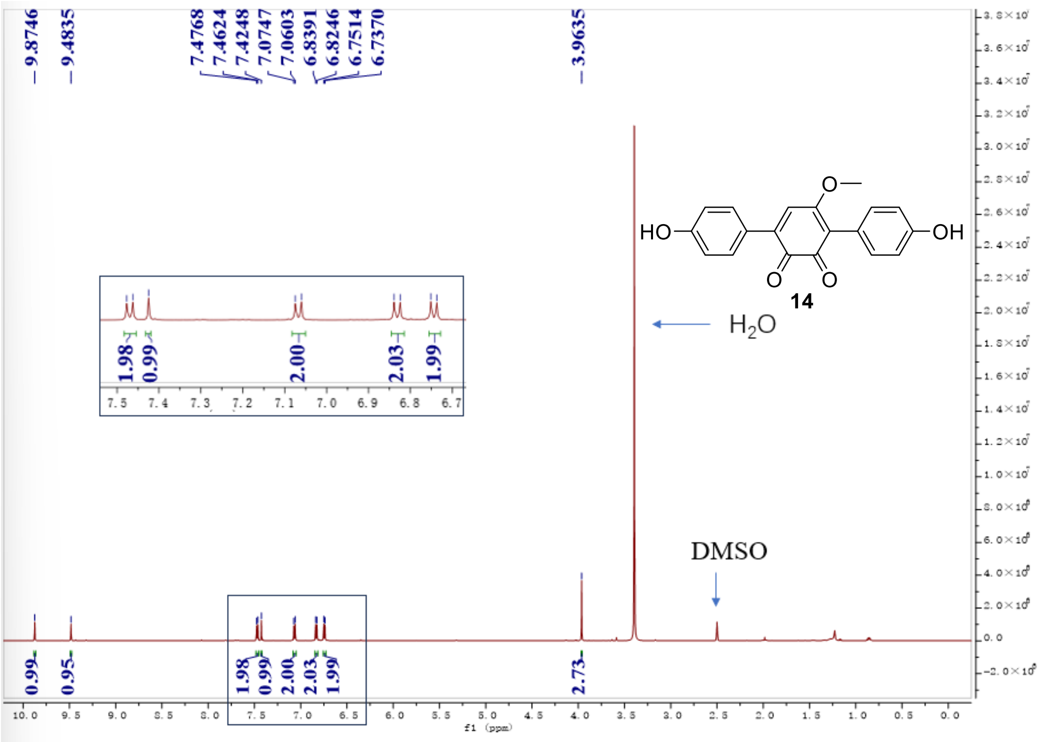

Figure S45. The ^{13}C (150 MHz) NMR spectrum of compound **14** in DMSO-d_6

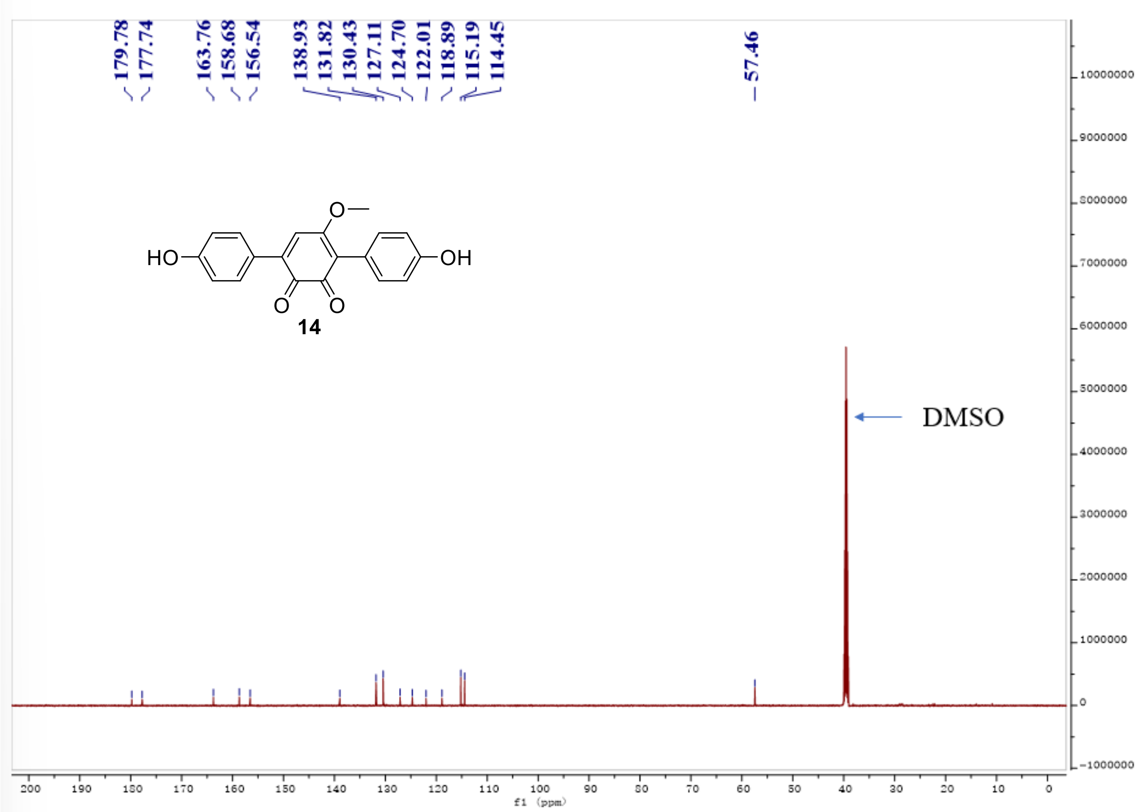

Figure S46. The ^{1}H (600 MHz) NMR spectrum of compound **15** in DMSO-d_6

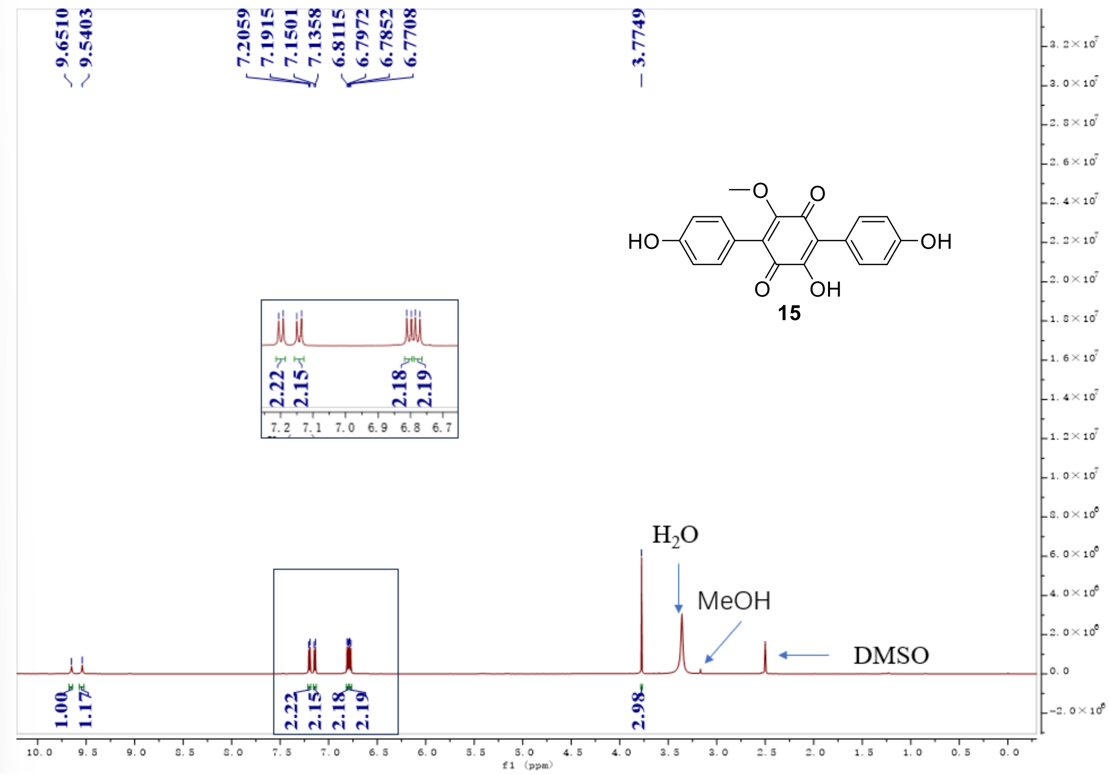

Figure S47. The ^{13}C (150 MHz) NMR spectrum of compound **15** in DMSO-d_6

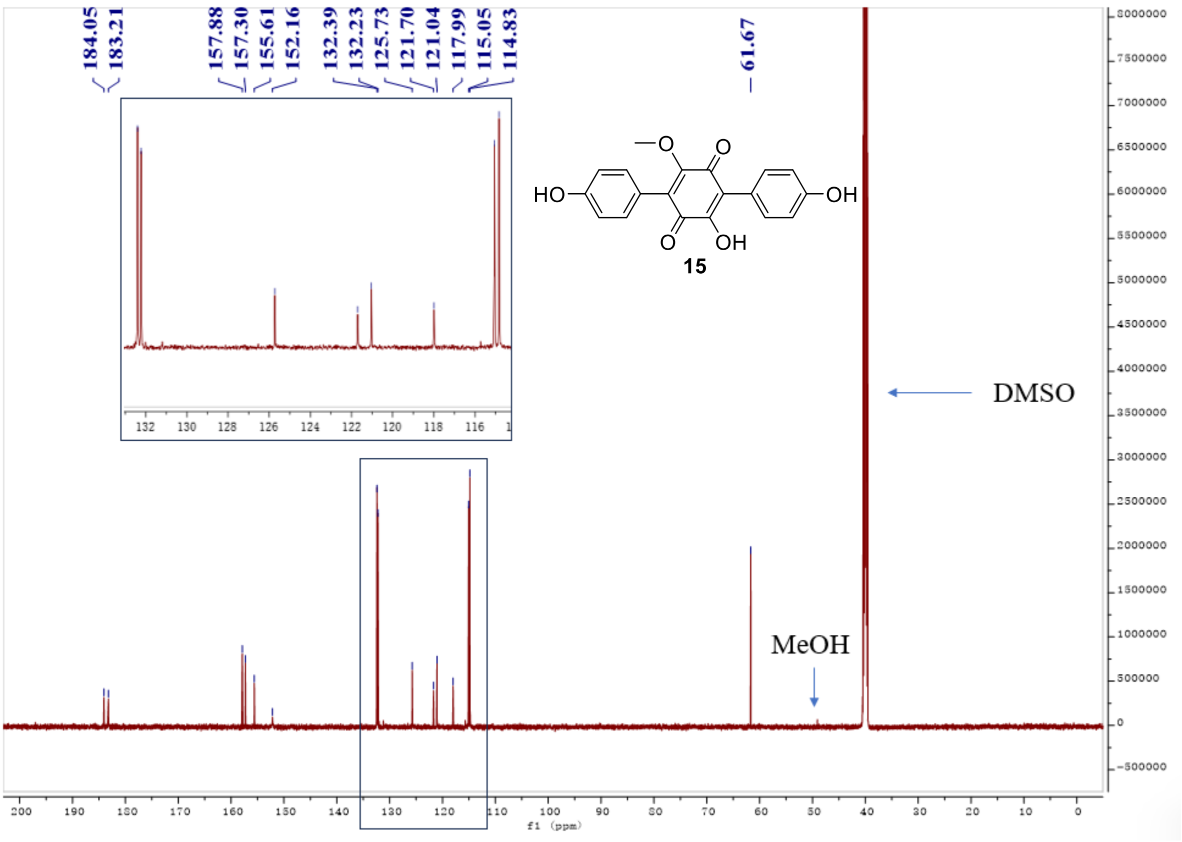

Figure S48. The LC-MS spectrum of compound **15** in DMSO-d_6

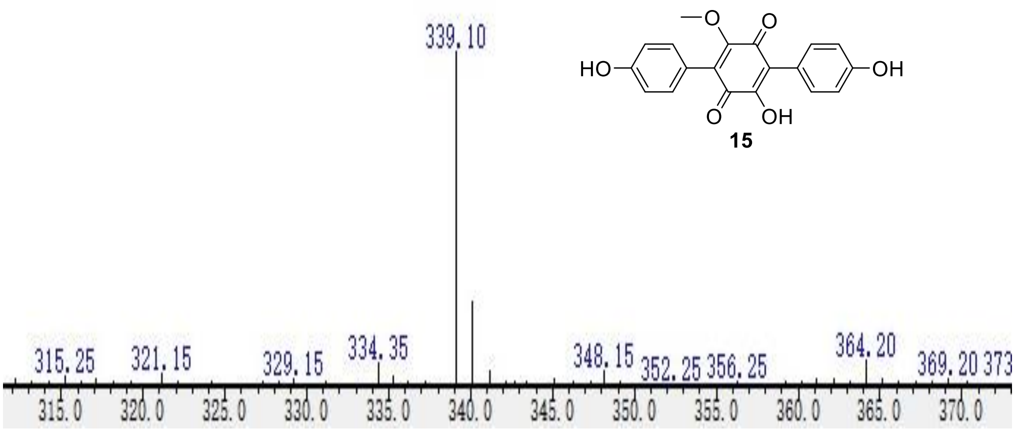

Figure S49. The ^{1}H (600 MHz) NMR spectrum of compound **16** in DMSO-d_6

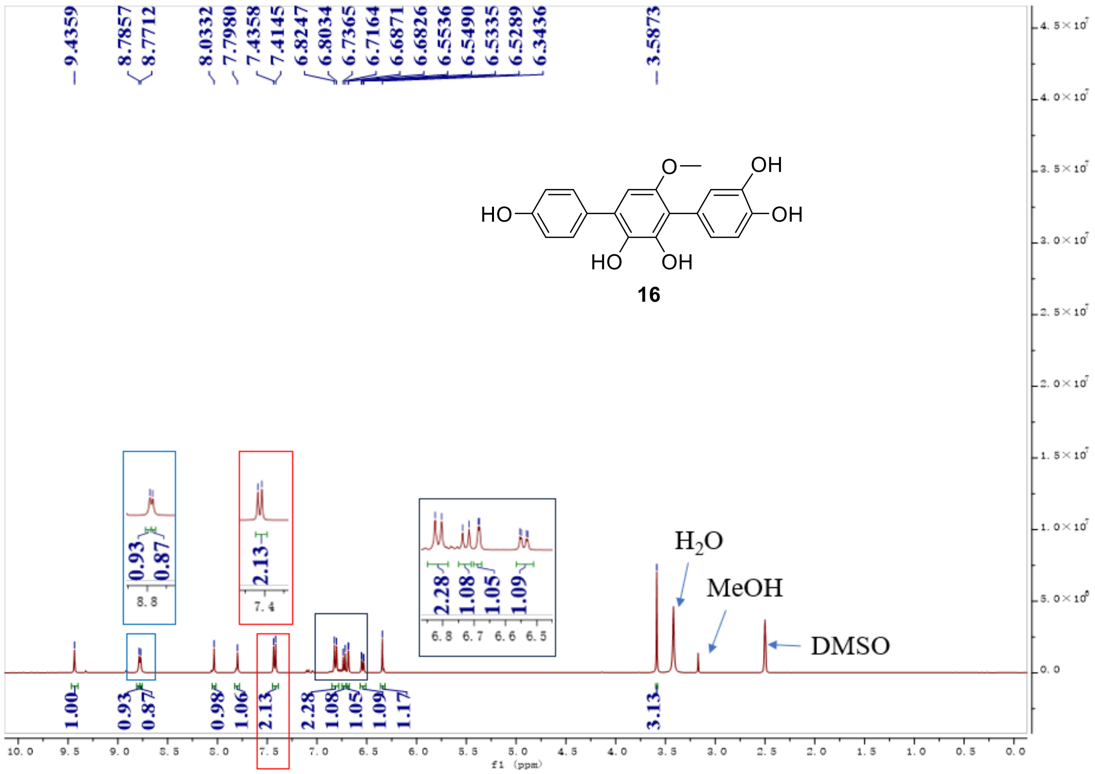

Figure S50. The ^{13}C (150 MHz) NMR spectrum of compound **16** in DMSO-d_6

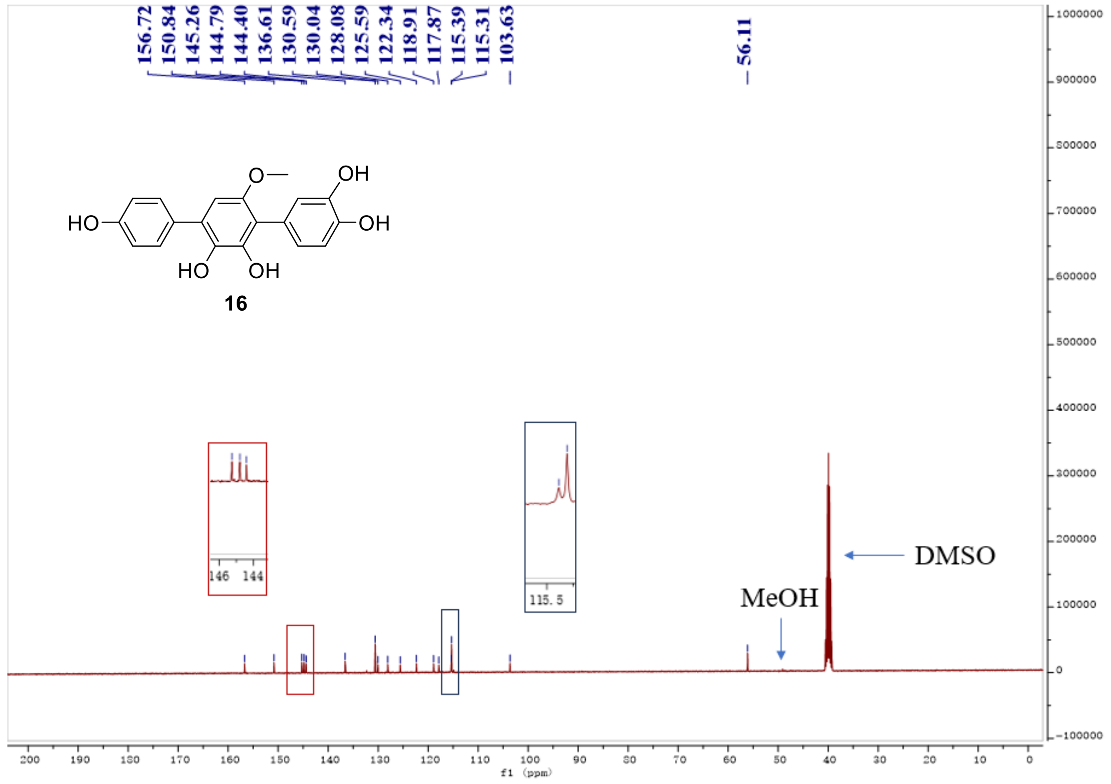

Figure S51. The HRESIMS spectrum of compound **16**

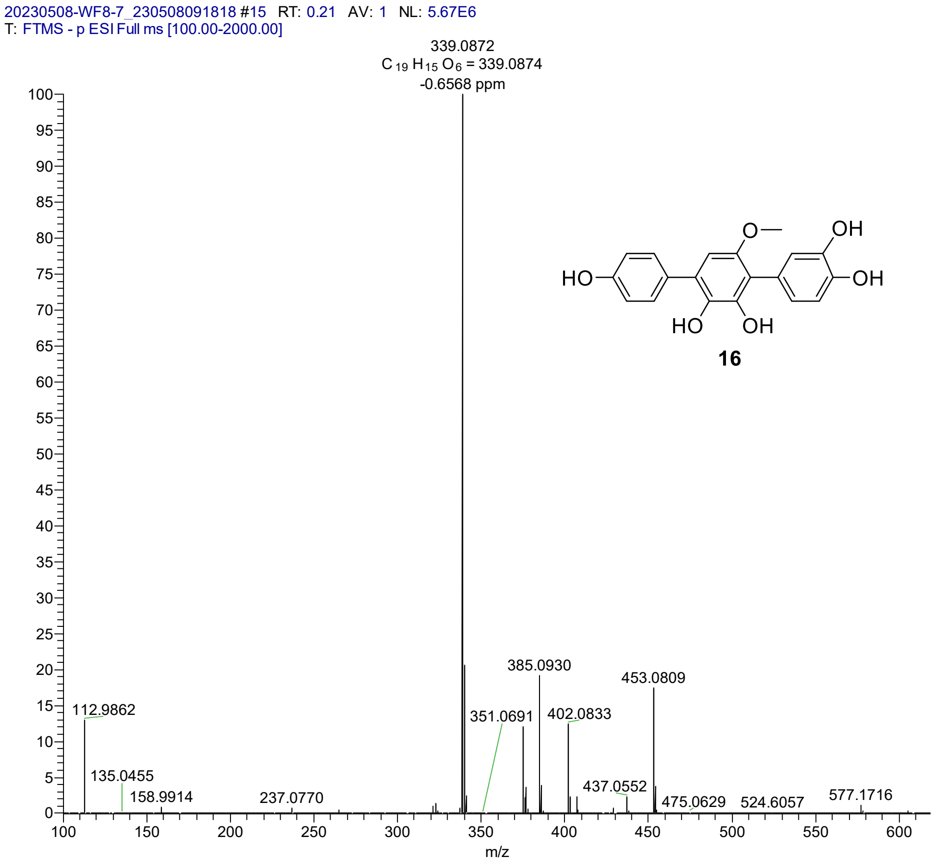

Figure S52. The ^{1}H (600 MHz) NMR spectrum of compound **17** in DMSO-d_6

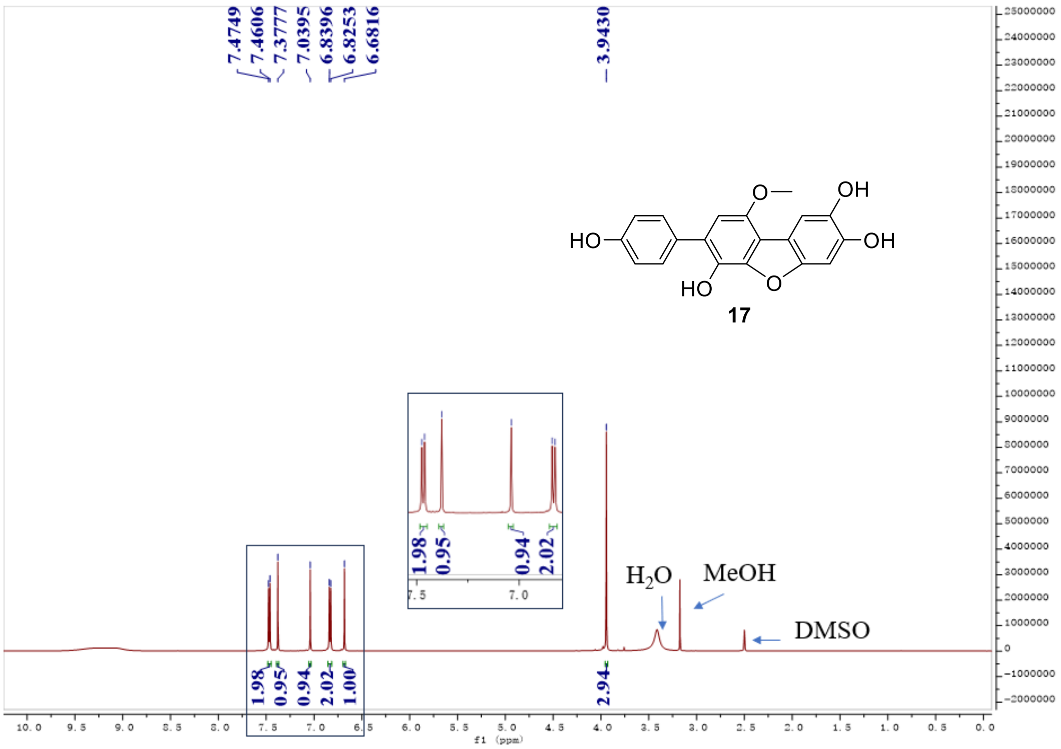

Figure S53. The ^{13}C (150 MHz) NMR spectrum of compound **17** in DMSO-d_6

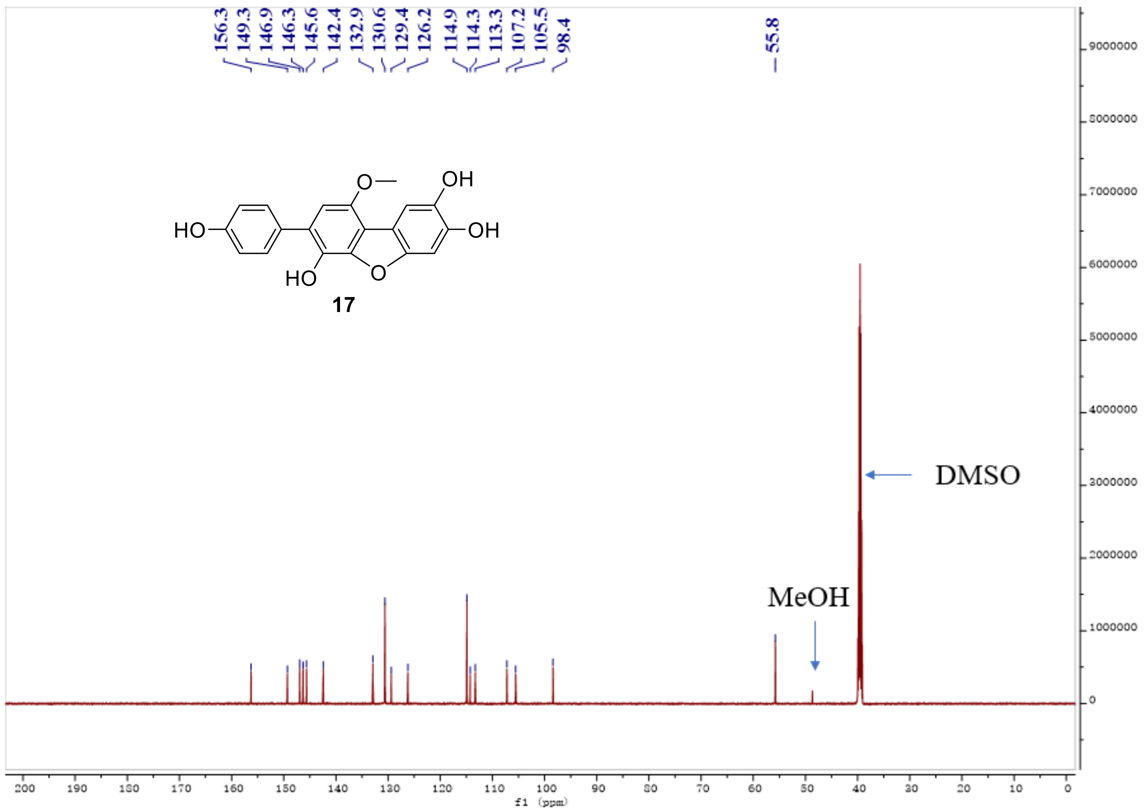

Figure S54. The HRESIMS spectrum of compound **17**

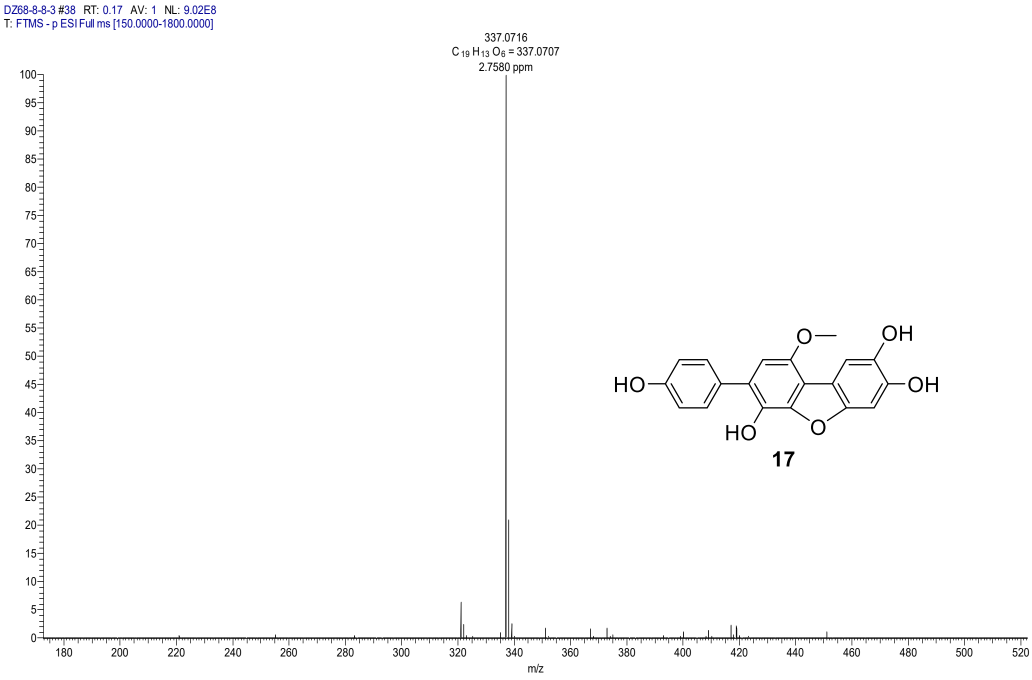

Figure S55. The ^{1}H (600 MHz) NMR spectrum of compound **18** in DMSO-d_6

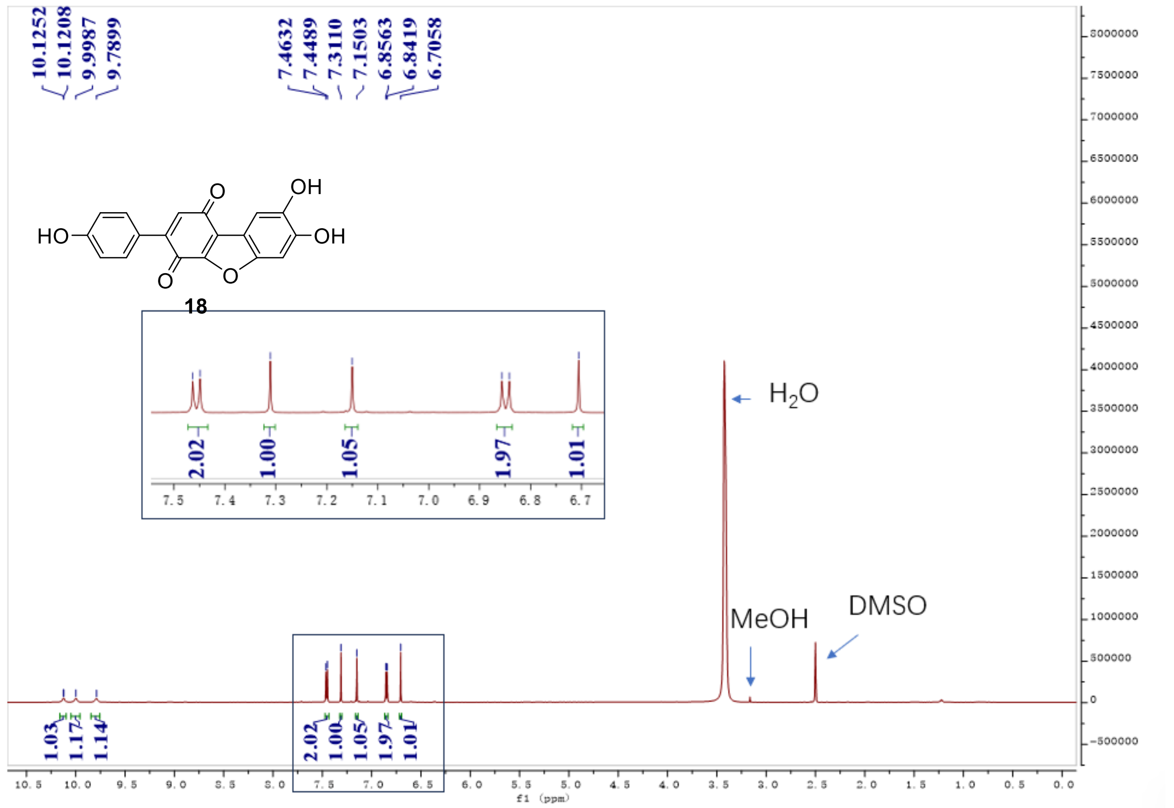

Figure S56. The ^{13}C (150 MHz) NMR spectrum of compound **18** in DMSO-d_6

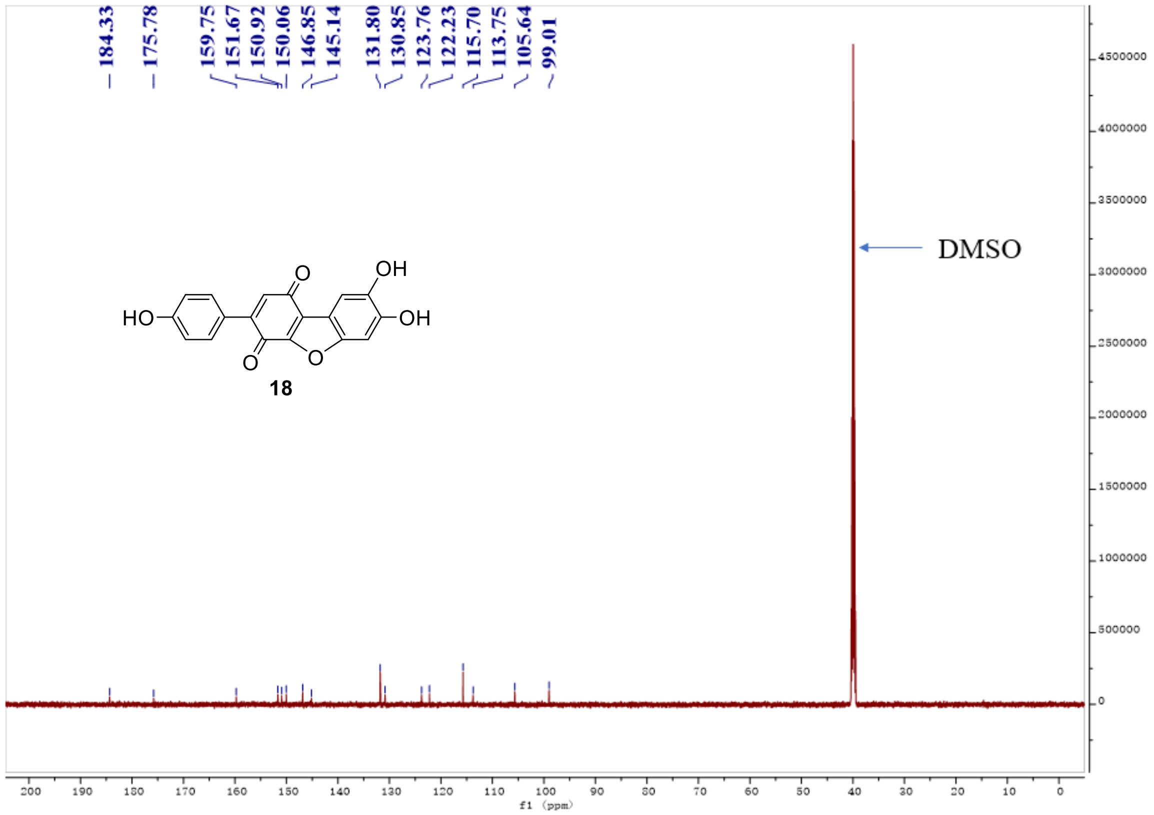

Figure S57. The HRESIMS spectrum of compound **18**

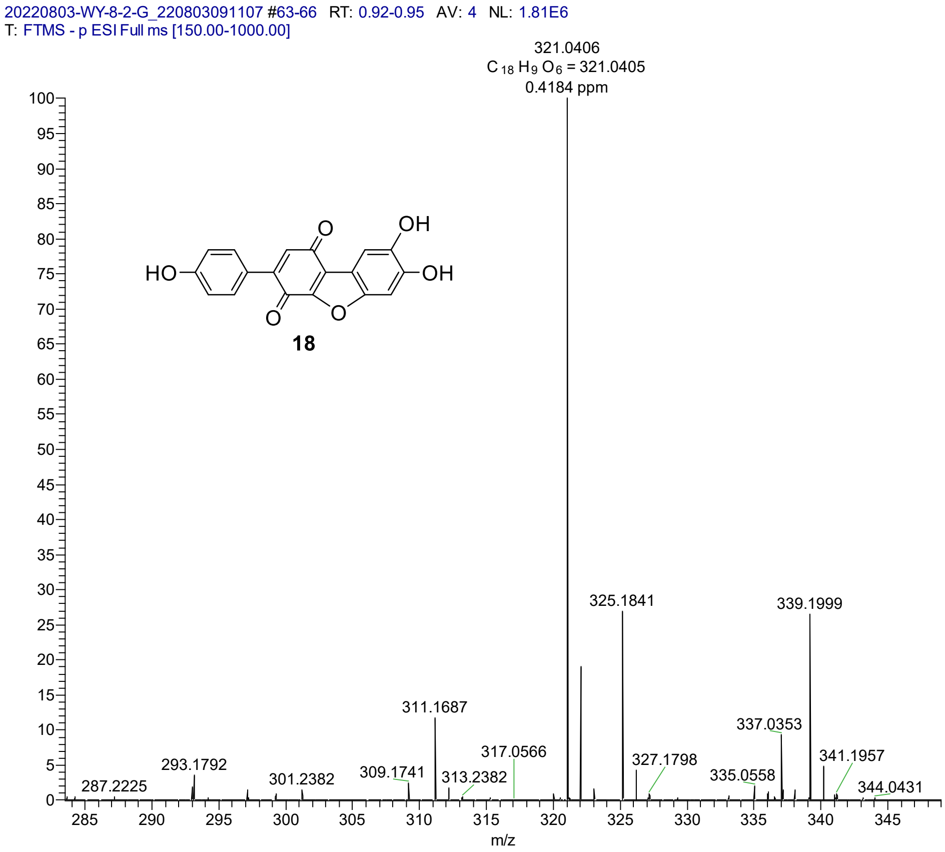

Figure S58. The ^{1}H (600 MHz) NMR spectrum of compound **19** in DMSO-*d*6

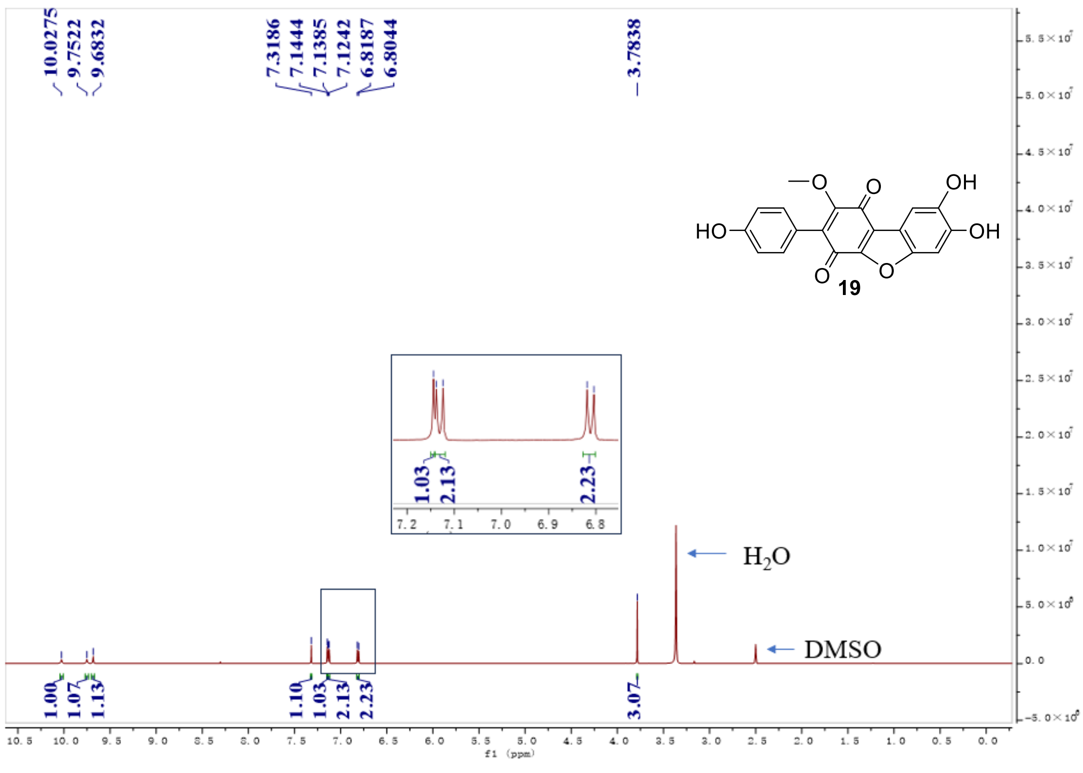

Figure S59. The ^{13}C (150 MHz) NMR spectrum of compound **19** in DMSO-*d*6

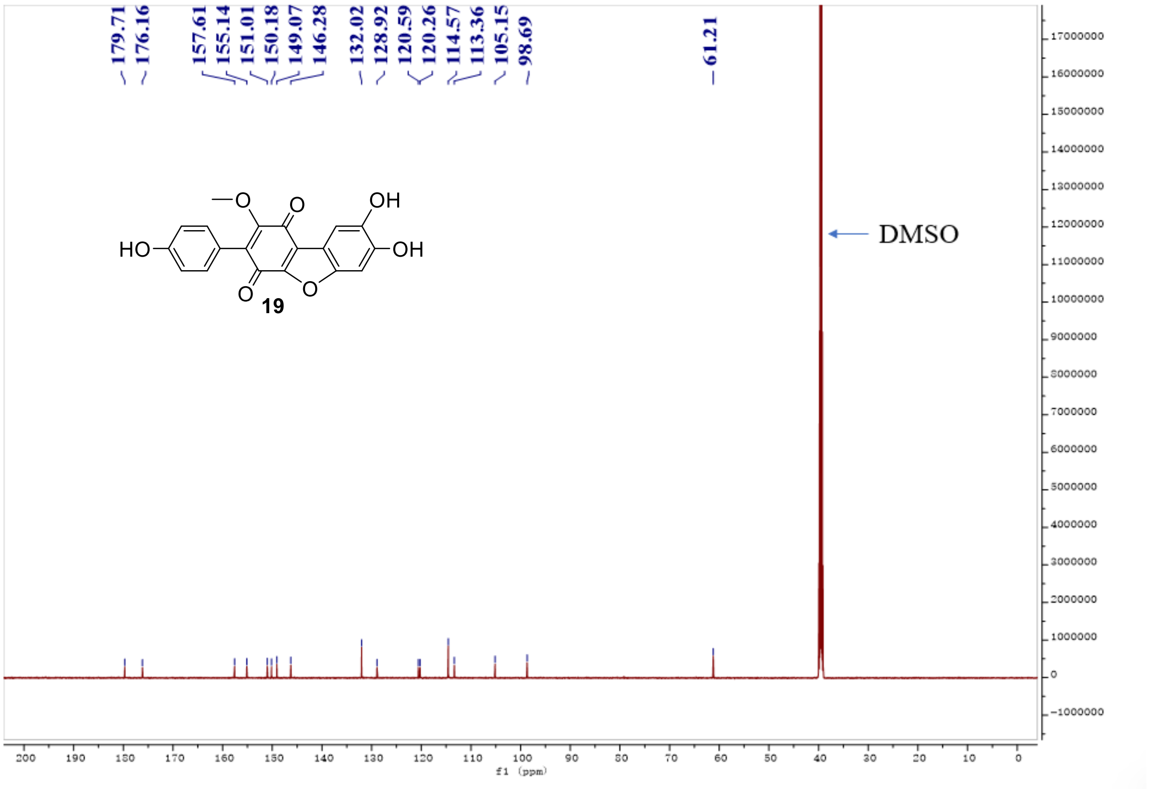

Figure S60. The HRESIMS spectrum of compound **19**

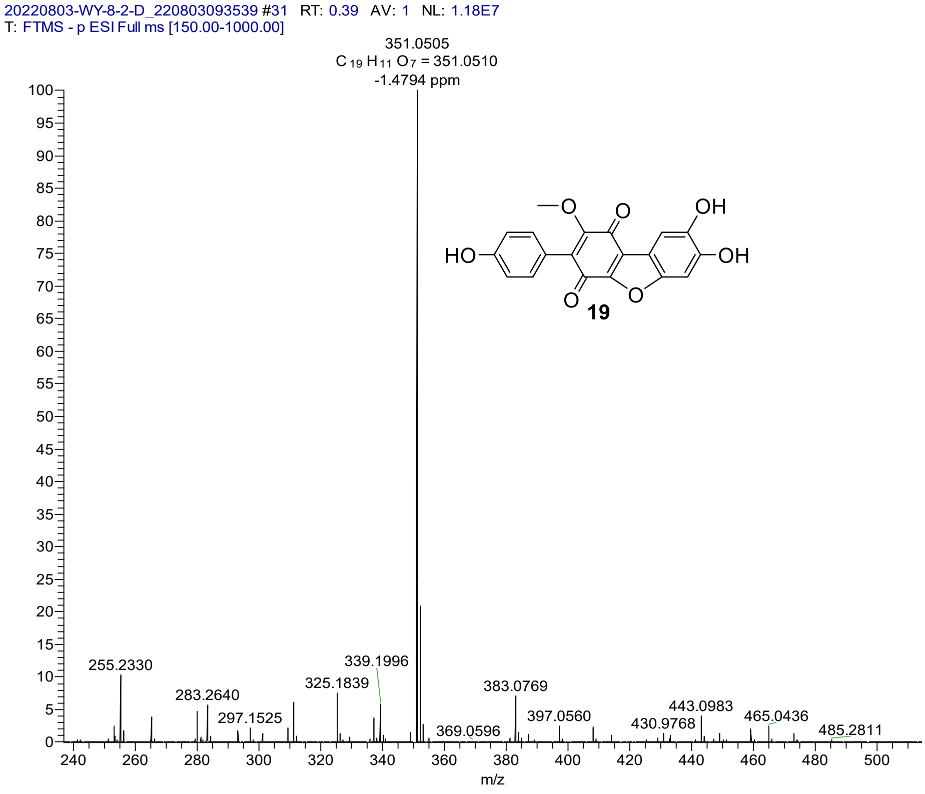

Figure S61. The ^{1}H (600 MHz) NMR spectrum of compound **20** in DMSO-d_6

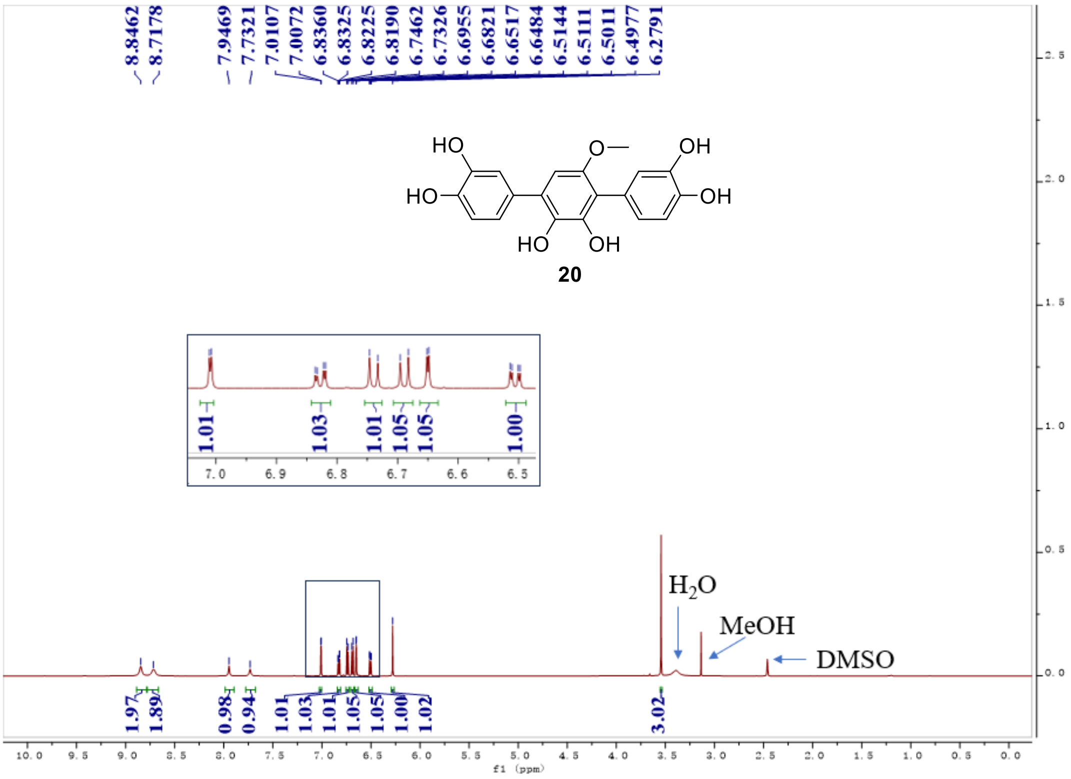

Figure S62. The ^{13}C (150 MHz) NMR spectrum of compound **20** in DMSO-d_6

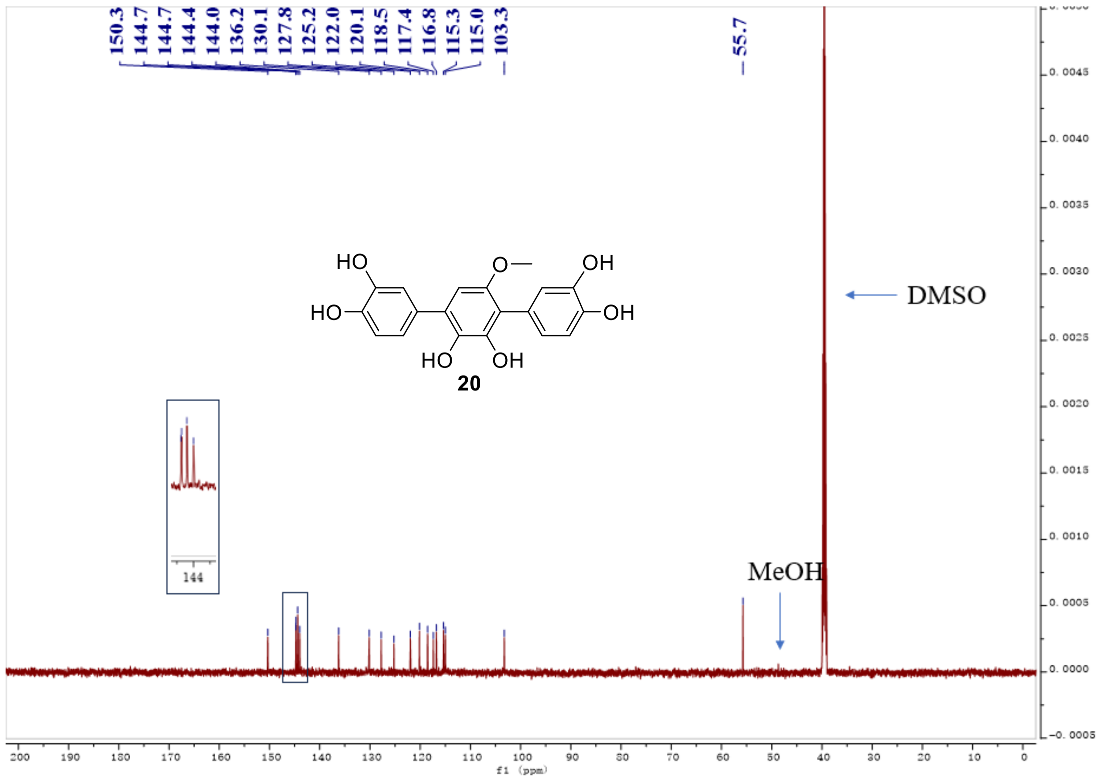

Figure S63. The HRESIMS spectrum of compound **20**

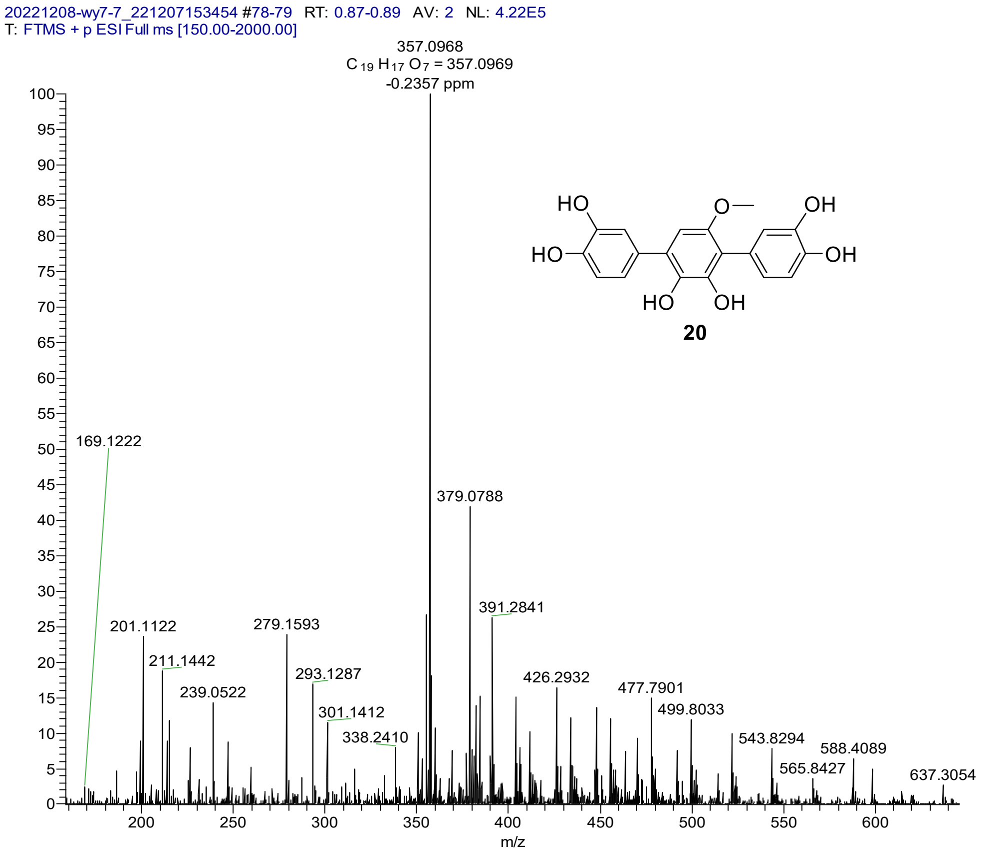

Figure S64. The ^{1}H (600 MHz) NMR spectrum of compound **21** in DMSO-d_6

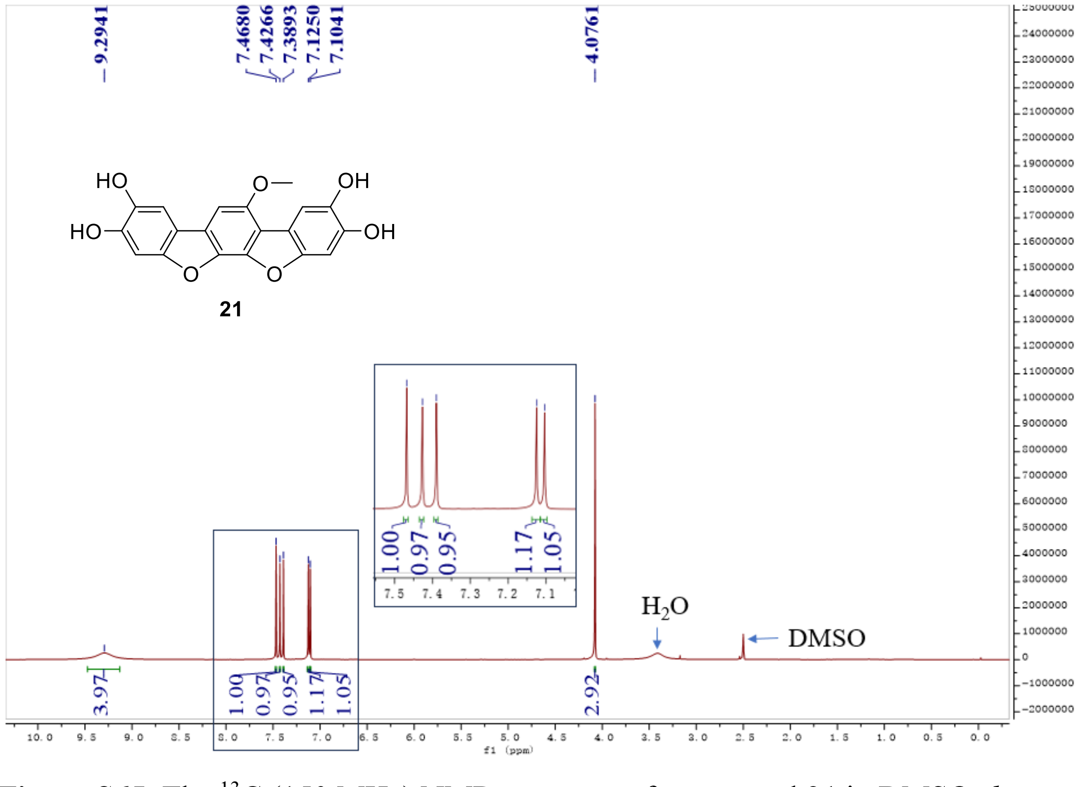

Figure S65. The ^{13}C (150 MHz) NMR spectrum of compound **21** in DMSO-d_6

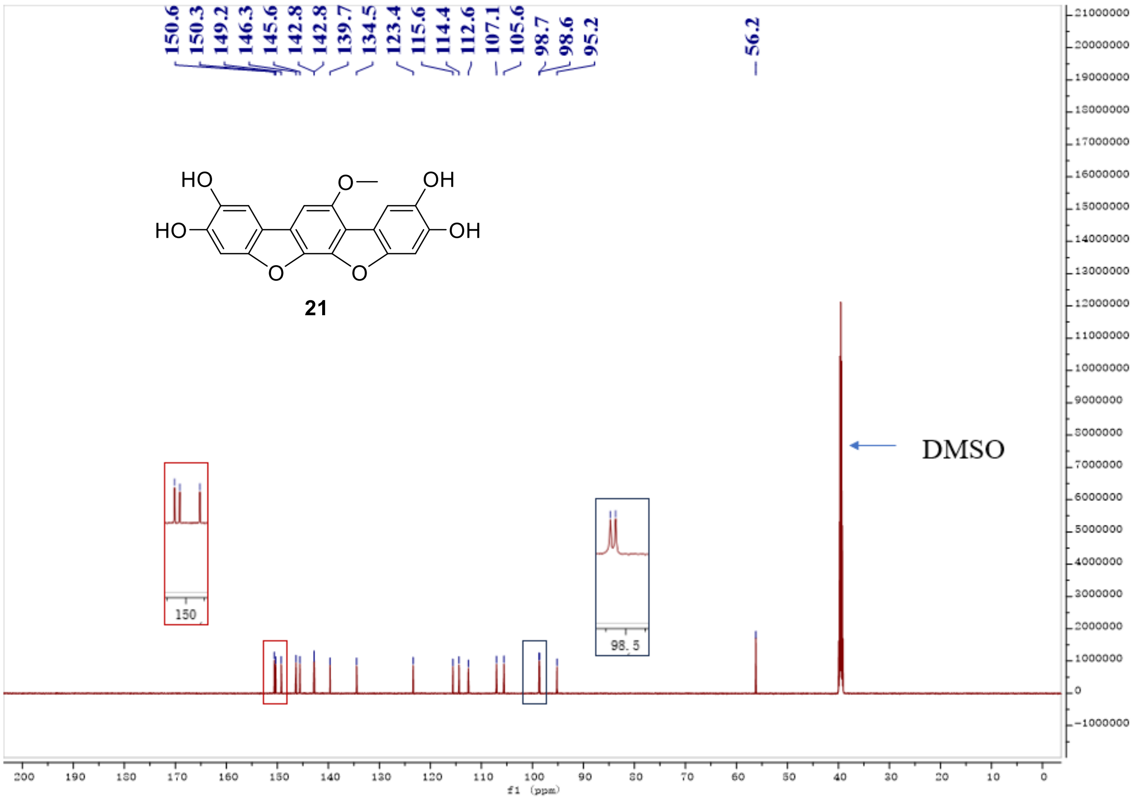

Figure S66. The HRESIMS spectrum of compound **21**

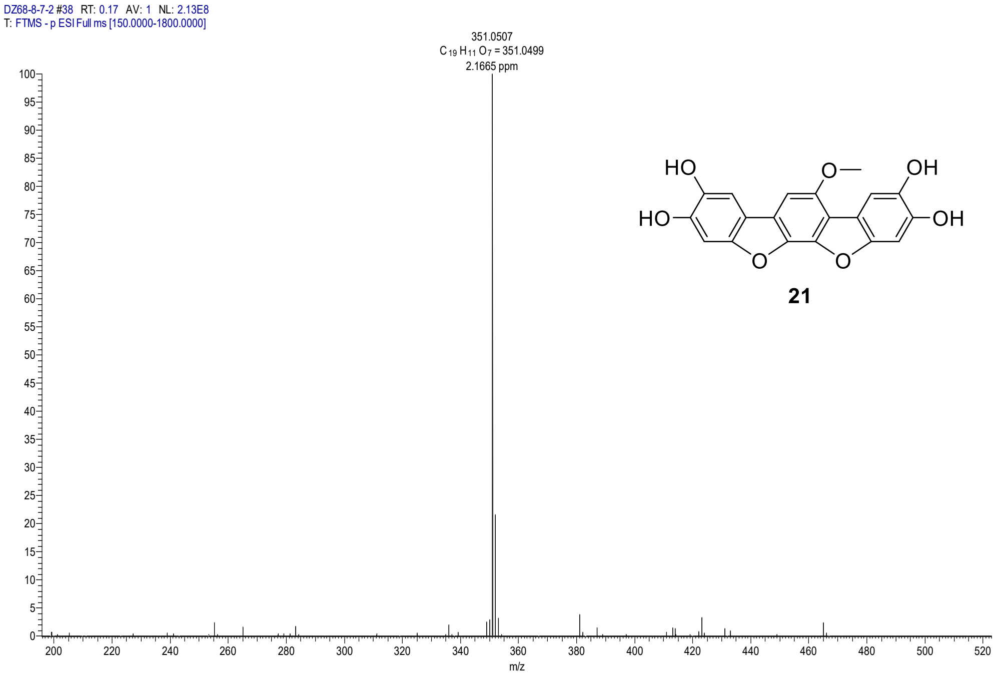

Figure S67. The ^{1}H (600 MHz) NMR spectrum of compound **22** in DMSO-*d*6

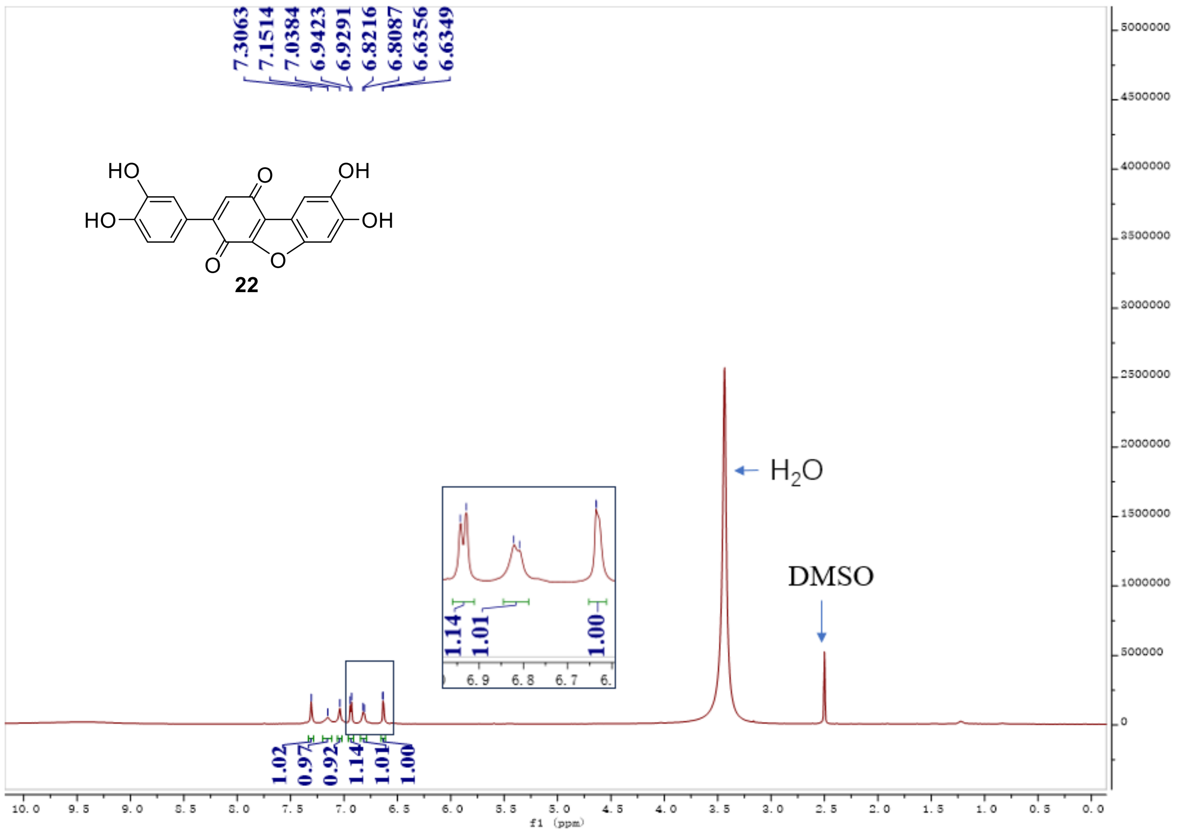

Figure S68. The ^{13}C (150 MHz) NMR spectrum of compound **22** in DMSO-*d*6

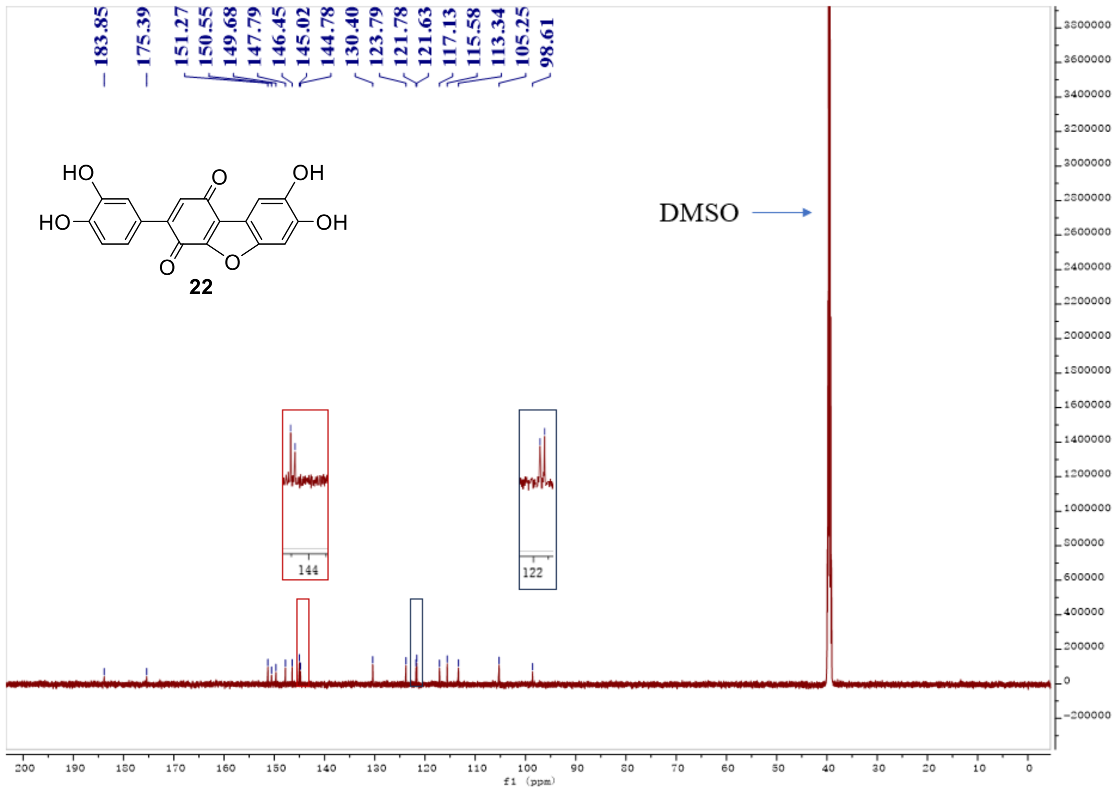

Figure S69. The HRESIMS spectrum of compound **22**

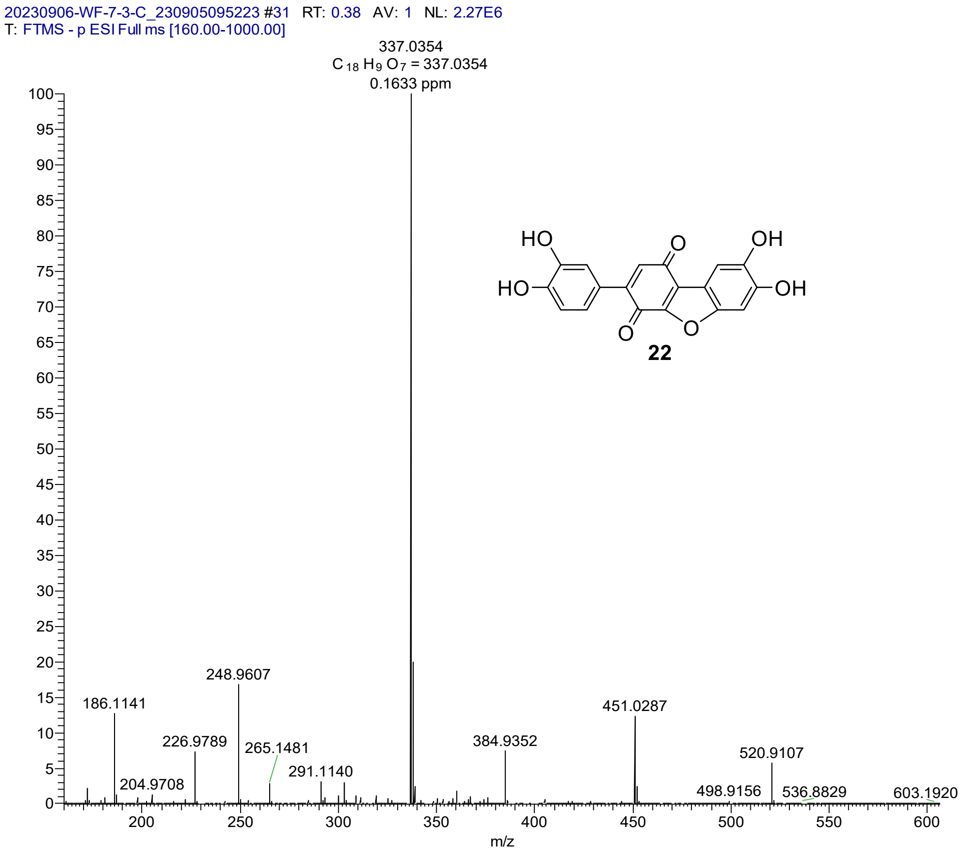

Figure S70. The HPLC spectrum of compound **22**

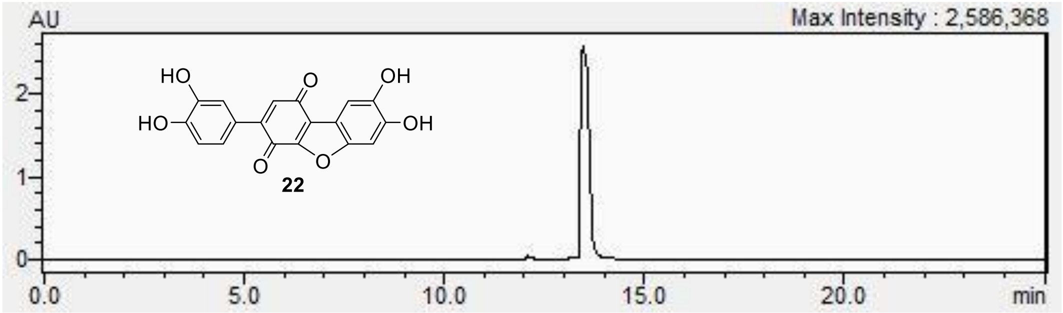

Supplement: Supplementary file 1 [file ijms-27-02726-s001.zip › ijms-4154635-supplementary.pdf]
